# YAP/TAZ and ATF4 drive resistance to Sorafenib in hepatocellular carcinoma by preventing ferroptosis

**DOI:** 10.15252/emmm.202114351

**Published:** 2021-10-19

**Authors:** Ruize Gao, Ravi K R Kalathur, Mairene Coto‐Llerena, Caner Ercan, David Buechel, Song Shuang, Salvatore Piscuoglio, Michael T Dill, Fernando D Camargo, Gerhard Christofori, Fengyuan Tang

**Affiliations:** ^1^ Department of Biomedicine University of Basel Basel Switzerland; ^2^ Institute of Pathology University Hospital Basel Basel Switzerland; ^3^ Friedrich Miescher Institute for Biomedical Research Basel Switzerland; ^4^ Stem Cell Program Boston Children's Hospital Boston MA USA; ^5^ Department of Stem Cell and Regenerative Biology Harvard University Cambridge MA USA

**Keywords:** ATF4, ferroptosis, Hippo signaling, liver cancer, YAP/TAZ, Autophagy & Cell Death, Cancer, Digestive System

## Abstract

Understanding the mechanisms underlying evasive resistance in cancer is an unmet medical need to improve the efficacy of current therapies. In this study, a combination of shRNA‐mediated synthetic lethality screening and transcriptomic analysis revealed the transcription factors YAP/TAZ as key drivers of Sorafenib resistance in hepatocellular carcinoma (HCC) by repressing Sorafenib‐induced ferroptosis. Mechanistically, in a TEAD‐dependent manner, YAP/TAZ induce the expression of SLC7A11, a key transporter maintaining intracellular glutathione homeostasis, thus enabling HCC cells to overcome Sorafenib‐induced ferroptosis. At the same time, YAP/TAZ sustain the protein stability, nuclear localization, and transcriptional activity of ATF4 which in turn cooperates to induce SLC7A11 expression. Our study uncovers a critical role of YAP/TAZ in the repression of ferroptosis and thus in the establishment of Sorafenib resistance in HCC, highlighting YAP/TAZ‐based rewiring strategies as potential approaches to overcome HCC therapy resistance.

## Graphical abstract

Resistance to therapy occurs in most liver cancer patients treated with Sorafenib, and patients succumb to the disease. A synthetic lethal screen identified a regulatory circuit, which prevents ferroptosis and promotes cancer cell survival, thus promoting resistance to Sorafenib.

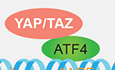

**The paper explained**

**Problem**

While treatment of liver cancer patients with Sorafenib, the current treatment of choice for advanced hepatocellular carcinoma, induces in most cases initial beneficial effects, resistance to Sorafenib therapy eventually occurs, tumors relapse, and patients succumb to the disease.

**Results**

We investigated the molecular mechanism underlying the development and maintenance of resistance to Sorafenib therapy in liver cancer cells. We found that the transcriptional regulators YAP/TAZ and ATF4 cooperatively induce the expression of genes required for antioxidant pathways, which are critical to prevent cancer cell death by ferroptosis. These pathways are also upregulated in tumors of Sorafenib‐resistant liver cancer patients. Proof‐of‐concept experiments with cultured liver cancer cells and in liver cancer mouse models revealed that inhibition of these pathways prevents the development of resistance to Sorafenib therapy.

**Impact**

These results suggest the possibility to re‐sensitize therapy‐resistant liver cancers to Sorafenib treatment by pharmacologically repressing the antioxidant pathways regulated by YAP/TAZ and ATF4.

## Introduction

Liver cancer is the second leading cause of cancer‐related mortality in patients. Hepatocellular carcinoma (HCC) represents about 90% of all cases of primary liver cancer (Llovet *et al*, 2008, 2016). Although cancer therapies have substantially improved clinical outcome (Kudo *et al*, 2018; Lee *et al*, 2020), patients invariably experience cancer relapse. Thus, delineating the mechanisms underlying evasive resistance of HCC is an unmet medical need and may add to a general understanding of therapy resistance in other cancer types as well.

Ferroptosis is an emerging type of cell death induced by metal iron and reactive oxygen species (ROS) and driven by lipid peroxidation (Dixon *et al*, 2012; Jiang *et al*, 2021). Among the core regulatory components, the cystine‐glutamate antiporter known as system Xc‐ or xCT (SLC7A11, encoded by the gene *SLC7A11*) imports cystine for the *de novo* synthesis of the important antioxidant peptide glutathione (GSH). GSH, among many functions, is also used as a substrate of phospholipid‐hydroxyperoxide‐glutathione‐peroxidase (GPX4) to catalyze the detoxification of phospholipid hydroperoxides (Lachaier *et al*, 2014). Hence, ferroptosis can be potently induced by cysteine deprivation and GPX4 inhibition. Small pharmacological inhibitors, including the GPX4 inhibitor RSL3, and Erastin and Sorafenib as direct inhibitors of xCT‐mediated import function, are widely used for the induction of ferroptosis (Dixon *et al*, 2012; Lachaier *et al*, 2014).

YAP/TAZ are well‐characterized transcriptional effectors of Hippo signaling involved in a variety of physio‐pathological processes, including tumorigenesis and tissue regeneration (Pan, 2010; Harvey *et al*, 2013). Previous studies have suggested the Hippo‐YAP/TAZ pathway is a key driver of ferroptosis in epithelial tumors (Wu *et al*, 2019; Yang *et al*, 2019). Here, we aimed at dissecting the molecular drivers of Sorafenib resistance in HCC and identified YAP/TAZ as negative regulators of ferroptosis. In a TEAD‐ and ATF4‐dependent manner, YAP/TAZ induce the expression of SLC7A11, thus assisting cells in overcoming Sorafenib‐induced ferroptosis. Moreover, the data suggest YAP/TAZ as key chaperones in stabilizing ATF4 protein and sustaining its nuclear transcriptional activity. Our study highlights YAP/TAZ as novel repressors of ferroptosis and, thus, as attractive therapeutic targets to overcome therapy resistance.

## Results

### Identification of YAP/TAZ as key drivers of Sorafenib resistance

To identify the molecular mechanisms underlying therapy resistance in HCC, we had previously established Sorafenib‐resistant HCC cell lines, called Huh7‐IR and Huh7‐CR (IC50 of 10.7 and 10.8 µM, respectively) and Hep3B‐IR and Hep3B‐CR (IC50 of 7.2 and 8.3 µM, respectively), as compared to their Sorafenib‐sensitive parental cell lines Huh7 and Hep3B (IC50 of 1.7 and 3.0 µM, respectively) (Appendix Fig S1A–C) (Tang *et al*, 2019; Gao *et al*, 2021). Global transcriptomic analysis between the Sorafenib‐sensitive parental cells and their Sorafenib‐resistant derivatives revealed that, in addition to changes in various signaling pathways, cellular metabolism pathways had dynamically shifted in HCC cells upon the establishment of Sorafenib resistance, including amino acid metabolism (Appendix Fig S1D; Dataset [Link], [Link]).

**Figure 1:**
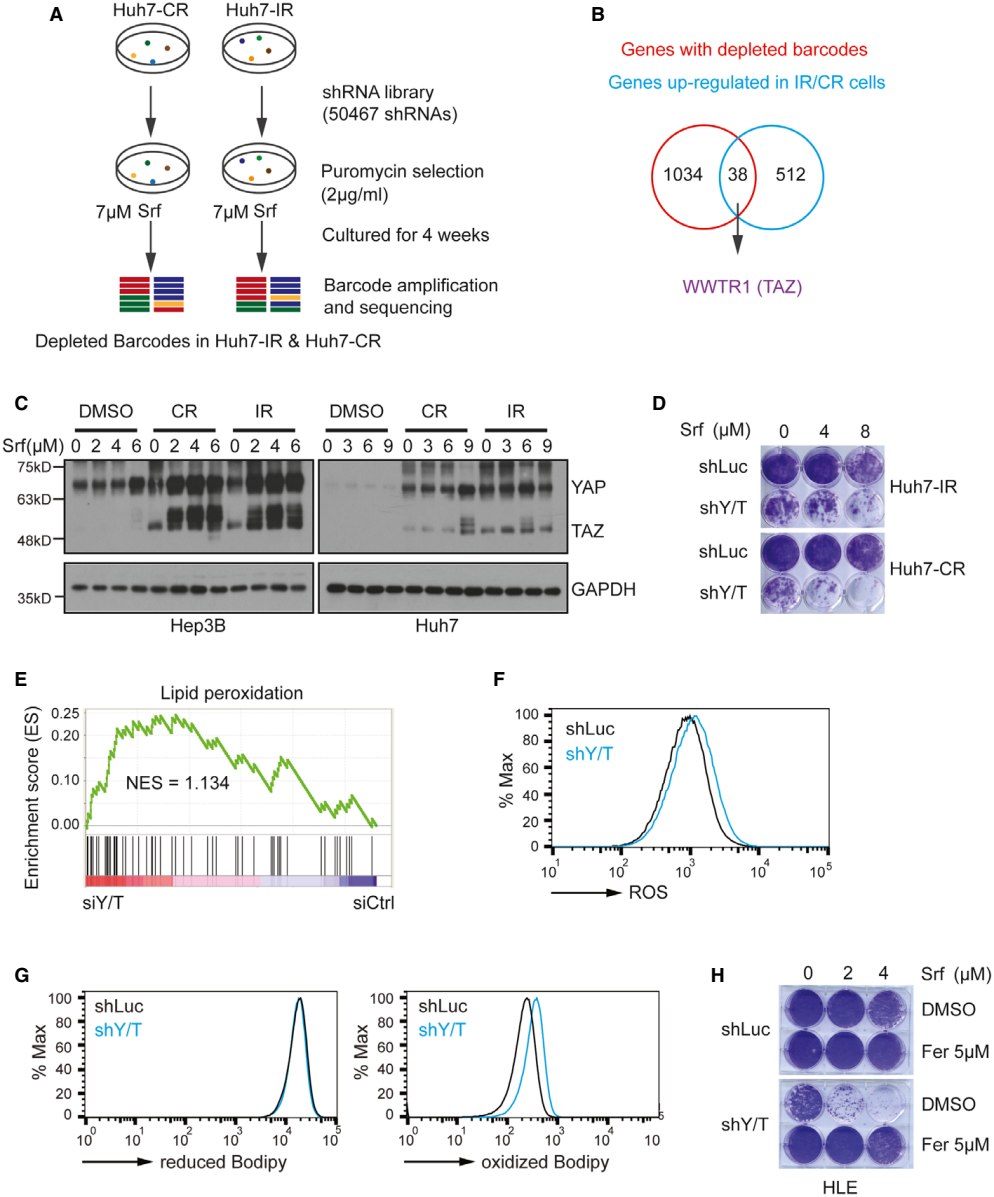
YAP/TAZ are key drivers of Sorafenib resistance by inhibiting ferroptosis Scheme of the shRNA‐mediated synthetic lethal screen. Huh7‐IR and CR cells were infected with lentiviral vectors (MOI = 0.5) expressing the shRNA library (human genome‐wide pooled lentiviral shRNA library module 1, vector: pRSI16cb, Cellecta) and cultured with 2 μg/ml puromycin for the selection of shRNA‐expressing cells plus 7 μM Sorafenib (Srf). After 4 weeks of culture, genomic DNA was extracted, and shRNA barcodes were amplified for next‐generation sequencing to uncover the critical genes for Sorafenib resistance.Combinatorial analysis of genes differentially expressed between Sorafenib‐sensitive and resistant cells and genes with depleted barcodes in the synthetic lethal screen in Huh7‐IR and CR cells. Thirty‐eight common genes were identified, among which was *WWTR1* coding for TAZ.Huh7‐parental, IR and CR, and Hep3B‐parental, and IR and CR cells were treated with different concentrations of Sorafenib (0, 3, 6, 9 μM) for Huh7‐P/IR/CR and Sorafenib (0, 2, 4, 6 μM) for Hep3B‐P/IR/CR for 18 h before harvest. Protein levels of YAP and TAZ were determined by immunoblotting illustrating higher protein levels of YAP/TAZ in Sorafenib‐resistant cells. GAPDH served as loading control. Results represent three independent experiments.Colony formation assay showing that shRNA‐mediated depletion of YAP/TAZ leads to cell numbers in response to Sorafenib treatment. Huh7 IR and CR cells either expressing a control shRNA (shLuc, non‐targeting shRNA) or shRNA against both YAP and TAZ (shY/T) were treated with different concentrations of Sorafenib (0, 4, 8 μM) for 2 weeks and colonies were visualized by crystal violet staining. Results represent three independent experiments.Gene Set Enrichment Analysis (GSEA) of the genes differentially expressed between YAP/TAZ‐deficient (siY/T) and control siRNA (siCtrl) transfected HLE cells showed an enrichment for genes involved in the regulation of lipid peroxidation.Basal reactive oxygen (ROS) levels increased upon loss of YAP/TAZ. HLE‐shLuc and HLE‐shY/T cell lines were stained with CellROX™ Green Flow Cytometry Assay Kit, and ROS levels were measured by flow cytometry using a 488 nm laser. Results represent three independent experiments.Basal lipid peroxidation levels increased with the loss of function of YAP/TAZ. HLE‐shLuc and HLE‐shY/T cells were stained with C11‐BODIPY 581/591. Reduced‐Bodipy was measured by flow cytometry using a 488 nm laser, and oxidized‐Bodipy was measured with a 561 nm laser. A significant shift of oxidized‐Bodipy occurred upon depletion of YAP/TAZ. Results represent three independent experiments.Colony formation assay demonstrating that the ferroptosis inhibitor Ferrostatin‐1 (Fer) reversed Sorafenib‐induced cell death in YAP/TAZ‐deficient HCC cells. HLE‐shLuc and shY/T cells were treated with different concentrations of Sorafenib (0, 2, 4 μM) and either DMSO or Ferrostatin‐1 (Fer; 5 μM) for 2 weeks. Results represent three independent experiments. Source data are available online for this figure.

To functionally identify intrinsic drivers of Sorafenib resistance in HCC cells, we performed a genome‐wide shRNA‐mediated synthetic lethality screen in Sorafenib‐resistant HCC cells (Fig 1A). Notably, we turned our focus on the identification of factors and pathways involved in the establishment of adaptive resistance to Sorafenib, as opposed to the factors and pathways activated during an acute response to Sorafenib treatment. Huh7‐IR and Huh7‐CR cells were transfected with a shRNA library selected to target all genes known to play a role in cell signaling and covering each gene with at least 8 independent shRNA sequences. Cells were then cultured for 4 weeks under selection conditions in the presence of Sorafenib. Then, genomic DNA was extracted, and shRNA barcodes were amplified for next‐generation sequencing to identify shRNAs and their target genes which have been lost during long‐term culture, indicating that these genes may be critical for the maintenance of Sorafenib resistance. shRNAs which had dropped out in parental Huh7 cells upon acute treatment with Sorafenib were subtracted from the list, to only enrich for the genes critical for the maintenance of Sorafenib resistance, and a total of 1,072 genes were identified (Appendix Fig S1E; Dataset [Link], [Link]). We also reasoned that critical drivers of Sorafenib resistance were deregulated at a transcriptional level during the adaptive reprogramming. Thus, we overlaid the hits from the synthetic lethality screen with the list of genes differentially regulated during the establishment of therapy resistance. This analytical combination revealed a total of 38 significant hits, among which eight genes were retrieved by more than three independent shRNA sequences and their gene regulation was highly significant (Fig 1B; Dataset [Link], [Link]). Interestingly, among these eight top candidate genes was the Hippo pathway transducer *WWTR1*, also known as TAZ.

The mammalian Hippo pathway has been previously implicated in tumorigenesis and therapy response in liver cancers (Harvey *et al*, 2013), and YAP and TAZ are well‐established Hippo transducers sharing redundant functional read‐outs (Totaro *et al*, 2018). Thus, we next focused our analysis on examining the expression of YAP and TAZ in Sorafenib‐sensitive and resistant HCC cell lines. Indeed, we found high expression of YAP and TAZ in Sorafenib‐resistant cells as compared to their sensitive counterparts (Fig 1C). Importantly, shRNA‐mediated ablation of YAP and TAZ expression revealed that they were required to maintain the acquired resistance to Sorafenib, as determined by colony formation assays (Fig 1D).

Together, these data indicate that YAP and TAZ represent critical drivers of acquired resistance to Sorafenib in HCC cells.

### YAP/TAZ promote resistance by antagonizing Sorafenib‐induced ferroptosis

To investigate the molecular mechanisms underlying YAP/TAZ‐driven Sorafenib resistance, we analyzed the global transcriptomic changes upon loss of YAP and TAZ in intrinsically Sorafenib‐resistant HLE cells (IC50 = 3.9 µM; Appendix Fig S1A) by RNA sequencing. This cell line was not highly resistant to Sorafenib, yet expressed substantial levels of YAP and TAZ proteins as compared to Sorafenib‐sensitive cell lines (Appendix Fig S1F) and exhibited a much higher transfection rate as compared to the more resistant SNU cell lines. Interestingly, among the deregulated pathways, the genes involved in the regulation of lipid peroxidation, a hallmark of ferroptosis, were found to be significantly enriched (Fig 1E). Notably, Sorafenib is known to potently promote ferroptosis by blocking SLC7A11‐mediated cellular cystine import (Dixon *et al*, 2014). We thus sought to validate the role of YAP and TAZ in the regulation of Sorafenib‐induced lipid ROS. Indeed, siRNA‐mediated loss of YAP and TAZ in HLE cells resulted in upregulated basal levels of ROS in HLE cells (Fig 1F). Moreover, loss of function of YAP and TAZ resulted in increased lipid peroxidation even in the absence of any treatment (Fig 1G), and even more so under treatment with Sorafenib or H_2_O_2_ (Appendix Fig S1G). Notably, Ferrostatin‐1, a specific inhibitor of ferroptosis (Dixon *et al*, 2012), prevented YAP/TAZ deficiency‐induced lipid peroxidation (Appendix Fig S1G). Consistent with these notions, intracellular GSH levels were decreased upon downregulation of YAP and TAZ (Appendix Fig S1H).

Based on these observations, we hypothesized that YAP and TAZ promoted Sorafenib resistance by detoxifying Sorafenib‐induced ferroptosis. We thus assessed whether Ferrostatin‐1 can prevent the cell death induced by Sorafenib upon loss of YAP and TAZ. Indeed, Ferrostatin‐1 fully restored the viability of YAP/TAZ‐deficient cells in the presence of Sorafenib, as determined by colony formation assay (Fig 1H). We further asked whether YAP/TAZ were able to antagonize ferroptosis in a general manner, not only in Sorafenib‐induced ferroptosis. To this end, we treated HLE cells with Erastin, a well‐known inducer of ferroptosis (Dixon *et al*, 2012) and observed that Erastin induced ferroptosis in shLuc‐transfected cells, which was further enhanced upon shRNA‐mediated ablation of YAP and TAZ. In both conditions, cell death could be blocked by treatment with Ferrostatin‐1, indicating that cell death was due to ferroptosis (Appendix Fig S1I). To further elaborate on the role of YAP and TAZ in supporting cell viability upon Sorafenib treatment of Sorafenib‐resistant HCC cells, we further employed the Promega Celltiter Glo^TM^ assay which confirmed the conclusion that YAP/TAZ promotes Sorafenib resistance via blocking ferroptosis in YAP/TAZ^high^ cells (Appendix Fig S1J and K).

Treatment of Huh7 parental, IR, and CR cells with other inducers of ferroptosis, including Erastin, RSL3, FIN58, and FINO2 all resulted into reduced cell viability of Sorafenib‐sensitive Huh7‐parental cells as compared to the Sorafenib‐resistant Huh7‐IR and Huh7‐CR cells (Appendix Fig S2A), further indicating that Sorafenib‐resistant cells are also more resistant to ferroptosis induction.

To further demonstrate the specificity of a role of YAP/TAZ in ferroptotic cell death, yet to exclude apoptosis or necroptosis, we treated siCtrl and siYAP/TAZ‐transfected HLE cells with Sorafenib and with the ferroptosis inhibitor Ferrostatin‐1, with the pan‐caspase inhibitor Z‐VAD‐FMK to repress apoptosis or with the RIPK3 inhibitor GSK‐872 to suppress necroptosis. Interestingly, only Ferrostatin‐1 rescued cell viability, thus confirming a specific role of YAP/TAZ in regulating Sorafenib‐induced ferroptosis (Appendix Fig S2B).

In contrast, cell death induced by acute Sorafenib treatment of Sorafenib‐sensitive (parental) Huh7 and Hep3B cells could not be reversed by treatment with Ferrostatin‐1 (Appendix Fig S3A and B). Given that YAP/TAZ expression was significantly lower in Sorafenib‐sensitive Huh7 and Hep3B cells as compared to their resistant counterparts (Fig 1C), we also assessed whether the forced expression of YAP/TAZ could overcome cell death induced by Sorafenib treatment of Sorafenib‐sensitive cells. Indeed, the forced expression of a constitutively active version of YAP (YAP‐5SA) prevented Sorafenib‐induced cell death (Appendix Fig S3C and D) and increased GSH levels (Appendix Fig S3E). This increased resistance to Sorafenib observed in YAP‐5SA‐expressing Huh7 and Hep3B cells as compared to empty vector control‐transfected cells was maintained in the presence of Erastin, although the overall levels of cell viability were dramatically reduced by Erastin (Appendix Fig S3F). These results are consistent with a previous report by our laboratory demonstrating that the acute treatment of Sorafenib‐sensitive HCC cells with Sorafenib induces autophagy and apoptosis which is prevented by YAP/TAZ activities (Tang *et al*, 2019). Yet, these results also indicates that YAP/TAZ not only counteract ferroptosis but also apoptosis in the context of Sorafenib treatment.

Altogether, the results demonstrate that YAP and TAZ act as general inhibitors of ferroptosis and apoptosis, thereby promoting Sorafenib resistance.

### YAP/TAZ transcriptionally upregulate SLC7A11

**Figure 2:**
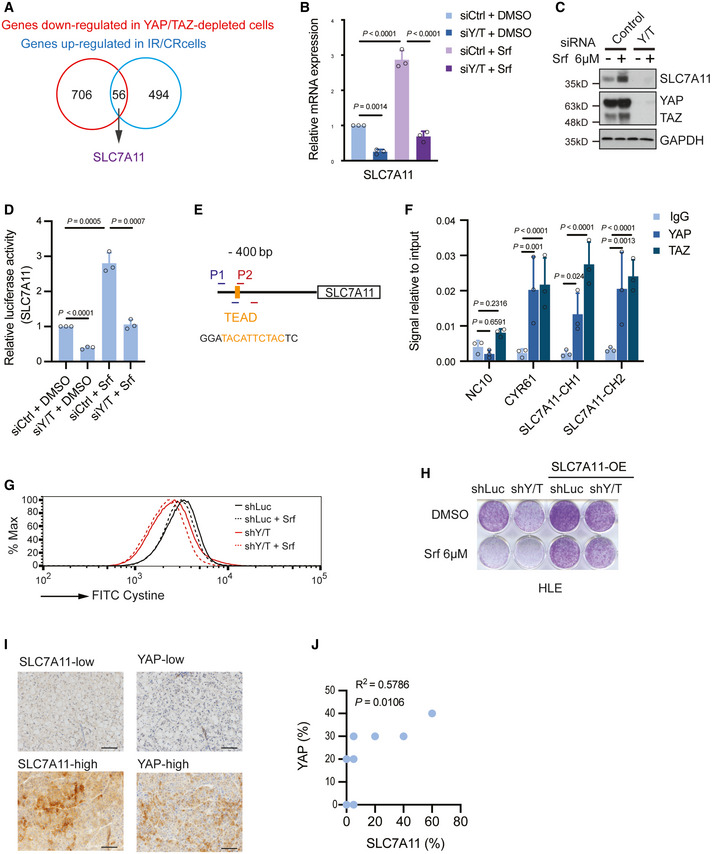
YAP/TAZ transcriptionally upregulate *SLC7A11* expression Combinatorial analysis of the genes upregulated in Sorafenib‐resistant cells and the genes downregulated upon YAP/TAZ depletion uncovered 56 common genes, among which was *SLC7A11*.Quantitative RT‐PCR analysis confirmed the dependency of *SLC7A11* gene expression on YAP/TAZ. HLE cells were transfected with control siRNA (siCtrl) or siRNA against YAP/TAZ (siY/T) and cultured with DMSO or 6 μM Sorafenib for 18 h. RNA was extracted and analyzed by quantitative RT‐PCR. Data are shown as mean ± standard deviation (SD). Statistical significance was calculated using one‐way ANOVA. Results represent three independent experiments.SLC7A11 protein levels were upregulated by the exposure to Sorafenib, yet downregulated by siRNA‐mediated depletion of YAP/TAZ. HLE cells were transfected with siCtrl or siY/T and cultured with DMSO or 6 μM Sorafenib for 18 h before harvest, followed by immunoblotting for YAP/TAZ and SLC7A1. GAPDH served as loading control. Results represent three independent experiments.siRNA‐mediated ablation of YAP/TAZ significantly reduced SLC7A11 promoter activity, as determined by SLC7A11‐promoter‐luciferase reporter assay. HLE cells were transfected with *SLC7A11*‐promoter *firefly* luciferase reporter construct and a constitutive‐active *Renilla* luciferase reporter construct (pRL‐CMV) and with siCtrl or siY/T. Relative luciferase activity was measured using the Dual‐Luciferase Reporter Assay Kit (Promega E1980). Data are shown as mean ± standard deviation (SD). Statistical significance was calculated using one‐way ANOVA. Results represent three independent experiments.A potential TEAD binding motif was predicted at – 400 bp within the promoter region of *SLC7A11*. PCR primers SLC7A11‐CH1 (P1) and SLC7A11‐CH2 (P2) were designed to examine the potential binding of transcription factors to the TEAD binding motif by chromatin immunoprecipitation (ChIP).Binding of YAP and TAZ to a DNA fragment containing the TEAD binding motif in the *SLC7A11* promoter. ChIP was performed on HLE cell lysate with antibodies against YAP and TAZ and rabbit IgG as control. DNA fragments were amplified using the primers specific for TEAD binding motif in the *SLC7A11* promoter region shown in (E). The non‐coding region NC10 served as negative control, and the bona fide TEAD target gene *CYR61* served as positive control. Data are shown as mean ± standard deviation (SD). Statistical significance was calculated using one‐way ANOVA. Results represent three independent experiments.Knockdown of YAP/TAZ impairs cystine uptake either with or without Sorafenib treatment, and cystine uptake decreased with the exposure to Sorafenib. HLE‐shLuc and HLE‐shY/T cells were cultured with DMSO or 6 μM Sorafenib for 18 h, cystine‐FITC was added to cells, and after incubation at 37°C for 30 min, intracellular Cystine‐FITC levels were measured by flow cytometry using a 488 nm laser. Results represent three independent experiments.Colony formation assay showing that stable overexpression of SLC7A11 reversed Sorafenib‐induced cell death in YAP/TAZ‐deficient HCC cells. HLE‐shLuc and HLE‐shY/T were or were not transfected to overexpress SLC7A11 (SLC7A11 OE) and treated with DMSO or 6 μM Sorafenib for 2 weeks as indicated. Results represent three independent experiments.Representative images of immunohistochemical staining of SLC7A11 and YAP proteins in HCC samples from patients. Scale bars, 50 μm.Quantification of the immunohistochemical stainings shown in (I) revealed a positive correlation of YAP and SLC7A11 expression (*N* = 10). Statistical significance was calculated using Pearson correlation analysis. Source data are available online for this figure.

To further investigate how the transcription factors YAP/TAZ restrict ferroptosis, we set out to identify their transcriptional target genes in the context of Sorafenib resistance of HCC cells. To this end, we overlaid the list of genes downregulated in their expression in YAP/TAZ‐depleted HLE cells and the list of genes upregulated in Sorafenib‐resistant cells, which led to the identification of 56 genes (Dataset [Link], [Link]). Of note, *SLC7A11*, coding for the cystine‐glutamate antiporter known to regulate ferroptosis, was among these genes (Fig 2A).

As a key regulator of lipid peroxidation and ferroptosis, we confirmed the functional importance of SLC7A11 for Sorafenib resistance in HCC cells. Loss of SLC7A11 resulted in an increase of intracellular ROS levels (Appendix Fig S4A) as well as of lipid peroxidation (Appendix Fig S4B). Moreover, cystine uptake was reduced by siRNA‐mediated depletion of SLC7A11 (Appendix Fig S4C) and, as a consequence, the levels of intracellular GSH were diminished (Appendix Fig S4D). Finally, the depletion of SLC7A11 resulted in increased cell death in a colony formation assay which was fully rescued by Ferrostatin‐1 (Appendix Fig S4E and F). These results confirm SLC7A11 as a key regulator of ferroptosis in response to Sorafenib.

We next explored the expression of SLC7A11 in HCC of patients. Of note, SLC7A11 mRNA was highly upregulated in primary HCC tumors. Further, immunohistochemical analysis of HCC tumor sections revealed that SLC7A11 was upregulated in HCC of patients as compared with adjacent liver parenchyma (Appendix Fig S4G). Statistical analysis using multi‐tissue arrays with non‐tumor and tumor tissues confirmed its high expression in HCC (Appendix Fig S4H). Interestingly, SLC7A11 protein significantly correlated with reduced cell differentiation of HCC samples, as classified by Edmondson grades III and IV (Appendix Fig S4I). Even more important, using the expression data retrieved from the TCGA liver dataset (Cancer Genome Atlas Research Network. Electronic address & Cancer Genome Atlas Research, 2017), we observed that *SLC7A11* expression predicted patient survival, with high expression of *SLC7A11* significantly correlating with poor clinical outcome (Appendix Fig S4J).

We next sought to validate whether YAP/TAZ directly affected *SLC7A11* gene expression. Indeed, knockdown of YAP and TAZ together resulted in a significant downregulation of SLC7A11 at both mRNA level and protein level (Appendix Fig S5A; Fig 2B and C). Moreover, overexpression of a constitutive‐active form of YAP induced an upregulation of *SLC7A11* (Appendix Fig S5B). The transcriptional activity of YAP/TAZ was best known to be regulated by cell–cell contact, and we thus further explored whether *SLC7A11* gene expression was also sensitive to changes in cell density. Indeed, the expression of *SLC7A11* was significantly downregulated with increasing cell density (Appendix Fig S5C).

To determine whether YAP/TAZ directly regulated *SLC7A11* gene expression, we first analyzed the transcriptional activity of a *SLC7A11*‐promoter‐luciferase reporter construct in dependence on YAP/TAZ activities. Indeed, we observed that the *SLC7A11* promoter was fully responsive to the absence or presence of YAP/TAZ (Fig 2D). Moreover, analysis of the *SLC7A11* promoter sequence identified a potential TEAD binding motif approximately 400 bps upstream of the transcriptional start site (Fig 2E). Chromatin immunoprecipitation with specific antibodies to YAP or TAZ followed by quantitative PCR (ChIP‐qPCR) demonstrated that YAP or TAZ bound to the DNA fragment containing the TEAD motif in the promoter of the *SLC7A11* gene (Fig 2F).

To functionally validate the critical role of YAP/TAZ‐mediated *SLC7A11* expression in Sorafenib resistance, we asked whether the cystine‐glutamate antiporter SLC7A11 acted downstream of YAP/TAZ in overcoming Sorafenib‐induced ferroptosis and whether it could replace YAP/TAZ activities. Interestingly, knockdown of YAP/TAZ resulted in impaired cystine uptake (Fig 2G). Moreover, the forced expression of SLC7A11 was able to prevent Sorafenib‐induced death in YAP/TAZ‐deficient HLE cells (Fig 2H; Appendix Fig S5D).

To assess the functional connection of YAP/TAZ and SLC7A11 expression in patients, we explored the correlation of YAP and SLC7A11 expression in HCC samples from patients. In fact, a significant positive correlation was observed between YAP and SLC7A11 protein levels in HCC patient samples (Fig 2I and J), suggesting YAP as one of the key regulators of *SLC7A11* gene expression during HCC development.

Together, the results show that *SLC7A11*, as a direct transcriptional target of YAP/TAZ, is upregulated in HCC and, as mRNA or protein, is a prognostic factor of HCC aggressiveness and of clinical outcome. Importantly, SLC7A11 acts epistatically downstream of YAP and TAZ to drive Sorafenib resistance in HCC.

### ATF4 regulates SLC7A11 in response to Sorafenib treatment

**Figure 3:**
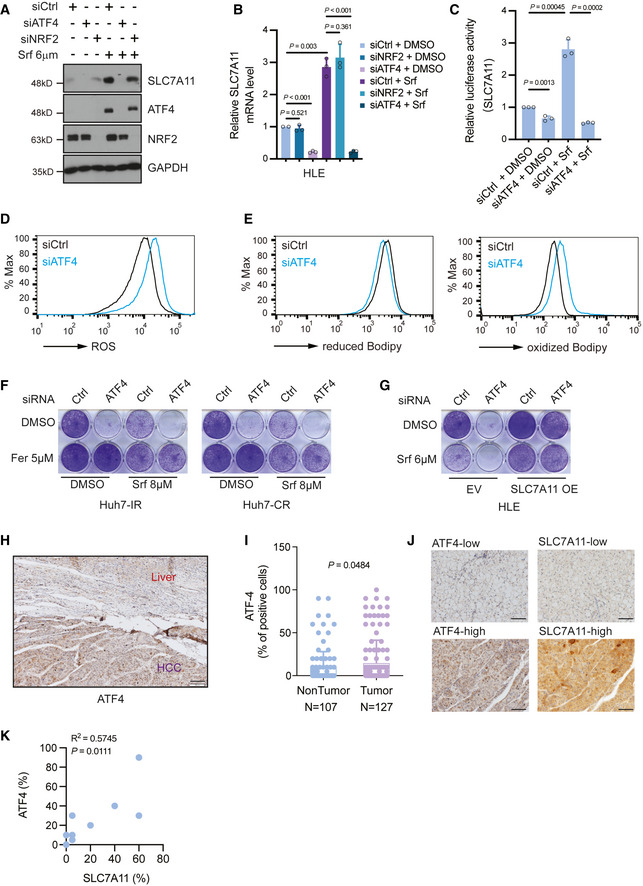
ATF4 regulates SLC7A11 in response to Sorafenib treatment SLC7A11 protein levels decreased upon siRNA‐mediated depletion of ATF4 but not of NRF2 either with or without Sorafenib treatment, and Sorafenib promoted the expression of ATF4. HLE cells were transfected with siCtrl, siATF4 or siNRF2 and treated with or without 6 μM Sorafenib for 18 h. The expression of SLC7A11, ATF4, and NRF2 was determined by immunoblotting. GAPDH served as loading control. Results represent three independent experiments.
*SLC7A11* mRNA levels were decreased upon ATF4 depletion but not NRF2 depletion. HLE cells were transfected with siCtrl, siATF4, or siNRF2 and cultured with DMSO or 6 μM Sorafenib for 18 h. Quantitative RT‐PCR was used to determine *SLC7A11* mRNA levels. Data are shown as mean ± standard deviation (SD). Statistical significance was calculated using one‐way ANOVA. Results represent three independent experiments.siRNA‐mediated ablation of ATF4 significantly reduced *SLC7A11* promoter activity, as determined by *SLC7A11*‐promoter‐luciferase reporter assay. HLE cells were transfected with *SLC7A11*‐promoter *firefly* luciferase reporter construct and a constitutive‐active *Renilla* luciferase reporter construct (pRL‐CMV) and with siCtrl or siATF4 and treated or not with 6 µM Sorafenib. Relative luciferase activity was measured using the Dual‐Luciferase Reporter Assay Kit (Promega E1980). Data are shown as mean ± standard deviation (SD). Statistical significance was calculated using one‐way ANOVA. Results represent three independent experiments.Basal reactive oxygen (ROS) levels increased upon loss of ATF4. HLE cells were transfected with siCtrl or siATF4 and cultured for 36 h were stained with CellROX™ Green Flow Cytometry Assay Kit, ROS levels were measured by flow cytometry using a 488 nm laser. Results represent three independent experiments.Basal lipid peroxidation levels increased with the loss of ATF4. HLE cells were transfected with siCtrl or siATF4 and cultured for 36 h were stained with C11‐BODIPY 581/591. Reduced‐Bodipy was measured by flow cytometry using a 488 nm laser, and oxidized‐Bodipy was measured with a 561 nm laser. A significant shift of oxidized‐Bodipy occurred upon depletion of ATF4. Results represent three independent experiments.Colony formation assay demonstrating that the ferroptosis inhibitor Ferrostatin‐1 (Fer) reversed Sorafenib‐induced cell death in ATF4‐deficient HCC cells. HLE cells transfected with siCtrl or siATF4 were treated with Sorafenib (8 μM) or DMSO plus either DMSO or Ferrostatin‐1 (Fer; 5 μM) for 2 weeks. Results represent three independent experiments.The forced expression of SLC7A11 rescued from cell death induced by ATF4 ablation. HLE cells were transfected with a construct coding for SLC7A11 (SLC7A11 OE) or empty vector (EV) and with siCtrl or siATF4 every other day. Cells were then treated with either DMSO or 6 μM Sorafenib for 2 weeks. Colony formation was visualized by crystal violet staining. Results represent three independent experiments.Immunohistochemical staining of ATF4 in HCC and adjacent non‐neoplastic areas from patients. Tumor tissues showed a higher expression of ATF4. Scale bar, 100 μm.Quantification of ATF4‐positive cells in tumor and non‐tumor samples showed that HCC tumors present higher ATF4 levels. Data are shown as mean ± standard deviation (SD). Statistical significance was calculated using unpaired *t*‐test.Representative images of immunohistochemical staining of ATF4 and SLC7A11 proteins in HCC samples from patients. Scale bars, 50 μm.Quantification of the immunohistochemical stainings in (J) showed a positive correlation of ATF4 and SLC7A11 expression (*N* = 10). Statistical significance was calculated using Pearson correlation analysis. Source data are available online for this figure.

Our study revealed YAP/TAZ as novel regulators of *SLC7A11* gene expression. Interestingly, Sorafenib can potently induce *SLC7A11* expression, while it does not boost YAP/TAZ activity (Tang *et al*, 2019). This prompted us to investigate alternative key factors driving *SLC7A11* expression in response to Sorafenib treatment. It is well known that stress‐induced *SLC7A11* expression can be controlled by the ROS sensor NRF2 (Sun *et al*, 2016) and the stress regulator ATF4 (Chen *et al*, 2017). Therefore, we tested the contribution of these two transcription factors by siRNA‐mediated loss‐of‐function studies. Surprisingly, we observed that ATF4, rather than NRF2, was a predominant driver of SLC7A11 expression in Sorafenib‐resistant cell lines HLE cells (Fig 3A and B) and Huh7‐IR and Huh7‐CR cells (Appendix Fig S6A and B). Interestingly, ATF4 itself was upregulated in response to Sorafenib (Fig 3A) and highly expressed in Sorafenib Huh7‐IR and Huh7‐CR cells as compared to their parental Sorafenib‐sensitive cells (Appendix Fig S6C). This upregulated expression of ATF4 is likely due to Sorafenib‐induced ER stress (Kim *et al*, 2011; Dixon *et al*, 2014). Sorafenib‐induced ATF4 activity in return increased *SLC7A11* promoter activity in a luciferase reporter assay, suggesting that ATF4 directly regulated *SLC7A11* gene expression in response to Sorafenib (Fig 3C).

We next investigated the functional consequences of increased ATF4 activity on HCC cell survival and found that loss of ATF4 resulted in increased ROS levels as well as lipid peroxidation (Fig 3D and E). Moreover, cystine uptake and intracellular GSH level were downregulated upon ATF4 depletion (Appendix Fig S6D and E). Consequently, loss of ATF4 by two different siRNA sequences resulted in decreased cell viability in a colony formation assay and in cell viability assays, which was fully blocked by the ferroptosis inhibitor Ferrostatin‐1 (Fig 3F; Appendix Fig S6F–H). In addition, the forced expression of SLC7A11 in ATF4‐deficient cells also restored cell viability (Fig 3G), suggesting that ATF4 was a key transcriptional factor for the repression of ferroptosis by inducing *SLC7A11* gene expression.

We further explored the expression of ATF4 in HCC patient samples and found that ATF4 was upregulated in tumor tissues in comparison to adjacent liver parenchyma (Fig 3H). Quantification of ATF4 levels in a multi‐tissue arrays of patient samples further revealed that ATF4 was highly expressed in HCC tumors as compared to non‐tumor parenchyma (Fig 3I). Finally, the significant correlation of ATF4 and SLC7A11 protein levels suggested that these two proteins were co‐expressed in HCC of patients (Fig 3J and K).

Together, our study reveals that ATF4 is a key regulator of SLC7A11 expression and, thus, prevents Sorafenib‐induced ferroptosis in HCC.

### YAP/TAZ stabilize and direct ATF4 into the nucleus

Our study thus far identified YAP/TAZ and ATF4 as joint regulators of *SLC7A11* gene expression. The fact that ATF4 was upregulated by Sorafenib and simultaneously regulated *SCL7A11* expression motivated us to investigate a potential crosstalk of YAP/TAZ with ATF4 in the regulation of *SLC7A11* expression.

**Figure 4:**
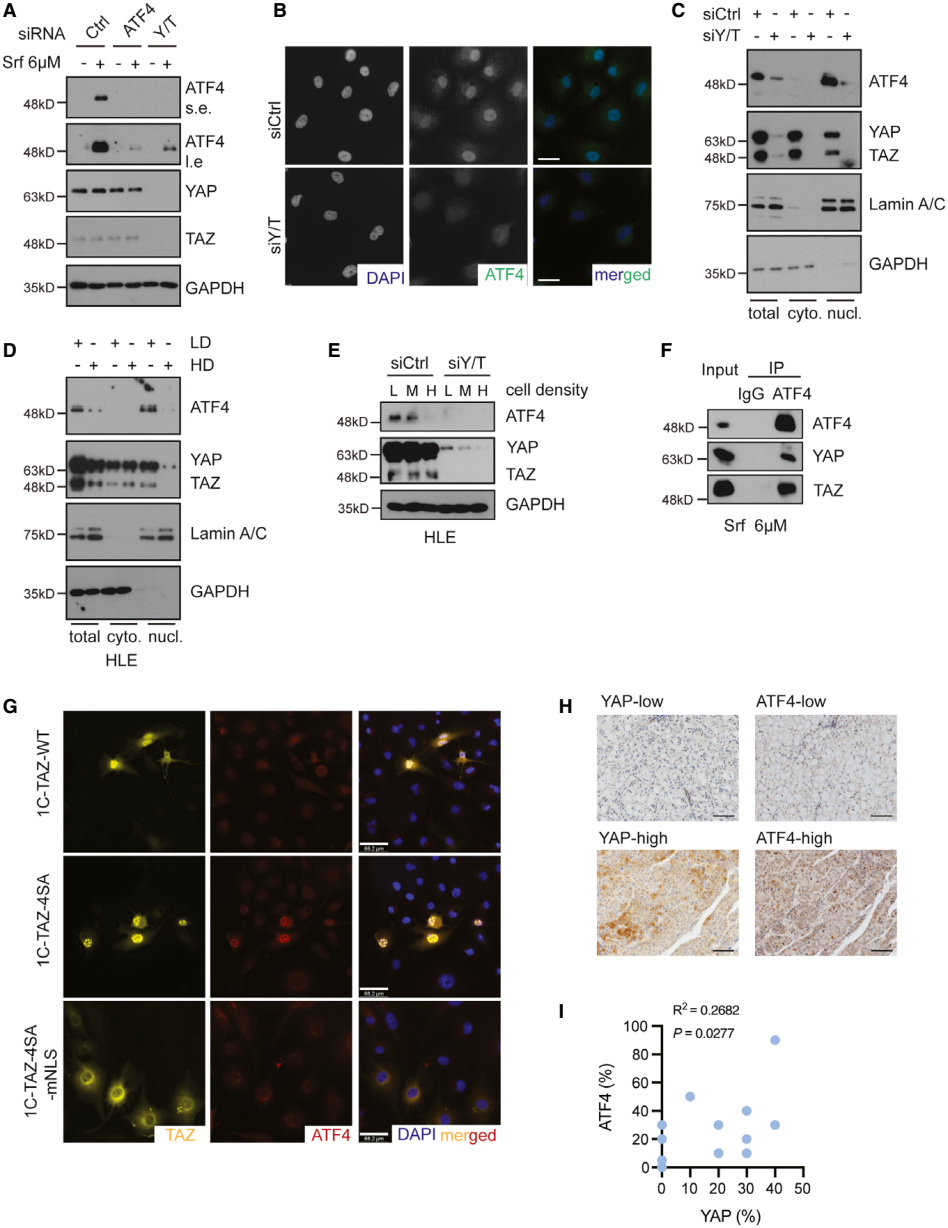
YAP/TAZ stabilize ATF4 protein and direct it into the nucleus YAP/TAZ deficiency repressed Sorafenib‐induced expression of ATF4, whereas ATF4 depletion had no effect on YAP/TAZ protein levels. HLE cells were transfected with siCtrl or siATF4 or siY/T and treated with 6 μM Sorafenib or not for 18 h. ATF4 and YAP/TAZ protein levels were analyzed by immunoblotting. GAPDH served as loading control. Results represent three independent experiments.YAP/TAZ deficiency induced a reduction of ATF4 protein level and nuclear localization. HLE cells were transfected with siCtrl or siY/T, and ATF4 and YAP/TAZ were visualized by immunofluorescence microscopy. DAPI visualized nuclei. Scale bars, 50 μm. Results represent three independent experiments.Cellular fractionation revealed that YAP/TAZ deficiency reduced ATF4 nuclear localization. HLE cells were transfected with siCtrl or siY/T, and cell lysates were separated into cytoplasmic and nuclear fractions and analyzed by immunoblotting for ATF4 and YAP/TAZ. GAPDH served as loading control of total lysate and cytoplasmic fractions, Lamin A/C served as loading control for nuclear proteins. Results represent three independent experiments.Cellular fractionation revealed that high cell density and, with it, lower YAP/TAZ activity reduced the levels of nuclear ATF4. HLE cells were seeded at different cell numbers, and cell lysates of high cell density cells (HD) and low cell density (LD) were fractionated into cytoplasmic and nuclear fractions and analyzed for ATF4 and YAP/TAZ by immunoblotting. GAPDH served as loading control for total lysate and cytoplasmic proteins, Lamin A/C served as loading control for nuclear proteins. Results represent three independent experiments.Increasing cell density declined YAP/TAZ stability and led to a reduction of ATF4. HLE cells were seeded to reach low (L), medium (M), and high (H) cell densities and transfected with siCtrl or siY/T. Protein levels of ATF4 and YAP/TAZ were determined by immunoblotting blotting. GAPDH served as loading control. Results represent three independent experiments.YAP/TAZ physically interacted with ATF4. HLE cells were cultured with 6 μM Sorafenib for 18 h and ATF4 was immunoprecipitated and precipitated YAP, TAZ, and ATF4 were visualized by immunoblotting. Unrelated IgG was used as control for immunoprecipitation. Input represents cell lysates directly analyzed by immunoblotting. Results represent three independent experiments.YAP/TAZ directed the nuclear localization of ATF4. HLE cells were transfected with plasmid constructs coding for wild‐type TAZ (1C‐TAZ‐WT), constitutive‐active TAZ (1C‐TAZ‐ 4SA), or constitutive‐active TAZ lacking a nuclear localization signal (NLS) (1C‐TAZ‐mNLS) and analyzed for TAZ and ATF4 expression and localization by immunofluorescence microscopy. DAPI visualized nuclei. Scale bars, 66.2 μm. Results represent three independent experiments.Representative images of immunohistochemical staining of ATF4 and YAP proteins in HCC samples from patients. Scale bars, 50 μm.Quantification of the immunohistochemical staining in (J) showed a positive correlation of ATF4 and YAP expression (*N* = 18). Statistical significance was calculated using Pearson correlation analysis. Source data are available online for this figure.

We first assessed whether YAP/TAZ and ATF4 affected each other’s expression levels in response to Sorafenib. Indeed, YAP/TAZ deficiency resulted in a marked downregulation of Sorafenib‐induced ATF4 expression at the protein level (Fig 4A). Subcellular localization by immunofluorescence microscopy analysis as well as cellular fractionation and immunoblotting revealed that loss of YAP/TAZ resulted into a reduction of nuclear ATF4 in HCC cells (Fig 4B–D). Moreover, high cell density culture resulted in the expected deactivation of YAP/TAZ, but also in decreased ATF4 expression in HCC cells (Fig 4E).

To further explore how YAP/TAZ regulates ATF4 protein levels, we next asked whether YAP/TAZ affected ATF4 protein stability and/or whether YAP/TAZ directly induced ATF4 transcriptional activation. Protein levels can be regulated by proteasome‐mediated degradation or auto‐lysosomal‐driven turnover. Thus, we treated HCC cells with the proteasome inhibitor MG132 and we found that it efficiently blocked the downregulation of ATF4 induced by the siRNA‐mediated depletion of YAP/TAZ (Appendix Fig S7A). In contrast, treatment with chloroquine (CQ) as an inhibitor of lysosomal degradation had no effect on ATF4 protein levels. Proteasomal degradation is generally preceded by protein ubiquitination. We thus analyzed the influence of YAP/TAZ expression on the ubiquitination of ATF4. Indeed, the loss of YAP/TAZ resulted in increased ubiquitination of ATF4 (Appendix Fig S7B). These results suggested that YAP/TAZ promoted ATF4 function by restricting ATF4 poly‐ubiquitination and thus stabilizing ATF4.

It is well known that the shuttling of YAP/TAZ between cytoplasm and nucleus enables them to act as core chaperones and to facilitate the subcellular translocation of specific factors, such as β‐catenin (Heallen *et al*, 2011). Therefore, we asked whether YAP/TAZ directed ATF4 to the nucleus. Analysis of a direct protein–protein interaction by co‐immunoprecipitation revealed that ATF4 indeed physically bound to YAP and TAZ (Fig 4F). To study the active role of YAP/TAZ in translocating ATF4 to the nucleus, we transfected HLE HCC cells with plasmid constructs coding for wild‐type TAZ, constitute‐active TAZ, or constitutive‐active TAZ lacking the nuclear localization signal (NLS), and analyzed ATF4 subcellular localization by immunofluorescence microscopy. Notably, wild‐type TAZ and in particular constitutive‐active TAZ increased nuclear ATF4, while constitutive‐active TAZ lacking a NLS failed to translocate ATF4 into the nucleus (Fig 4G). Since TEADs have been shown to promote the nuclear import of YAP/TAZ (Diepenbruck *et al*, 2014), we also assessed whether TEADs indirectly affected ATF4 nuclear localization. Indeed, siRNA‐mediated ablation of all TEADs resulted in a loss of nuclear ATF4 and also in a substantial reduction of ATF4 protein levels (Appendix Fig S7C–E).

We further explored the correlation of YAP and ATF4 expression in HCC of patients. Intriguingly, a significant positive correlation was observed in HCC patient samples, suggesting YAP as a key regulator of ATF4 protein levels in HCC (Fig 4H and I).

Together, the data indicate that YAP/TAZ interact with ATF4 and together with TEADs promote its nuclear import to prevent its ubiquitylation and proteasomal degradation in the cytoplasm.

### YAP/TAZ and ATF4 collaboratively regulate SLC7A11 expression

**Figure 5:**
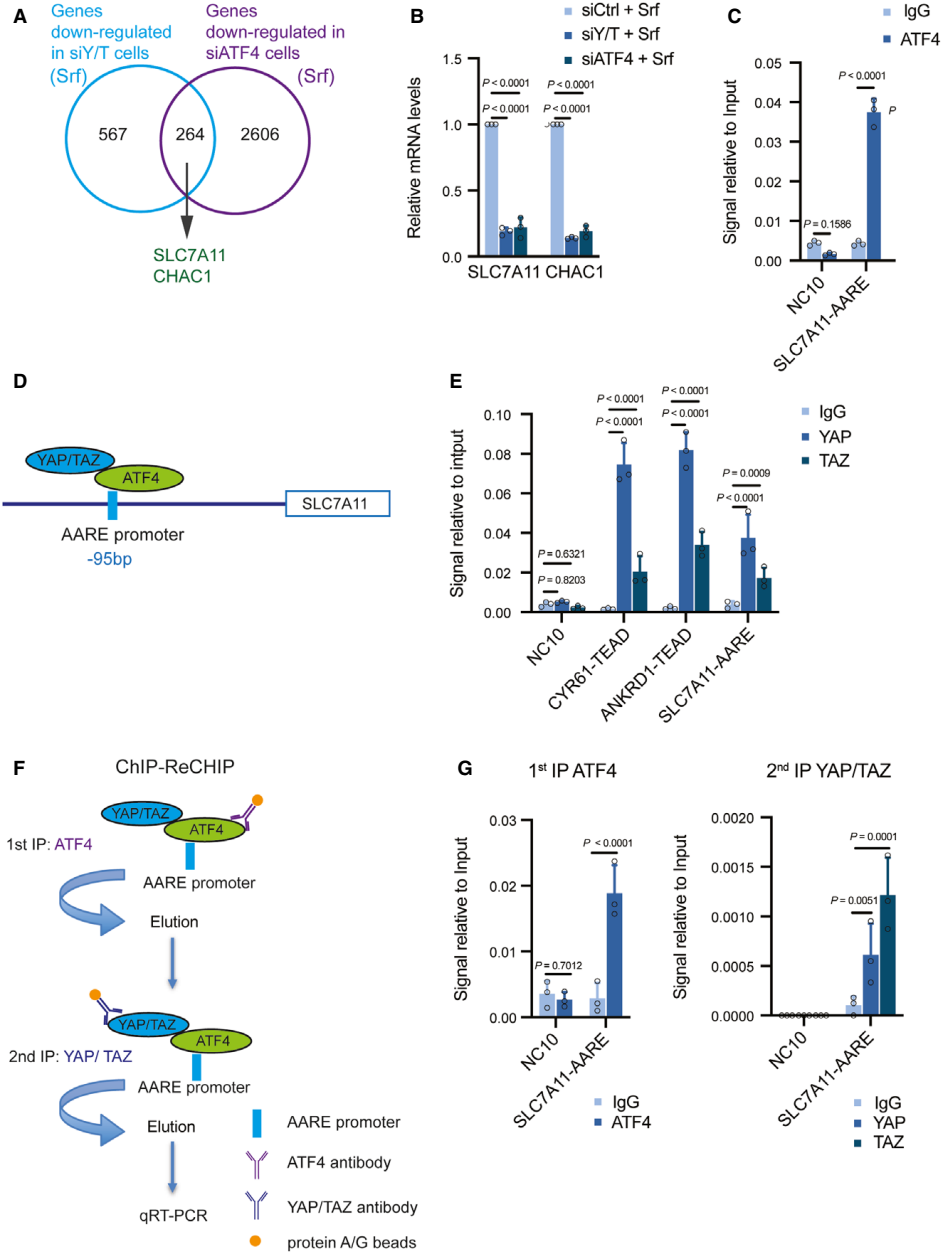
YAP/TAZ‐ATF4 collaboratively regulate *SLC7A11* expression A total of 264 common genes were identified downregulated by both deficiency for YAP/TAZ and deficiency for ATF4, among which were known ATF4 targets, such as *SLC7A11* and *CHAC1*. HLE cells were transfected with siCtrl, siYAP/TAZ, or siATF4 and cultured with 6 μM Sorafenib for 18 h and RNA was subjected to next‐generation RNA sequencing. Venn diagram analysis was conducted using VENNY 2.1.0.Quantitative RT‐PCR analysis verified that depletion of YAP/TAZ or ATF4 declined the expression levels of *SLC7A11* and *CHAC1*. HLE cells were transfected with siCtrl, siY/T, or siATF4 and cultured with 6 μM Sorafenib for 18 h. Quantitative RT‐PCR was conducted to determine *SLC7A11* and *CHAC1* mRNA levels. Data are shown as mean ± standard deviation (SD). Statistical significance was calculated using two‐way ANOVA. Results represent 3 independent experiments.Binding of ATF4 to DNA fragments containing the AARE binding motif in the promoters of *SLC7A11* and *ATF*3. ChIP was performed on HLE cell lysate with antibodies against ATF4 and rabbit IgG as control. DNA fragments were amplified using the primers specific for AARE binding motif in the *SLC7A11* promoter region. The non‐coding region NC10 served as negative control. Data are shown as mean ± standard deviation (SD). Statistical significance was calculated using one‐way ANOVA. Results represent three independent experiments.Schematic representation of the ATF4 binding motif AARE located at – 95 bp of the *SLC7A11* promoter region. YAP/TAZ were predicted to interact with ATF4 binding to the AARE binding motif in the promoter of *SLC7A11*.Binding of YAP and TAZ to DNA fragments containing the AARE binding motif in the *SLC7A11* promoter. ChIP was performed on HLE cell lysate with antibodies against YAP and TAZ and rabbit IgG as control. DNA fragments were amplified using the primers specific for AARE binding motif in the *SLC7A11* promoter region. The non‐coding region NC10 served as negative control, and the bona fide TEAD target genes *CYR61* and *ANKRD1* served as positive controls. Data are shown as mean ± standard deviation (SD). Statistical significance was calculated using one‐way ANOVA. Results represent three independent experiments.Schematic representation of the ChIP‐ReChIP strategy to test whether YAP/TAZ bind to the AARE binding motif within the *SLC7A11* promoter via binding to ATF4.YAP/TAZ bind to DNA fragments containing the AARE binding motif within the *SLC7A11* promoter via ATF4. HLE cells were cultured with 6 μM Sorafenib for 18 h before harvest. In a 1^st^ round ChIP ATF4 was immunoprecipitated with antibody against ATF4, rabbit IgG was used as control. DNA‐protein immunocomplexes were eluted and in a 2^nd^ round ChIP antibody against YAP/TAZ was used to precipitate DNA fragments which were then amplified and analyzed by quantitative PCR for the AARE motif in the *SLC7A11* promoter. NC10 served as negative PCR control. Data are shown as mean ± standard deviation (SD). Statistical significance was calculated using one‐way ANOVA. Results represent three independent experiments. Source data are available online for this figure.

Since YAP/TAZ appeared to regulate ATF4 nuclear activity and together they induced *SLC7A11* expression, we next asked whether and how YAP/TAZ and ATF4 act globally in response to Sorafenib. We employed RNA sequencing to compare the expression of genes affected by siRNA‐mediated ablation of either YAP/TAZ or ATF4 in the presence of Sorafenib. A total of 262 genes were found co‐regulated by YAP/TAZ and ATF4, among which were well‐known ATF4 targets, such as *CHAC1* and *SLC7A11* (Fig 5A; Dataset [Link], [Link]). Quantitative RT‐PCR further confirmed that these genes were co‐regulated by YAP/TAZ and ATF4 (Fig 5B). We next assessed whether ATF4 directly regulated their expression via canonical amino acid responsive element (AARE) motifs. ChIP‐qPCR analysis revealed that ATF4 indeed bound to DNA fragments containing the AARE regions of the *ATF3* and *SLC7A11* gene promoters (Fig 5C).

Our previous results demonstrated that YAP/TAZ regulates *SLC7A11* expression via a TEAD binding motif on its gene promoter. Given that a fraction of YAP/TAZ targets was found co‐regulated by ATF4, we further investigated an alternative regulation of *SLC7A11* by YAP/TAZ, such as via the AARE motif in the *SLC7A11* promoter. Indeed, ChIP‐qPCR analysis revealed that YAP/TAZ was also able to bind to a DNA fragment containing the AARE motif in the *SLC7A11* gene promoter (Fig 5D and E). Given that YAP/TAZ physically interacted with ATF4 and that YAP/TAZ and ATF4 bound to the AARE motif in the *SLC7A11* gene promoter, ATF4 might mediate the indirect binding of YAP/TAZ to the AARE motif. To test this, a ChIP‐ReChIP assay was performed with a 1^st^ round of anti‐ATF4 ChIP followed by a 2^nd^ round of anti‐YAP/TAZ ChIP (Fig 5F). Indeed, we found that the AARE motif in the *SLC7A11* gene promoter was significantly enriched by YAP/TAZ in the 2^nd^ round ChIP (Fig 5G), indicating that YAP/TAZ bound to the DNA fragment containing the AARE motif in the *SLC7A11* gene promoter via binding to ATF4. Together, the results suggest that YAP/TAZ and ATF4 collaboratively regulate *SLC7A11* expression in HCC cells.

We next analyzed the effect of ATF4 overexpression on the expression of SLC7A11 and CHAC1. Interestingly, while overexpression of ATF4 alone significantly induced the expression of SCL7A11 and CHAC1, the concomitant loss of YAP/TAZ reduced the ATF4‐mediated induction of SLC7A11 and CHAC1 expression (Appendix Fig S8A).

To assess the general importance of the cooperative action of YAP/TAZ and ATF4 in overcoming ferroptosis in various cancer cell types, we have further addressed the roles of YAP/TAZ and ATF4 in HT1080 human fibrosarcoma cells in response to a variety of ferroptotic stimuli. Interestingly, while YAP/TAZ and ATF4 loss of function resulted in higher rates of ferroptosis upon RSL3, FIN56, and FINO2 treatments (Appendix Fig S8B and C). More importantly, treatment with Ferrostatin‐1 rescued the ferroptotic stimuli‐induced cell death, confirming cell death via ferroptosis. Interestingly, we observed that there was no difference in the rate of ferroptosis in HT1080 cell upon YAP/TAZ or ATF4 deficiency and Erastin treatment. In fact, a recent report by the Conrad laboratory demonstrated that HT1080 cells fails to response to the SLC7A11 (xCT) blocker Sorafenib (Zheng *et al*, 2021). While both Sorafenib and Erastin target xCT to induce ferroptosis, how these two compounds exerted their functions so divergently in comparison with other ferroptotic inducers in the context of YAP/TAZ and ATF4 was unknown; it may be due to the inhibition of other targets of Sorafenib and/or Erastin.

To further assess a general role of YAP/TAZ in restricting ferroptosis, we analyzed Sorafenib and RSL‐3‐induced ferroptosis in MDA‐MB‐231 human breast cancer cells, a cell line widely used to study breast cancer metastasis. In line with our conclusion, overexpressed YAP can induce ferroptosis resistance in MDA‐MB‐231 cells as well (Appendix Fig S8D and E).

### Targeting ferroptosis overcomes HCC Sorafenib resistance *in vivo*

Our results suggested that YAP/TAZ and ATF4 promoted Sorafenib resistance by upregulating *SLC7A11* gene expression and thus antagonizing ferroptosis. We next investigated the therapeutic effect of targeting YAP/TAZ in combination with Sorafenib on tumor outgrowth of HCC cells *in vivo*. However, neither the human Sorafenib‐resistant HCC cell lines tested nor the Sorafenib‐resistant Huh7 and Hep3B‐IR and CR cells showed efficient tumor outgrowth upon transplantation into immunodeficient NSG mice, with the exception of SNU398 cells which were thus used for further experimentation. SNU398 cells also exhibited increased cell death induced by Sorafenib in YAP/TAZ or in ATF4‐depleted cells *in vitro*, which could be reversed by treatment with the ferroptosis inhibitor Ferrostatin‐1 but not with the apoptosis inhibitor Z‐VAD‐FMK or the necroptosis inhibitor GSK872 (Appendix Fig S9A and B).

**Figure 6:**
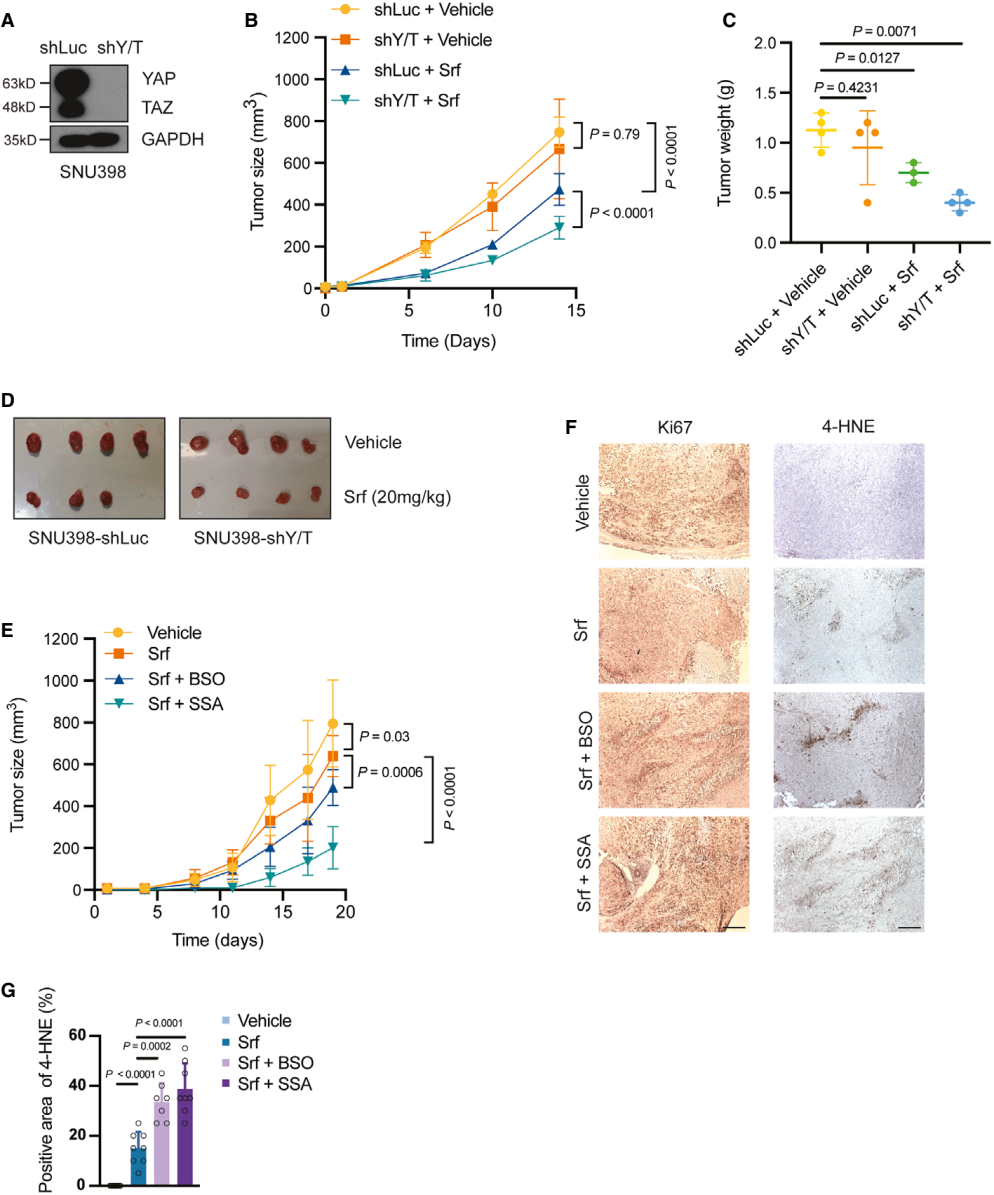
Targeting ferroptosis overcomes Sorafenib resistance AEstablishment of a YAP/TAZ‐deficient SNU398 cell line. SNU398 cells were infected with lentivirus to stably express either shLuc as control or shYAP/TAZ. Immunoblotting illustrated the loss of YAP/TAZ expression. GAPDH served as loading control.B–DCombination of YAP/TAZ deficiency and Sorafenib treatment suppressed tumor growth in a HCC xenograft model. SNU398‐shLuc or SNU398‐shYAP/TAZ (shY/T) cells were transplanted into the flanks of immunodeficient NSG mice. Once the tumors were palpable, mice were treated with 20 mg/kg Sorafenib or vehicle control, and tumor sizes were measured twice a week (B). Tumor weights and sizes were also recorded after sacrifice of the mice (C,D). Data are shown as mean ± standard deviation (SD). Statistical significance was calculated using two‐way ANOVA. Mouse numbers of shLuc + Vehicle, shY/T + Vehicle, and shY/T + Srf were 4 per cohort, mouse numbers of shLuc + Srf were 3 per cohort.EPharmacological inhibition of glutathione synthesis and function sensitizes HCC tumors to Sorafenib therapy. SNU398 cells were transplanted into the flanks of immunodeficient NSG mice. Once the tumors were palpable, mice were treated with vehicle control or with 20 mg/kg Sorafenib alone, or with 20 mg/kg Sorafenib and 20 mM BSO in the drinking water or 120 mg/kg SSA per os. Tumor sizes were measured twice a week. Data are shown as mean ± standard deviation (SD). Statistical significance was calculated using two‐way ANOVA. Mouse numbers were 8 for the experimental cohorts Vehicle, Srf, and Srf + SSA and 7 for Srf + BSO.FRepresentative pictures of immunohistochemical staining of Ki67 and 4‐HNE in tissue sections taken from HCC tumors of the mice treated as described in (E). Ki67 is a marker for cell proliferation, while 4‐HNE is a marker of lipid peroxidation and ferroptosis. Scale bars, 50 μm.GQuantification of 4‐HNE‐positive cells of the immunohistochemical stainings described in (F). Data are shown as mean ± standard deviation (SD). Statistical significance was calculated using one‐way ANOVA. Each data point represents the average of three technical replicates of one histological section, the numbers of tumor samples are given in (E). Source data are available online for this figure.

We then established SNU398 cells expressing either an shRNA against luciferase (SNU398‐shLuc) as control or an shRNA against YAP and TAZ (SNU398‐shY/T) (Fig 6A). These cells were then implanted into the flanks of NSG immunocompromised mice, and the mice were then treated with vehicle control or with Sorafenib (20 mg/kg). In line with the *in vitro* colony formation assays described above, YAP/TAZ deficiency resulted in an increased sensitivity to Sorafenib treatment *in vivo*, as reflected by decreased tumor growth rates as well as by smaller tumor masses at end point analysis (Fig 6B–D).

We further examined the potential therapeutic efficacy of targeting ferroptosis in order to overcome Sorafenib resistance. SNU398 cells were implanted into the flanks of NSG immunodeficient mice, and the mice were then treated with sulfasalazine (SSA) (Gout *et al*, 2001) (Wang *et al*, 2019), a pharmacological inhibitor of SLC7A11, or with buthionine sulfoximine (BSO) (Watanabe *et al*, 2003), a glutathione depletion reagent. Importantly, the combination therapy of Sorafenib with either compound led to delayed tumor growth (Fig 6E), suggesting that co‐targeting ferroptosis can efficiently sensitize tumors to Sorafenib therapy. Histochemical analysis of the tumors by 4‐hydroxynonenal (4‐HNE) staining confirmed an increase of lipid peroxidation in the inhibitor‐treated tumors, while tumor cell proliferation as determined by Ki67 staining was not significantly affected (Fig 6F and G).

Taken together, our studies demonstrate that the genetic ablation of YAP/TAZ or ATF4 or the pharmacologically blocking of their transcriptional target gene product SLC7A11 or the glutathione synthesis pathway can efficiently sensitize HCC tumors to Sorafenib therapy by unleashing ferroptosis to promote cell death.

## Discussion

**Figure 7:**
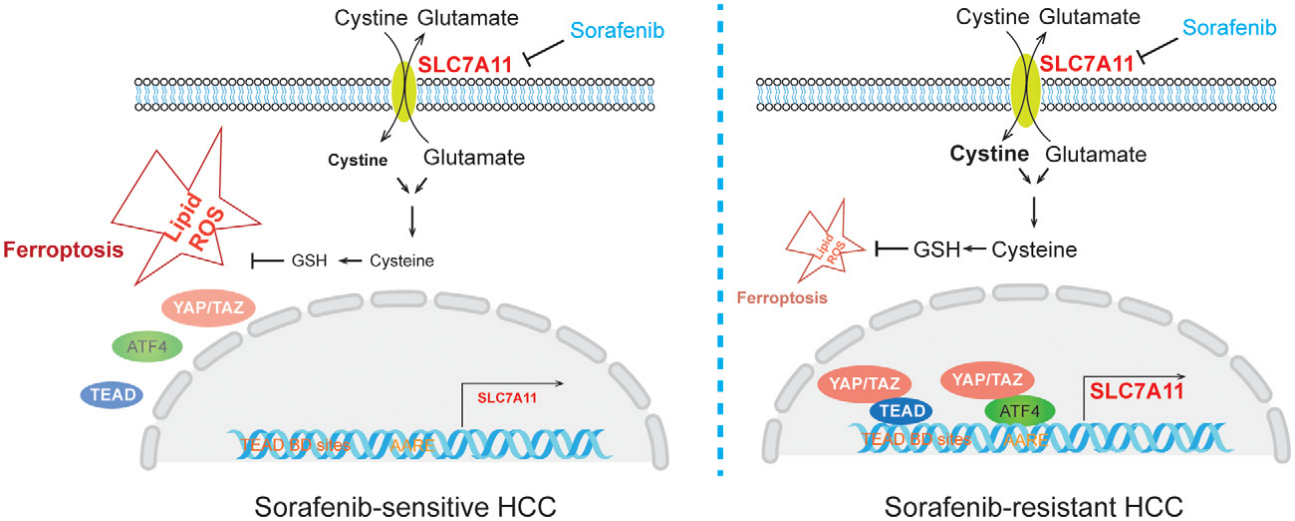
Working model of how YAP/TAZ and ATF4 repress ferroptosis in Sorafenib‐resistant HCC cells In Sorafenib‐sensitive cells, YAP/TAZ and ATF4 are not activated and not localized to the nucleus. As a consequence, the expression of antioxidant genes, such as *SLC7A11*, is not induced, and glutathione (GSH) levels are low. ROS levels then increase and ferroptosis can ensue, even more so in the presence of Sorafenib. In Sorafenib‐resistant cell, YAP/TAZ and ATF4 are activated in the nucleus and induce the expression of *SLC7A11* which increases GSH levels. ROS levels are thus reduced, ferroptosis is repressed, and HCC cells survive even in the presence of Sorafenib.

The development of therapy resistance is a general and sobering clinical challenge observed in a variety of therapeutic approaches, including newest molecularly targeted therapies. Sorafenib, a standard‐of‐care treatment for advanced HCC, targets cancer cells by blocking intracellular protein kinase cascades (Wilhelm *et al*, 2006). Further studies have uncovered that the cystine‐glutamate antiporter SLC7A11, a key antagonist of ferroptosis, is also inhibited by Sorafenib (Dixon *et al*, 2014). However, a recent report demonstrates that Sorafenib is not an inducer of ferroptosis and that inhibitors of SLC7A11 not necessarily induce ferroptosis in all tumor cells (Zheng *et al*, 2021). Hence, the mode of action of Sorafenib may be cell type‐specific and rely on its multiple roles in blocking proliferation and in inducing cell death. In our study, we have aimed at delineating the molecular drivers of Sorafenib resistance by combining a genome‐wide synthetic lethality screen with transcriptomic analysis. These combined approaches identified YAP/TAZ, well‐known transducers of the Hippo signaling pathway, as key factors in mediating Sorafenib resistance. One major mechanism relies on YAP/TAZ’s ability to restrain therapy‐induced ferroptosis by promoting *SLC7A11* gene expression via both TEAD‐dependent and ATF4‐dependent transcriptional activities (Fig 7).

Previous studies have suggested that *SLC7A11* can be transcriptionally regulated by a set of key oncogenic transcription factors, including mutant p53 (Jiang *et al*, 2015), NRF2 (Sun *et al*, 2016) and ATF4 (Chen *et al*, 2017). We here report that YAP/TAZ, as transcriptional co‐activators, form complexes with TEADs and thus indirectly bind to the TEAD motif in the *SLC7A11* gene promoter and thus induce the expression of SLC7A11. In line with previous report that ATF4 binds to AARE motif in the *SLC7A11* promoter (Sato *et al*, 2004), we observed that YAP/TAZ can bind to ATF4 and thus to the AARE motif in the *SLC7A11* gene promoter, illustrating that ATF4 serves as a novel transcriptional partner of YAP/TAZ. In fact, in addition to *SLC7A11*, global transcriptomic analysis revealed that YAP/TAZ and ATF4 share a substantial number of common direct and indirect transcriptional targets. While our current study focuses on *SLC7A11*, the functional contribution of ATF4 as an additional DNA binding partner of YAP/TAZ in physiological and pathophysiological processes warrants further investigation. Here, it will be also important to delineate the functional contribution of endoplasmic reticulum (ER) stress on the induction of ATF4 activity, in particular with the knowledge that Sorafenib is a well‐known inducer of ER stress (Rahmani *et al*, 2007; Shi *et al*, 2011).

In HCC cells, we have found that YAP/TAZ can form complexes with ATF4. The interaction of YAP/TAZ with ATF4 appears to promote the nuclear localization of ATF4 and thus its transcriptional activity and to stabilize ATF4 protein by preventing its cytoplasmic ubiquitylation and proteasomal degradation. Moreover, while TEADS have been previously shown to be critical for the nuclear import of YAP/TAZ (Diepenbruck *et al*, 2014), we here show that TEADs via YAP/TAZ are also required to direct ATF4 to the nucleus. Hence, our study suggests YAP/TAZ, and indirectly TEADs, as key chaperones for ATF4 upon cellular stress, such as in response to Sorafenib.

YAP/TAZ have been previously implicated in the development of resistance to targeted therapies as well as in epithelial tumor progression via regulating ferroptosis (Yang *et al*, 2020). In contrast, in breast tumors YAP has been shown to promote ferroptosis. Under cell–cell contact conditions, E‐cadherin suppresses ferroptosis by activating NF2 and the Hippo signaling pathway (Wu *et al*, 2019). Further, YAP transcriptionally upregulates key ferroptosis genes, such as ACSL4 and TFRC (Wu *et al*, 2019). Likewise, TAZ has been shown to promote ferroptosis in renal and ovarian tumors by upregulating EMP1‐NOX4 (Yang *et al*, 2019) and ANGPTL4‐NOX2 (Yang *et al*, 2020), respectively. A recent study by the Conrad laboratory demonstrated that Sorafenib fails to trigger ferroptosis in xCT^high^ cells (Zheng *et al*, 2021). In fact, the key conclusion from our study is that YAP/TAZ and ATF4 drive high levels of SLC7A11 expression, thereby restricting Sorafenib‐induced ferroptosis. Thus, our conclusion is fully supported by the recently published study. Similar to HCC cells, we have also found that YAP/TAZ and ATF4 repress ferroptosis by inducing SLC7A11 expression in other cancer cell types, including HT1080 fibrosarcoma and MDA‐MB‐231 breast cancer cells, suggesting a potentially high generality of the results. However, we found differences in the response of the various cancer cell types to different ferroptosis inducers, and our and the published results together suggest that the roles of YAP/TAZ and ATF4 in ferroptosis regulation appear to be cell context‐dependent. Here, detailed investigations are warranted to molecularly segregate the promoting and inhibitory roles of Hippo signaling in ferroptosis and the consequences for physiological and pathophysiological processes.

Our studies have identified a novel role of YAP/TAZ and ATF4 in restraining ferroptosis during the development of resistance against Sorafenib therapy in HCC, most likely also in patients. In its sum, the work raises the possibility and provides a first proof of concept in mouse models that targeting YAP/TAZ or ATF4 may be a suitable way to overcome therapy resistance. In fact, small inhibitors targeting YAP/TAZ have been and are being explored as anti‐cancer agents at a pre‐clinical and clinical stage. Notably, a vast majority of inventions aim to disrupt TEAD‐dependent YAP/TAZ function in tumor progression, such as blocking TEAD palmitoylation. Our observations of ATF4‐dependent YAP/TAZ activities, together with previous findings of and interaction of YAP/TAZ and β‐catenin and SMADs, suggest that targeting YAP/TAZ themselves, such as via a PROTAC approach, might represent a most efficient approach in clinics. Finally, in the context of Sorafenib resistance as a major clinical challenge in the therapy of HCC, we report first pre‐clinical proof that the specific targeting of enzymes and transporters involved in glutathione synthesis and homeostasis may offer attractive opportunities to overcome Sorafenib and potentially other therapy resistance.

## Materials and Methods

### DNA constructs, siRNAs, and antibodies

1C‐TAZ‐WT, 1C‐TAZ‐4SA, 1C‐TAZ‐4SA‐mNLS were kind gifts from Andras Kapus. pBabe‐puro‐HA‐EV, and pBabe‐puro‐HA‐YAP‐5SA were kind gifts from Alex Hergovich. pLenti‐CMV‐SLC7A11‐sh926R‐FLAG‐IRES‐Hygro was purchased from Addgene (# 118702). To generate pSuper‐retro‐puro‐shYAP/TAZ construct, the oligonucleotide 5′‐TGTGGATGAGATGGATACA‐3′ which targets both YAP and TAZ mRNAs was cloned into pSuper‐retro‐puro vector. To generate pGL4.10‐SLC7A11, a DNA fragment from −1,000 bp to +200 bp containing the *SLC7A11* promoter was cloned into the pGL4.10 vector.

On‐target siRNAs were purchased from Horizon Discovery and are listed in Appendix Table S1. Antibodies used are listed in Appendix Table S2, and PCR primers are listed in Appendix Table S3.

### Cell culture, transfection, reagents, and stable cell line generation

HEK‐293T, SNU398, HT1080 cells were obtained from American Type Culture Collection (ATCC); Huh7, HLE, Hep3B were gifts from Luca Quagliata. All cell lines used in this study were authenticated when applicable and tested for the absence of mycoplasma contamination every 2 weeks. Cells were cultured with DMEM supplemented with fetal calf serum (FCS, 10%, Sigma‐Aldrich), l‐glutamine (2 mM, Sigma‐Aldrich), penicillin (100 U/ml, Sigma‐Aldrich), and streptomycin (100 μg/ml, Sigma‐Aldrich) in the incubator at 5% CO_2_, 37°C, 95% humidity. Plasmid transfections into HEK293T cells were carried out by using PEI (polyethylenimine, linear, MW 25000, Polysciences Catalog No. 23966‐1), plasmid transfections into HCC cell lines were carried out with jetPEI (polyPlus Transfections) or with Lipofectamine 3000 (Invitrogen). siRNA transfections were carried out with Lipofectamine RNAiMAX (Invitrogen) according to the manufacturer’s instructions.

To establish cell lines stably overexpressing SLC7A11, lentivirus was produced by transfection of HEK‐293T cells with pLenti‐CMV‐SLC7A11‐sh926R‐FLAG‐IRES‐Hygro, infections were performed using 8 μg/ml polybrene (Sigma #107689), hygromycin B (Invivogen, # ant‐hg‐1) was used for selection. To establish YAP/TAZ‐stable knockdown cell lines, retrovirus was produced by transfection of Platinium‐A cells (Cell Biolabs) with pSuper‐retro‐puro‐shYAP/TAZ, infections were performed using 8 μg/ml Polybrene (Sigma #107689), and puromycin (Invivogen, # ant‐pr‐5b) was used for the selection. To establish YAP‐5SA stable overexpressing cell lines, retrovirus was produced by transfection of Platinium‐A cells (Cell Biolabs) with pBabe‐puro‐HA‐YAP‐5SA, infections were performed using 8 μg/ml Polybrene (Sigma #107689), and puromycin (Invivogen, # ant‐pr‐5b) was used for the selection. The shRNA sequence to target both YAP and TAZ expression was taken from (Hiemer *et al*, 2015).

The following reagents were used to modulate cell death: Sorafenib (Selleck‐S7397), (Ferrostatin‐1 (Sigma‐SML0583), GSK872 (Selleck‐S8465), Z‐VAD‐FMK (Selleck‐S7023), Erastin (Sigma‐E7781), RSL3 (Sigma‐SML2234), FIN56 (Sigma‐SML1740), and FIN02 (CAY‐25096).

### Synthetic‐lethal screen

A barcoded shRNA library targeting 6317 mRNAs (50467 shRNA) involved in signaling pathways (human genome‐wide pooled lentiviral shRNA library module 1, vector: pRSI16cb, Cellecta) was introduced into Sorafenib‐resistant cells by lentiviral transduction at a MOI = 0.5. Cells were treated with 2 μg/ml puromycin to select for infected cells. Cells were cultured with 7 μM Sorafenib, and the medium was refreshed three times a week for 4 weeks to generate sufficient cell numbers. Genomic DNA was extracted using QIAGEN QIAamp DNA Micro Kit (QIAGEN, #56304). shRNA barcodes were amplified with NGS Prep Kit (For shRNA Libraries in pRSI16cb (KOHGW), Biocat, # LNGS‐120‐CT). PCR products were purified with the QIAquick PCR purification kit (QIAGEN, #28106) and separated by electrophoresis in 3.5% agarose gels; exact bands were excised and purified using QIAquick gel extraction kit (QIAGEN, #28706). DNA concentrations were measured and adjusted to 10 nM; next‐generation sequencing (NGS) of pooled amplified barcodes was performed on an Illumina HiSeq (Illumina).

### Cell viability assay

Cells were seeded into 96‐well plates (5,000 cells per well), treated with different inducers or inhibitors before harvest. Cell viability was measured using the CellTiter‐Glo^®^ 2.0 kit (Promega G9243) according to the manufacturer’s recommendations. Luminescence signals were recorded with a bioluminescence plate reader (Berthold Centro LB 960).

### Colony formation assay

Cells were seeded into 12‐well plates (5,000 cells per well) and cultured for 2 weeks, siRNAs were transfected every other day, and culture medium with either DMSO or Sorafenib was exchanged every 24 h. 2 weeks later, cells were washed with PBS and fixed with 4% paraformaldehyde for 30 min at room temperature, washed with PBS again, and stained with crystal violet (1 mg/ml dissolved into 10% ethanol) for 30 min at room temperature. After washing with PBS, plates were left to dry and cells stained with crystal violet were quantified using Fiji (NIH Image).

### Dual‐Luciferase report assay

Cells were seeded into 24‐well plates, siRNAs were transfected once cell confluence had reached 60%. Medium was changed after 6 h. Twenty‐four hours later, pGL4.10‐SLC7A11 and pRL‐CMV were transfected together into cells at a 10:1 mass ratio, and medium was changed after 6 h. Cells were washed with PBS twice, and *Firefly* luminescence and *Renilla* luminescence were measured using the Dual‐Luciferase report Assay Kit (Promega E1980) and a bioluminescence plate reader (Berthold Centro LB 960).

### Intercellular GSH assay

Cells were seeded into 96‐well plates (5,000 cells per well), treated with DMSO or 6 μM Sorafenib, respectively. Eighteen hours later, cells were washed with PBS twice and harvested by trypsinization. Cell numbers were counted with a cell counter. Intracellular GSH levels were measured using the GSH‐Glo Glutathione Assay kit (Promega V6911/V6912) and a bioluminescence plate reader (Berthold Centro LB 960). The results were normalized to cell numbers.

### Cystine uptake measurement

10^6^ cells were seeded into 60‐mm dishes. Twenty‐four hours later, cells were transfected with siRNAs, and medium was exchanged 6 h after the transfection. Twenty‐four hours after the transfection, cells were treated with DMSO, Sorafenib, or Erastin for 18 h. Cystine‐FITC (BioTracker Cystine‐FITC Live Cell Dye, Sigma SCT047) was diluted in medium at a concentration of 5 μM and added to cells to incubate at 37°C for 30 min. Cells were washed with PBS and harvested by trypsinization. Cells were resuspended in PBS and filtered through a 40‐μm strainer. Intracellular Cystine‐FITC levels were measured by flow cytometry (Beckman Coulter Cytoflex LX) with a 488 nm laser. Data were collected and analyzed using Flow Jo (Ashland, Oregon‐based FlowJo LLC).

### Cellular lipid measurement

Cells were seeded into 60‐mm dishes with 10^6^ cells. Twenty‐four hours later, cells were transfected with siRNAs, and fresh medium was changed 6 h after the transfection. Twenty‐four hours after transfection, cells were treated with DMSO or Sorafenib for 18 h. C11‐BODIPY 581/591 (Thermo Fisher Scientific, D3861) was used to stain the cells for 30 min at 37°C and harvested by trypsinization. Cells were resuspended in PBS and filtered through a 40‐μM strainer. Flow cytometry (Beckman Coulter Cytoflex LX) with 488 and 561 nm lasers was used for analysis, and data were collected and analyzed using Flow Jo (Ashland, Oregon‐based FlowJo LLC).

### Cellular Reactive Oxygen Species (ROS) measurement

10^6^ cells were seeded into 60‐mm dishes. Twenty‐four hours later, cells were transfected with siRNAs and fresh medium were changed 6 h after the transfection. Twenty‐four hours after the transfection, cells were treated with DMSO, Sorafenib or Erastin for 18 h. CellROX™ Green Flow Cytometry Assay Kit (Thermo Fisher Scientific, C10492) was used to stain the cells for 30 min at 37°C which were then harvested by trypsinization. Cells were resuspended in PBS and filtered through a 40‐μM strainer. Flow cytometry (Beckman Coulter Cytoflex LX) with a 488 nm laser was used for analysis, and data were collected and analyzed using Flow Jo (Ashland, Oregon‐based FlowJo LLC).

### Protein lysis, immunoprecipitation, ubiquitination assay

For immunoblotting analysis, cells were washed with 1× PBS twice and lysed with RIPA lysis buffer (Sigma R0278). Cell lysates were centrifuged and the pellets were removed before protein concentration measurement and immunoblotting analysis.

For immunoprecipitation, cells were washed with 1× PBS twice and lysed with CST lysis buffer (CST9803) supplemented with protease inhibitors (Sigma P2714) at 4°C, then centrifuged at 16,100 *g* for 10 min, and pellets were removed. 1/10 of the cell lysate was taken as input, the rest of the cell lysate was incubated with specific antibodies and protein A/G‐Sepharose overnight at 4°C. After five times washing with CST lysis buffer, the precipitated proteins were eluted with SDS‐loading buffer and analyzed by immunoblotting.

For ubiquitination assays, cells transfected with plasmids were lysed with RIPA buffer supplemented with additional 0.1% SDS to a final concentration of 0.2% SDS, followed by standard immunoprecipitation protocols.

For immunoblotting analysis, protein samples were fractionated by SDS–PAGE and transferred to PVDF membranes, then membranes were blocked with 5% skimmed milk in TBST, and antibodies were incubated with the membranes overnight at 4°C. Membranes were washed with TBST 3 × 10 min and incubated with the secondary antibodies for 2 h at room temperature, then washed for 3 × 10 min with TBST. Chemiluminescence was detected with X‐ray films (FUJIFILM) or using Fusion (Analis) once the membranes were incubated with chemiluminescent HRP substrate (Millipore WBKLS0500). Fiji software was used to quantify the immunoblots by densitometry (NIH Image). Information on the antibodies used is presented in Appendix Table S2.

### Chromatin immunoprecipitation

Cells were washed with cold PBS twice, crosslinking was conducted with 1% formaldehyde and EGS (Thermo Fisher #21565). Glycine was used to quench the reaction, cells were then rinsed three times with cold PBS and scraped off from dishes for centrifugation. Cells were lysed with CHIP lysis buffer and sonicated (Bioruptor, Diagenode) to achieve 100–500 bps chromatin fragments, sonication efficacy was validated by running 1% agarose gel. 150 μg chromatin in 600 μl CHIP dilution buffer was prepared, 1% was saved for input samples, and the rest of the chromatin was incubated with 5 μg normal rabbit IgG antibody (Cell Signaling Technology, 2729) or anti‐YAP1 antibody (Cell Signaling Technology, 4912), anti‐TAZ (V386) antibody (Cell Signaling Technology, 4883), or anti‐ATF‐4 (D4B8) antibody (Cell Signaling Technology, 11815) overnight at 4°C. Immunocomplexes were incubated with 30 μl pre‐locked Sepharose Protein A beads (Affi‐Prep Protein A Support, Bio‐Rad; 1560006) at 4°C, 2 h later the immunocomplexes were centrifuged at 9,500 *g* for 1 min and the supernatant discarded. The beads were washed with CHIP wash buffer. Immunocomplexes were eluted from the beads, for Re‐CHIP experiment, after the first step elution, half of the samples were incubated with another antibody and pre‐locked Sepharose Protein A beads for the secondary immunoprecipitation. Input and IP samples were all de‐crosslinked and purified with QIAquick Gel Extraction kit (Qiagen, #28704), and eluted DNA samples were analyzed by quantitative PCR. Fold enrichments for specific *SLC7A11* promoter regions were calculated by IP over input samples and normalized to isotype‐specific IgG as the negative control. Enrichments of *CYR61* promoter were used as a positive control. Primers used in the ChIP experiment are listed in Appendix Table S3.

### RNA extraction and quantitative RT‐PCR

RNA was extracted with TRIZOL reagent (Sigma T9424), reverse transcription PCR was performed using Reverse Transcriptase kit (Promega A3803), and real‐time PCR was performed using Powerup SYBR Green PCR master mix (A25743) on a Step‐One Plus real‐time PCR machine (Applied Biosystems). Human RPL19 expression was used for normalization. Fold changes were calculated using the comparative Ct method (ΔΔCt). Sequences of primers are listed in Appendix Table S3.

### Immunohistochemistry

Tumor sections were deparaffinized with 3 × 10 min Roticlear, 2 × 5 min 100% EtOH, 1 × 10 min 90% EtOH, 1 × 5 min 80% EtOH, 1 × 5 min 70% EtOH, 1 × 5 min 30% EtOH, 3 × 10 min PBS. Antigen retrieval was performed in 10 mM pH 6.0 citrate buffer in a pressure cooker, then washed 3 × 10 min with 0.3% Triton‐100 in PBS. Peroxidase was quenched with 3% H_2_O_2_ for 10 min, washed with PBS 3 × 10 min, then blocked with 2.5% goat serum for 30 min at room temperature. Incubation with primary antibody (diluted into 2.5% goat serum) was at 4°C overnight, followed by washing 3 × 10 min with PBS, incubation with secondary antibody (vector MP‐7541‐50) at room temperature for 30 min, washing 3 × 10 min with PBS, incubation with peroxidase substrate (vector SK‐4105) at room temperature for 5 min and washing with water for 5 min. Counterstaining with hematoxylin was done for 1 min to stain nuclei, followed by washing with water for 5 min, and dehydration with 50% EtOH, 70% EtOH, and 95% EtOH for 5 min each, then 2 × 10 min 100% EtOH, and clearing with 2 × 10 min xylene. Coverslips were mounted with 2–3 drops mounting media (Thermo Fisher Scientific Cytoseal™ XYL mounting media 8312‐4) and let dry overnight. Slides were imaged using a Zeiss brightfield microscope (Zeiss Axioskop 2 Plus) and analyzed with Fiji (NIH Image).

### Immunohistochemistry of patient samples

Immunohistochemical staining of SLC7A11 was assessed on a tissue multi‐array (TMA) of an independent cohort of 233 HCCs, 119 cirrhotic tissues, and 19 normal liver samples, as previously described (Andreozzi *et al*, 2016). Additionally, SLC7A11 and YAP1 staining were performed in 10 HCC patients using whole serial tissue slides. Similarly, YAP and ATF4 staining were performed in a cohort of 18 HCC patients using whole serial tissue slides. Staining was performed on a Leica Bond III immunohistochemistry staining system (Muttenz, Switzerland) using DAB as chromogen as previously described (Coto‐Llerena *et al*, 2020). Antigen retrieval was performed using HIER 2 solution. Primary antibodies were used at the following concentrations: anti‐SLC7A11 (1:50, Cell Signaling clone 12691S), YAP (1:500, Abcam ab52771), and ATF4 (1:100, Abcam ab221791).

All relevant ethical regulations were strictly followed in this study. All the analyses using human tissue samples reported in this study were approved by the ethics commission of Northwestern Switzerland (EKNZ, approval no. 361/12). Informed consent was obtained from all subjects and the experiments conformed to the principles set out in the WMA Declaration of Helsinki and the Department of Health and Human Services Belmont Report.

### Immunofluorescence

Cells were cultured on coverslips, washed with PBS twice and fixed with 4% paraformaldehyde for 10 min, and then washed twice with PBS. Cells were permeabilized with 0.1% Triton (DAPI was also diluted into Triton at 100 ng/ml to stain the nucleus) on ice for 10 min. After three times washing with PBS, cells were blocked with 5% goat serum for 1 h at room temperature, then incubated with diluted antibodies (in 5% goat serum) overnight at 4°C. Cells were washed with PBS three times then incubated with secondary antibody (1:200 dilution) at room temperature for 1 h. Then, cells were washed with PBS three times, and mounting medium was added to mount coverslips on glass slides. Immunofluorescence staining was visualized using a Leica DMI 4000/6000 microscope.

### Tumor transplantation

All animal experiments were performed according to Swiss Federal Animal Welfare Law under approval number 2839 by the Veterinary Office of the Canton Basel‐Stadt. 2‐ to 3 month‐old male immunodeficient NOD/SCID; common γ receptor^−/−^ (NSG) mice were used in this study for tumor transplantation experiments. The mouse line was obtained from the Department of Biomedicine, University of Basel, animal facility and bred and housed in a certified animal facility with a 12‐h light/dark cycle in a temperature‐controlled room (22 ± 1°C) with free access to water and food, in accordance with Swiss guidelines.

SNU398‐parental cells, SNU398‐shLuc, or SNU398‐shYAP/TAZ cells (10^6^ in 100 μl PBS) were implanted into the left flanks of immunodeficient NOD/SCID; common γ receptor^−/−^ (NSG) mice. When tumors were palpable, Sorafenib (LC Laboratories, S‐8502) was applied at 20 mg/kg daily via gavage, SSA (Sulfasalazine, Sigma, S0883) was given at 120 mg/kg daily by intraperitoneal injection, 20 mM BSO (L‐buthionine‐sulfoximine, Sigma, B2515) was given in the drinking water for 3 weeks. Tumor width and length were measured twice a week using a caliper, and tumor volumes were calculated using the formulation of volume = length × width^2^ × 0.52.

### RNA‐sequencing analysis

RNA was extracted in biological triplicates using miRNeasy Mini kit (Qiagen) according to the manufacturer’s instructions. RNA quality control was performed using a fragment analyzer and standard or high‐sensitivity RNA analysis kits (Labgene; DNF‐471‐0500 or DNF‐472‐0500). RNA concentrations were measured using Quanti‐iTTM RiboGreen RNA assay Kit (Life Technologies/Thermo Fisher Scientific). A total of 200ng of RNA was utilized for library preparation using TruSeq stranded total RNA LT sample prep Kit (Illumina). Poly‐A^+^ RNA was sequenced using HiSeq SBS Kit v4 (Illumina) on an Illumina HiSeq 2500 using protocols defined by the manufacturer.

Single‐end RNA‐seq reads (81‐mers) were mapped to the human genome assembly, version hg19 (GRCh37.75), with RNA‐STAR (Dobin *et al*, 2013), with default parameters except for allowing only unique hits to genome (outFilterMultimapNmax=1) and filtering reads without evidence in spliced junction table (outFilterType="BySJout"). Expression levels per gene (counts over exons) for the RefSeq mRNA coordinates from UCSC (genome.ucsc.edu, downloaded in December 2015) were quantified using qCount function from QuasR package (version 1.12.0). The differentially expressed genes were identified using the edgeR package (version 3.14.0). Genes with *P*‐values smaller than 0.05 and minimum log2‐fold changes of +/−0.58 were considered as differentially regulated and were selected for functional and pathway enrichment analysis.

### Functional enrichment analysis

Functional enrichment analysis of differentially expressed genes for biological processes or pathways was performed in R using several publicly available Bioconductor resources, including org. Hs.eg.db (version 3.3.0), GO.db (version 3.4.1), GOstats (version 2.42.0) (Falcon & Gentleman, 2007), KEGG.db (version 3.2.3), and ReactomePA (version 1.16.2) (Yu & He, 2016). The significance of each biological processes or pathways identified was calculated using the hypergeometric test (equivalent to Fisher’s exact test) and those with *P* ≤ 0.05 were considered significant.

### Gene set enrichment analysis (GSEA)

GSEA analysis was performed using the JAVA application of the Broad Institute version 3.0 (http://www.broadinstitute.org/gsea). The gene sets used for the analysis were derived from gene ontology annotations, and pathways were obtained from the Kyoto Encyclopedia of Genes and Genomes (KEGG) (http://www.genome.jp/kegg/) databases.

### Re‐analysis of transcriptomic profiling data

RNA‐sequencing gene expression values were retrieved from om TCGA Liver dataset (Cancer Genome Atlas Research Network. Electronic address & Cancer Genome Atlas Research, 2017) using cbioportal (http://www.cbioportal.org, accessed 05/12/2017) (Gao *et al*, 2013). The dataset included 364 and 314 HCCs with overall survival and disease‐free survival information, respectively. Survival analyses were performed using Kaplan‐Meier method and log‐rank test. The cutoff was defined as previously described (Budczies *et al*, 2012).

### Statistical analysis

All statistical tests were two‐sided. Data are presented as means. Bar plots with error bars represent means ± standard derivations (SD). All analyses were performed using Prism 8.0.

## Author contributions

FT and GC conceived this study. RG and FT designed and performed the experiments, analyzed the data, and wrote the manuscript. GC designed the experiments and wrote the manuscript. DB and FT established cell lines. RG and FT performed the synthetic lethal screen, SS helped to conduct the ChIP experiments. RKRK analyzed the RNA‐sequencing data. MC‐L, CE, and SP performed the histopathological analysis. MTD and FDC analyzed mouse samples.

## Conflict of interest

The authors declare that they have no conflict of interest.

## Supporting information



Appendix

Dataset EV1

Dataset EV2

Dataset EV3

Dataset EV4

Dataset EV5

Source Data for Appendix

Source Data for Figure 1

Source Data for Figure 2

Source Data for Figure 3

Source Data for Figure 4

Source Data for Figure 5

Source Data for Figure 6

## Data Availability

The sequencing files are deposited on GEO database (https://www.ncbi.nlm.nih.gov/geo/browse/) with the accession numbers: GSE117116 (RNA‐sequencing Sorafenib‐resistant cell lines), GSE158458 (synthetic lethal barcode sequencing), and GSE181771 (RNA sequencing of YAP/TAZ and ATF4 knockdown cells).

## References

[emmm202114351-bib-0001] Andreozzi M , Quintavalle C , Benz D , Quagliata L , Matter M , Calabrese D , Tosti N , Ruiz C , Trapani F , Tornillo L *et al* (2016) HMGA1 expression in human hepatocellular carcinoma correlates with poor prognosis and promotes tumor growth and migration in *in vitro* models. Neoplasia 18: 724–731 27855356 10.1016/j.neo.2016.10.002 PMC5110473

[emmm202114351-bib-0002] Budczies J , Klauschen F , Sinn BV , Gyorffy B , Schmitt WD , Darb‐Esfahani S , Denkert C (2012) Cutoff finder: a comprehensive and straightforward Web application enabling rapid biomarker cutoff optimization. PLoS One 7: e51862 23251644 10.1371/journal.pone.0051862 PMC3522617

[emmm202114351-bib-0003] Cancer Genome Atlas Research Network. Electronic address web, Cancer Genome Atlas Research N (2017) Comprehensive and integrative genomic characterization of hepatocellular carcinoma. Cell 169: 1327–1341 e1323 28622513 10.1016/j.cell.2017.05.046 PMC5680778

[emmm202114351-bib-0004] Chen D , Fan Z , Rauh M , Buchfelder M , Eyupoglu IY , Savaskan N (2017) ATF4 promotes angiogenesis and neuronal cell death and confers ferroptosis in a xCT‐dependent manner. Oncogene 36: 5593–5608 28553953 10.1038/onc.2017.146 PMC5633655

[emmm202114351-bib-0005] Coto‐Llerena M , Ercan C , Kancherla V , Taha‐Mehlitz S , Eppenberger‐Castori S , Soysal SD , Ng CKY , Bolli M , von Flüe M , Nicolas GP *et al* (2020) High expression of FAP in colorectal cancer is associated with angiogenesis and immunoregulation processes. Front Oncol 10: 979 32733792 10.3389/fonc.2020.00979 PMC7362758

[emmm202114351-bib-0006] Diepenbruck M , Waldmeier L , Ivanek R , Berninger P , Arnold P , van Nimwegen E , Christofori G (2014) Tead2 expression levels control the subcellular distribution of Yap and Taz, zyxin expression and epithelial‐mesenchymal transition. J Cell Sci 127: 1523–1536 24554433 10.1242/jcs.139865

[emmm202114351-bib-0007] Dixon S , Lemberg K , Lamprecht M , Skouta R , Zaitsev E , Gleason C , Patel D , Bauer A , Cantley A , Yang W *et al* (2012) Ferroptosis: an iron‐dependent form of nonapoptotic cell death. Cell 149: 1060–1072 22632970 10.1016/j.cell.2012.03.042 PMC3367386

[emmm202114351-bib-0008] Dixon SJ , Patel DN , Welsch M , Skouta R , Lee ED , Hayano M , Thomas AG , Gleason CE , Tatonetti NP , Slusher BS *et al* (2014) Pharmacological inhibition of cystine‐glutamate exchange induces endoplasmic reticulum stress and ferroptosis. Elife 3: e02523 24844246 10.7554/eLife.02523 PMC4054777

[emmm202114351-bib-0009] Dobin A , Davis CA , Schlesinger F , Drenkow J , Zaleski C , Jha S , Batut P , Chaisson M , Gingeras TR (2013) STAR: ultrafast universal RNA‐seq aligner. Bioinformatics 29: 15–21 23104886 10.1093/bioinformatics/bts635 PMC3530905

[emmm202114351-bib-0010] Falcon S , Gentleman R (2007) Using GOstats to test gene lists for GO term association. Bioinformatics 23: 257–258 17098774 10.1093/bioinformatics/btl567

[emmm202114351-bib-0011] Gao J , Aksoy BA , Dogrusoz U , Dresdner G , Gross B , Sumer SO , Sun Y , Jacobsen A , Sinha R , Larsson E *et al* (2013) Integrative analysis of complex cancer genomics and clinical profiles using the cBioPortal. Sci Signal 6: pl1 23550210 10.1126/scisignal.2004088 PMC4160307

[emmm202114351-bib-0012] Gao R , Buechel D , Kalathur RKR , Morini MF , Coto‐Llerena M , Ercan C , Piscuoglio S , Chen Q , Blumer T , Wang X *et al* (2021) USP29‐mediated HIF1alpha stabilization is associated with Sorafenib resistance of hepatocellular carcinoma cells by upregulating glycolysis. Oncogenesis 10: 52 34272356 10.1038/s41389-021-00338-7 PMC8285469

[emmm202114351-bib-0013] Gout PW , Buckley AR , Simms CR , Bruchovsky N (2001) Sulfasalazine, a potent suppressor of lymphoma growth by inhibition of the x(c)‐ cystine transporter: a new action for an old drug. Leukemia 15: 1633–1640 11587223 10.1038/sj.leu.2402238

[emmm202114351-bib-0014] Harvey KF , Zhang X , Thomas DM (2013) The Hippo pathway and human cancer. Nat Rev Cancer 13: 246–257 23467301 10.1038/nrc3458

[emmm202114351-bib-0015] Heallen T , Zhang M , Wang J , Bonilla‐Claudio M , Klysik E , Johnson RL , Martin JF (2011) Hippo pathway inhibits Wnt signaling to restrain cardiomyocyte proliferation and heart size. Science 332: 458–461 21512031 10.1126/science.1199010 PMC3133743

[emmm202114351-bib-0016] Hiemer SE , Zhang L , Kartha VK , Packer TS , Almershed M , Noonan V , Kukuruzinska M , Bais MV , Monti S , Varelas X (2015) A YAP/TAZ‐regulated molecular signature is associated with oral squamous cell carcinoma. Mol Cancer Res 13: 957–968 25794680 10.1158/1541-7786.MCR-14-0580 PMC4470857

[emmm202114351-bib-0017] Jiang L , Kon N , Li T , Wang SJ , Su T , Hibshoosh H , Baer R , Gu W (2015) Ferroptosis as a p53‐mediated activity during tumour suppression. Nature 520: 57–62 25799988 10.1038/nature14344 PMC4455927

[emmm202114351-bib-0018] Jiang X , Stockwell BR , Conrad M (2021) Ferroptosis: mechanisms, biology and role in disease. Nat Rev Mol Cell Biol 22: 266–282 33495651 10.1038/s41580-020-00324-8 PMC8142022

[emmm202114351-bib-0019] Kim YS , Jin HO , Seo SK , Woo SH , Choe TB , An S , Hong SI , Lee SJ , Lee KH , Park IC (2011) Sorafenib induces apoptotic cell death in human non‐small cell lung cancer cells by down‐regulating mammalian target of rapamycin (mTOR)‐dependent survivin expression. Biochem Pharmacol 82: 216–226 21601561 10.1016/j.bcp.2011.04.011

[emmm202114351-bib-0020] Kudo M , Finn RS , Qin S , Han K‐H , Ikeda K , Piscaglia F , Baron A , Park J‐W , Han G , Jassem J *et al* (2018) Lenvatinib versus sorafenib in first‐line treatment of patients with unresectable hepatocellular carcinoma: a randomised phase 3 non‐inferiority trial. Lancet 391: 1163–1173 29433850 10.1016/S0140-6736(18)30207-1

[emmm202114351-bib-0021] Lachaier E , Louandre C , Godin C , Saidak Z , Baert M , Diouf M , Chauffert B , Galmiche A (2014) Sorafenib induces ferroptosis in human cancer cell lines originating from different solid tumors. Anticancer Res 34: 6417–6422 25368241

[emmm202114351-bib-0022] Lee MS , Ryoo B‐Y , Hsu C‐H , Numata K , Stein S , Verret W , Hack SP , Spahn J , Liu BO , Abdullah H *et al* (2020) Atezolizumab with or without bevacizumab in unresectable hepatocellular carcinoma (GO30140): an open‐label, multicentre, phase 1b study. Lancet Oncol 21: 808–820 32502443 10.1016/S1470-2045(20)30156-X

[emmm202114351-bib-0023] Llovet JM , Ricci S , Mazzaferro V , Hilgard P , Gane E , Blanc J‐F , de Oliveira AC , Santoro A , Raoul J‐L , Forner A *et al* (2008) Sorafenib in advanced hepatocellular carcinoma. N Engl J Med 359: 378–390 18650514 10.1056/NEJMoa0708857

[emmm202114351-bib-0024] Llovet JM , Zucman‐Rossi J , Pikarsky E , Sangro B , Schwartz M , Sherman M , Gores G (2016) Hepatocellular carcinoma. Nat Rev Dis Primers 2: 16018 27158749 10.1038/nrdp.2016.18

[emmm202114351-bib-0025] Pan D (2010) The hippo signaling pathway in development and cancer. Dev Cell 19: 491–505 20951342 10.1016/j.devcel.2010.09.011 PMC3124840

[emmm202114351-bib-0026] Rahmani M , Davis EM , Crabtree TR , Habibi JR , Nguyen TK , Dent P , Grant S (2007) The kinase inhibitor sorafenib induces cell death through a process involving induction of endoplasmic reticulum stress. Mol Cell Biol 27: 5499–5513 17548474 10.1128/MCB.01080-06 PMC1952105

[emmm202114351-bib-0027] Sato H , Nomura S , Maebara K , Sato K , Tamba M , Bannai S (2004) Transcriptional control of cystine/glutamate transporter gene by amino acid deprivation. Biochem Biophys Res Commun 325: 109–116 15522208 10.1016/j.bbrc.2004.10.009

[emmm202114351-bib-0028] Shi Y‐H , Ding Z‐B , Zhou J , Hui BO , Shi G‐M , Ke A‐W , Wang X‐Y , Dai Z , Peng Y‐F , Gu C‐Y *et al* (2011) Targeting autophagy enhances sorafenib lethality for hepatocellular carcinoma via ER stress‐related apoptosis. Autophagy 7: 1159–1172 21691147 10.4161/auto.7.10.16818

[emmm202114351-bib-0029] Sun X , Ou Z , Chen R , Niu X , Chen D , Kang R , Tang D (2016) Activation of the p62‐Keap1‐NRF2 pathway protects against ferroptosis in hepatocellular carcinoma cells. Hepatology 63: 173–184 26403645 10.1002/hep.28251 PMC4688087

[emmm202114351-bib-0030] Tang F , Gao R , Jeevan‐Raj B , Wyss CB , Kalathur RKR , Piscuoglio S , Ng CKY , Hindupur SK , Nuciforo S , Dazert E *et al* (2019) LATS1 but not LATS2 represses autophagy by a kinase‐independent scaffold function. Nat Commun 10: 5755 31848340 10.1038/s41467-019-13591-7 PMC6917744

[emmm202114351-bib-0031] Totaro A , Panciera T , Piccolo S (2018) YAP/TAZ upstream signals and downstream responses. Nat Cell Biol 20: 888–899 30050119 10.1038/s41556-018-0142-z PMC6186418

[emmm202114351-bib-0032] Wang W , Green M , Choi JE , Gijón M , Kennedy PD , Johnson JK , Liao P , Lang X , Kryczek I , Sell A *et al* (2019) CD8(+) T cells regulate tumour ferroptosis during cancer immunotherapy. Nature 569: 270–274 31043744 10.1038/s41586-019-1170-y PMC6533917

[emmm202114351-bib-0033] Watanabe T , Sagisaka H , Arakawa S , Shibaya Y , Watanabe M , Igarashi I , Tanaka K , Totsuka S , Takasaki W , Manabe S (2003) A novel model of continuous depletion of glutathione in mice treated with L‐buthionine (S, R)‐sulfoximine. J Toxicol Sci 28: 455–469 14746349 10.2131/jts.28.455

[emmm202114351-bib-0034] Wilhelm S , Carter C , Lynch M , Lowinger T , Dumas J , Smith RA , Schwartz B , Simantov R , Kelley S (2006) Discovery and development of sorafenib: a multikinase inhibitor for treating cancer. Nat Rev Drug Discov 5: 835–844 17016424 10.1038/nrd2130

[emmm202114351-bib-0035] Wu J , Minikes AM , Gao M , Bian H , Li Y , Stockwell BR , Chen ZN , Jiang X (2019) Intercellular interaction dictates cancer cell ferroptosis via NF2‐YAP signalling. Nature 572: 402–406 31341276 10.1038/s41586-019-1426-6 PMC6697195

[emmm202114351-bib-0036] Yang WH , Ding CC , Sun T , Rupprecht G , Lin CC , Hsu D , Chi JT (2019) The hippo pathway effector TAZ regulates ferroptosis in renal cell carcinoma. Cell Rep 28: 2501–2508 e2504 31484063 10.1016/j.celrep.2019.07.107 PMC10440760

[emmm202114351-bib-0037] Yang WH , Huang Z , Wu J , Ding CC , Murphy SK , Chi JT (2020) A TAZ‐ANGPTL4‐NOX2 axis regulates ferroptotic cell death and chemoresistance in epithelial ovarian cancer. Mol Cancer Res 18: 79–90 31641008 10.1158/1541-7786.MCR-19-0691 PMC6942206

[emmm202114351-bib-0038] Yu G , He QY (2016) ReactomePA: an R/Bioconductor package for reactome pathway analysis and visualization. Mol Biosyst 12: 477–479 26661513 10.1039/c5mb00663e

[emmm202114351-bib-0039] Zheng J , Sato M , Mishima E , Sato H , Proneth B , Conrad M (2021) Sorafenib fails to trigger ferroptosis across a wide range of cancer cell lines. Cell Death Dis 12: 698 34257282 10.1038/s41419-021-03998-w PMC8277867

