# Supplementary material for: YAP/TAZ and ATF4 drive resistance to Sorafenib in hepatocellular carcinoma by preventing ferroptosis
Source: EMBO Mol Med. 2021 Oct 19;13(12):e14351. doi: 10.15252/emmm.202114351 (PMC8649869; doi:10.15252/emmm.202114351)
Supplement: Supplementary file 1 — Appendix [file EMMM-13-e14351-s014.pdf]

Appendix

YAP/TAZ and ATF4 drive resistance to Sorafenib therapy in hepatocellular carcinoma by preventing ferroptosis

Ruize Gao, Ravi K.R. Kalathur, Mairene Coto-Llerena, Caner Ercan, David Buechel, Song Shuang, Salvatore Piscuoglio, Michael T. Dill, Fernando D. Camargo, Gerhard Christofori, and Fengyuan Tang

Table of Contents

Appendix Figures S1 – S9 with their legends

Appendix Tables S1 - S3.

Appendix Figures

Appendix Figure S1. Gao et al.

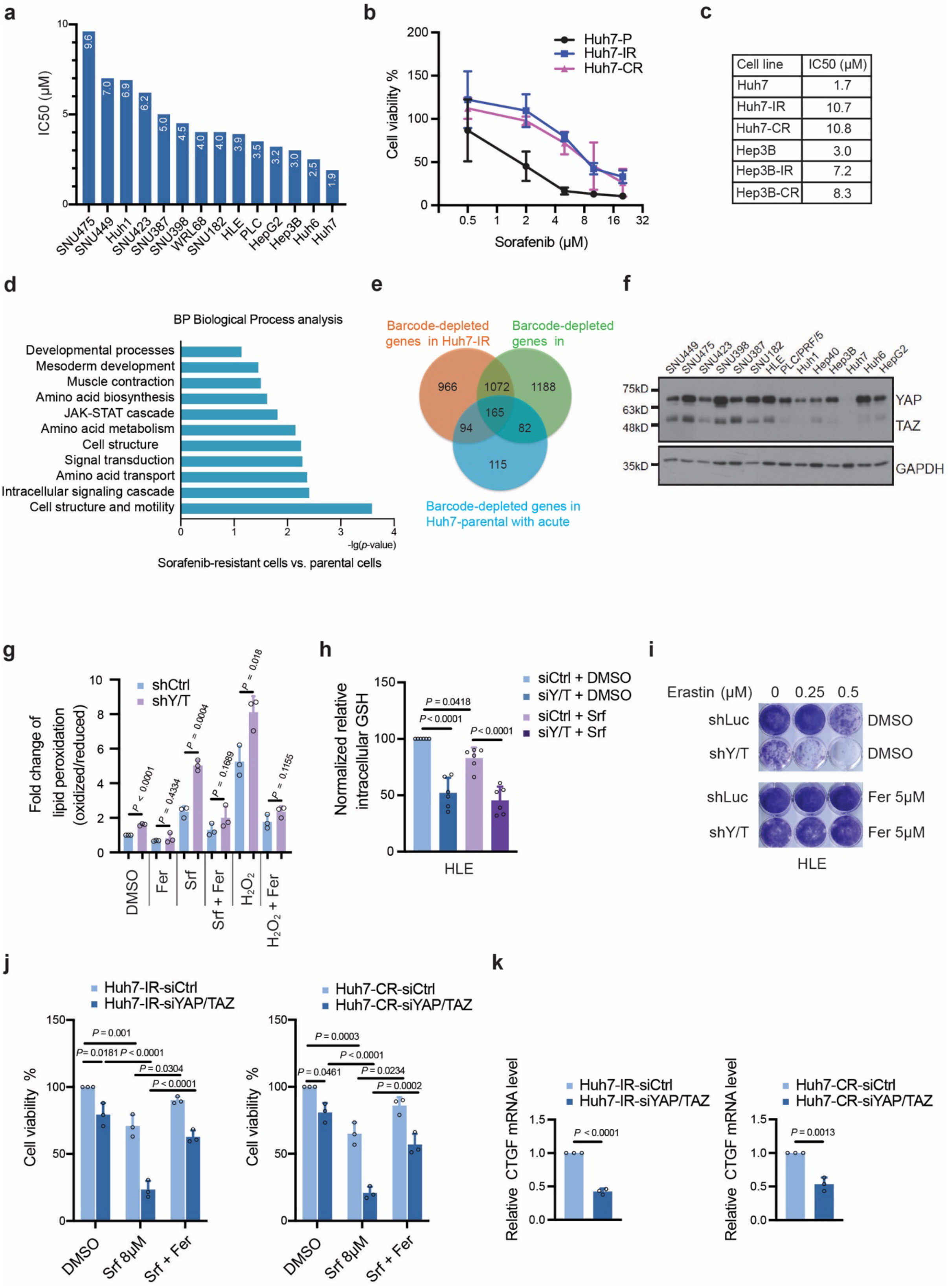

Appendix Figure S1. YAP/TAZ are key drivers of Sorafenib resistance through inhibition of lipid peroxidation.

(a) IC50 for Sorafenib responsiveness of different HCC cell lines. Different patient-derived HCC cell lines were treated with increasing doses of Sorafenib, and the IC50 values for cell growth inhibition by Sorafenib were determined. Hep3B and Huh7 were selected as two Sorafenib-responsive and HLE and SNU398 as moderate Sorafenib-resistant HCC cell lines (taken from Gao *et al*, 2021).

(b) Huh7-IR and Huh7-CR cells showing higher tolerance to Sorafenib than Huh7-parental cells. Cells were seeded into 96-well plate with 5000 cells/well, treated with increasing concentration of Sorafenib for 24 hours before harvest. Cell viability was measured using the Promega CellTiter-Glo 2.0 kit and normalized to 0μM Sorafenib. (N = 4)

(c) Sorafenib IC50 values of HUH7, Huh7-IR, Huh7-CR and Hep3B, Hep3B-IR and Hep3B-CR. These IC50 values are fairly close to Sorafenib's clinically relevant concentration of 10μM.

(d) Biological process analysis of the gene expression profiles of Sorafenib-resistant cells revealed a significant shift in amino acid metabolism, among other signaling pathways and regulatory networks.

(e) Overlapping barcode-depleted genes in Huh7-IR, Huh7-CR and Huh7-parental upon acute Sorafenib treatment. 1072 barcode-depleted genes were identified to maintain Sorafenib resistance after subtracting the 165 barcode-depleted genes of acute Sorafenib-treated Huh7-parental cells.

(f) YAP and TAZ protein expression in various HCC cell lines. Immunoblotting for YAP and TAZ in cell lysates of the HCC cell lines indicated. Immunoblotting for GAPDH was used as loading control.

(g) Lipid peroxidation levels increased upon depletion of YAP/TAZ, in particular in combination with Sorafenib (Srf) or H_2O_2. This increase was repressed by the ferroptosis inhibitor Ferrostatin-1 (Fer). HLE cells stably expressing luciferase (HLE-shLuc) or shRNA against YAP/TAZ (HLE-shY/T) were treated as indicated and stained with C11-BODIPY 581/591. Reduced-Bodipy was measured by flow cytometry with a 488 nm laser and oxidized-Bodipy was measured using a 561 nm laser. Ratios of oxidized/reduced represent the fold-change of lipid peroxidation. Data are shown as mean ± standard deviation (SD). Statistical significance was calculated using one-way ANOVA. Results represent three independent experiments.

(h) Intracellular GSH levels declined with the depletion of YAP/TAZ either with or without Sorafenib treatment. HLE-shLuc and HLE-shY/T cells were cultured with DMSO or 6μM Sorafenib for 18 hours, and intracellular GSH levels were measured using the GSH-Glo Glutathione Assay kit. Data are shown as mean ± standard deviation (SD). Statistical significance was calculated using one-way ANOVA. Results represent three independent experiments.

(i) Erastin-induced cell death of YAP/TAZ-depleted HCC cells was prevented by Ferrostatin-1. HLE-shLuc and HLE-shY/T cells were treated with different concentrations of Erastin (0, 0.25, 0.5μM) plus DMSO or Ferrostatin-1 (Fer; 5μM) for 2 weeks. Results represent three independent experiments.

(j) Cell viability assay showing that the knockdown of YAP/TAZ in Sorafenib-resistant Huh7-IR cells (left panel) and Huh7-CR cells (right panel) induced higher rates of cell death in response to Sorafenib (Srf; 8μM) treatment which could be overcome by treatment with Ferrostatin-1 (Fer; 5μM) 12 hours before harvest. Cell viability was measured with Promega CellTiter-Glo 2.0 kit and normalized to siCtrl-DMSO. Data are shown as mean ± standard deviation (SD). Statistical significance was calculated using two-way ANOVA. Results represent three independent experiments.

(k) Knockdown efficiency of siYAP/TAZ was assessed by quantitative RT-PCR analysis. RNA was extracted from the cells described in (i) and analyzed by quantitative RT-PCR. *CTGF* as a direct transcriptional target of YAP/TAZ served as positive control to confirm the knockdown efficiency of siYAP/TAZ. Data are shown as mean ± standard deviation (SD). Statistical significance was calculated using unpaired t-test. Results represent three independent experiments.

Appendix Figure S2. Gao et al.

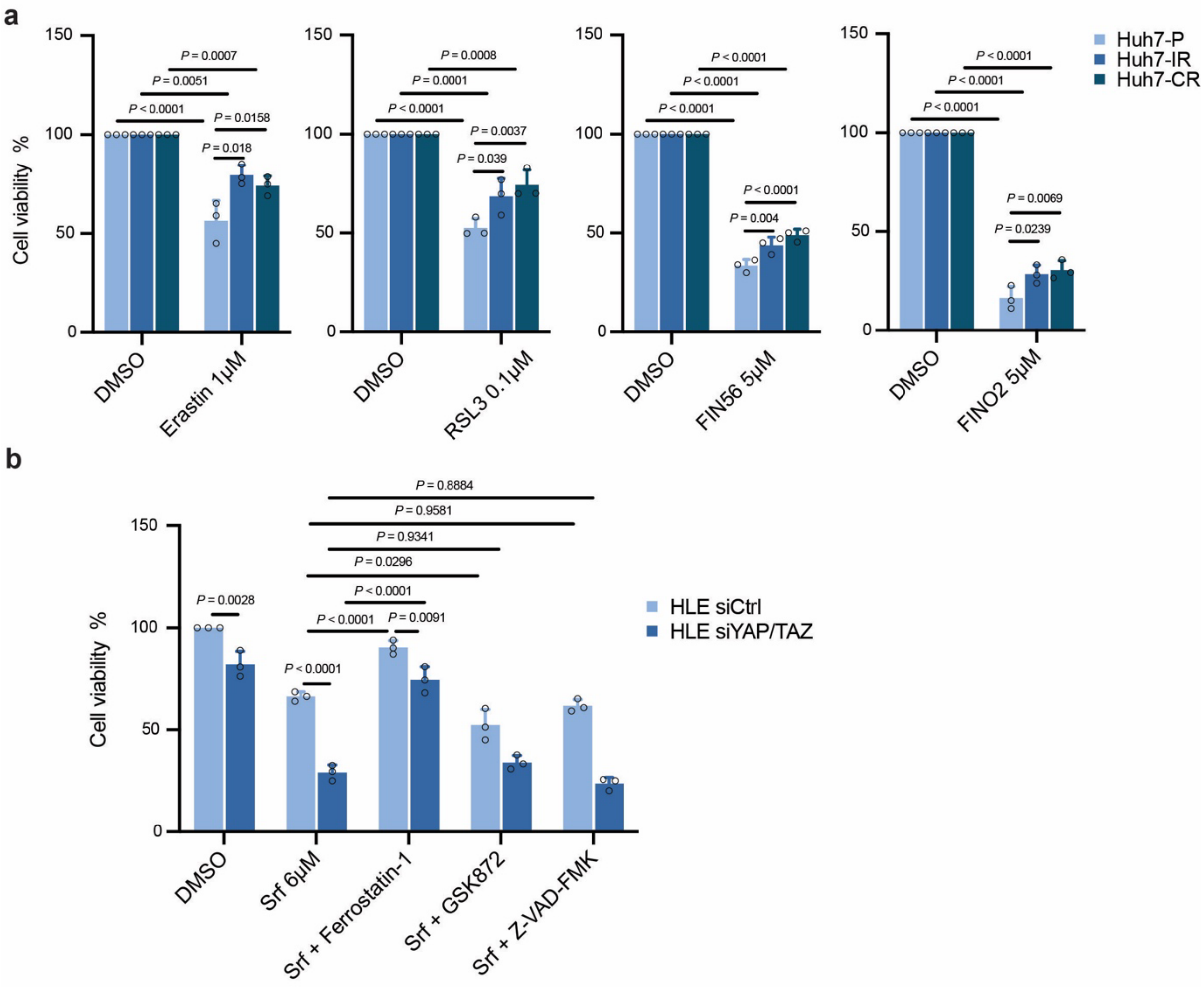

Appendix Figure S2. Ferroptosis is repressed in Sorafenib-resistant HCC cells.

(a) Cell viability assay showing that Sorafenib-resistant Huh7 cells are resistant to various ferroptosis inducers, including Erastin, RSL3, FIN56, and FINO2. Cells were treated with either 1μM Erastin for 24 hours, 0.1μM RSL3 for 12 hours, 5μM FIN56 for 18 hours, or 5μM FINO2 for 18 hours. Cell viability was measured using the Promega CellTiter-Glo 2.0 kit and normalized to the respective DMSO treatments. Data are shown as mean ± standard deviation (SD). Statistical significance was calculated using two-way ANOVA. Results represent three independent experiments.

(b) YAP/TAZ-deficiency induced death of HLE cells in response to Sorafenib treatment could be rescued by Ferrostatin-1 but not by GSK872 or Z-VAD-FMK. HLE cells transfected with either siCtrl or siYAP/TAZ were treated with 6μM Sorafenib (Srf) with or without 5μM Ferrostatin-1, 10μM GSK872 or 10μM Z-VAD-FMK for 20 hours before harvest. Cell

viability was measured using Promega CellTiter-Glo 2.0 kit and normalized to siCtrl-DMSO. Data are shown as mean ± standard deviation (SD). Statistical significance was calculated using two-way ANOVA. Results represent three independent experiments.

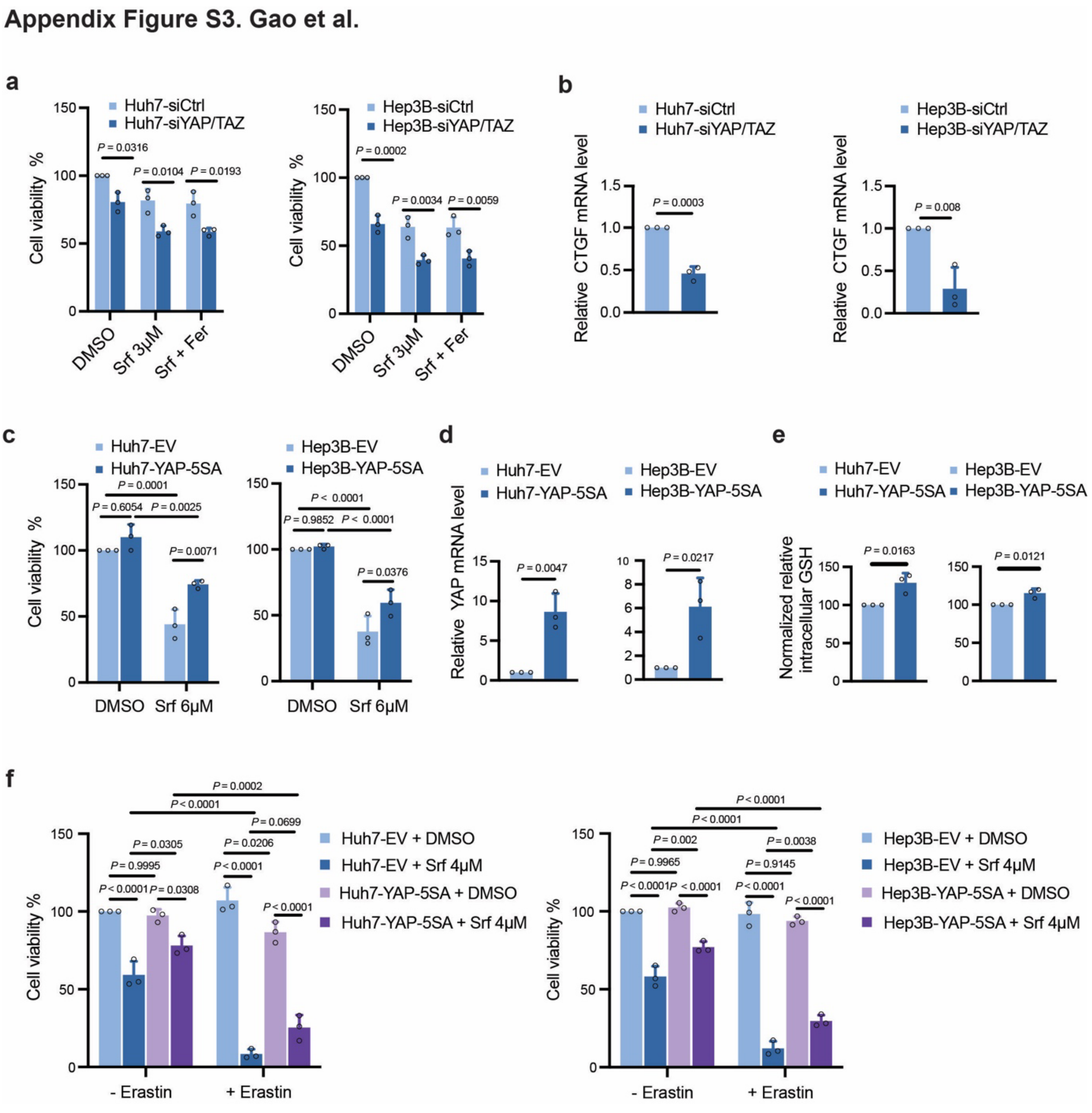

Appendix Figure S3. YAP/TAZ promote resistance to ferroptosis in Sorafenib-sensitive cells.

(a) Cell viability assay showing that the loss of YAP/TAZ increased death of Sorafenib-sensitive (parental) Huh7 and Hep3B cells in response to Sorafenib treatment which could not

be reversed by treatment with Ferrostatin-1. Huh7 and Hep3B cells were transfected with siYAP/TAZ and then treated with 3μM Sorafenib (Srf) with or without 5μM Ferrostatin-1 (Fer) for 12 hours before harvest. Cell viability was measured with Promega CellTiter-Glo 2.0 kit and normalized to siCtrl-DMSO. Data are shown as mean ± standard deviation (SD). Statistical significance was calculated using two-way ANOVA. Results represent three independent experiments.

(b) Quantitative RT-PCR analysis confirmed the knockdown efficiency of YAP/TAZ as determined from the experiment shown above. RNA was extracted and analyzed by quantitative RT-PCR. Expression of *CTGF* as a direct transcriptional target gene of YAP/TAZ was used to assess the knockdown efficiency of siYAP/TAZ. Data are shown as mean ± standard deviation (SD). Statistical significance was calculated using unpaired t-test. Results represent three independent experiments.

(c) Activated YAP overexpression confers Sorafenib resistance in Huh7 and Hep3B cells. Huh7 and Hep3B cells transfected with empty vector (EV) or with a cDNA construct coding for YAP-5SA were cultured with either DMSO or 6μM Sorafenib (Srf) for 12 hours before harvest. Cell viability was measured with Promega CellTiter-Glo 2.0 kit and normalized to Huh7 and Hep3B transfected with empty vector (EV) and treated with DMSO solvent. Data are shown as mean ± standard deviation (SD). Statistical significance was calculated using two-way ANOVA. Results represent three independent experiments.

(d) Quantitative RT-PCR analysis confirmed the overexpression of activated YAP. RNA was extracted from the cells described in (c) and *YAP* mRNA expression was analyzed by quantitative RT-PCR. Data are shown as mean ± standard deviation (SD). Statistical significance was calculated using unpaired t-test. Results represent three independent experiments.

(e) Intracellular GSH levels increased with the overexpression of activated-YAP (YAP-5SA). The intracellular GSH levels were measured in Huh7 and Hep3B cells described in (c) using the GSH-Glo Glutathione Assay kit and normalized to Huh7-EV and Hep3B-EV, respectively. Data are shown as mean ± standard deviation (SD). Statistical significance was calculated using unpaired t-test. Results represent three independent experiments.

(f) Combination of Sorafenib and Erastin induced higher rates of cell death as compared to single Sorafenib treatments in activated-YAP overexpressing Huh7 and Hep3B cells. Empty vector control (EV) transfected and YAP-5SA-expressing Huh7 and Hep3B cells were treated with DMSO or 4μM Sorafenib, combined with or without 2μM Erastin treatment for 12 hours

before harvest. Cell viability was measured with Promega CellTiter-Glo 2.0 kit and normalized to empty vector-transfected Huh7/Hep3B treated with DMSO. Data are shown as mean ± standard deviation (SD). Statistical significance was calculated using Two-way ANOVA. Results represent three independent experiments.

Appendix Figure S4. Gao et al.

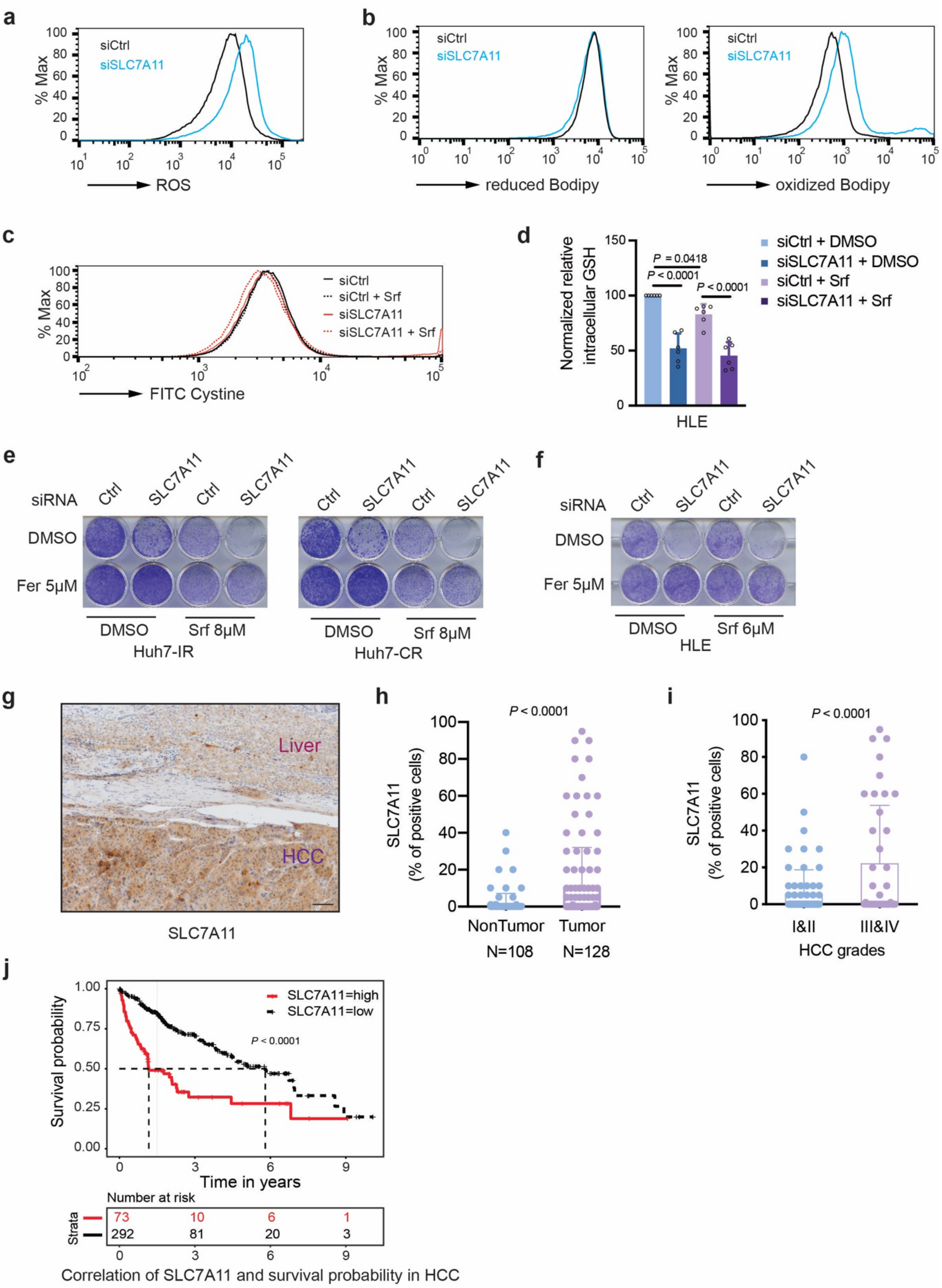

Appendix Figure S4. SLC7A11 is a regulator of ferroptosis in HCC cells.

(a) Levels of reactive oxygen (ROS) increased upon loss of SLC7A11. HLE cells were transfected with siCtrl or siSLC7A11, cultured for 36 hours, and stained with CellROX™ Green Flow Cytometry Assay Kit. ROS levels were measured by flow cytometry using a 488 nm laser. Results represent three independent experiments.

(b) Lipid peroxidation increased upon loss of SLC7A11. HLE cells were transfected with siCtrl or siSLC7A11, cultured for 36 hours, and stained with C11-BODIPY 581/591. Reduced-Bodipy was measured by flow cytometry using a 488 nm laser, and oxidized-Bodipy was measured with a 561 nm laser. A significant shift of oxidized-Bodipy occurred upon depletion of SLC7A11. Results represent three independent experiments.

(c) SLC7A11 deficiency impaired cystine uptake by HCC cells either with or without Sorafenib treatment. HLE cells were transfected with siCtrl or siSLC7A11 and treated for 18 hours with DMSO or 6μM Sorafenib. Cystine-FITC was added to the cells for 30 minutes at 37ºC, and intracellular Cystine-FITC levels were determined by flow cytometry with a 488 nm laser. Results represent three independent experiments.

(d) Intracellular GSH levels declined with the depletion of SLC7A11 either with or without Sorafenib treatment. HLE cells were transfected with siCtrl or siSLC7A11 and treated with DMSO or 6μM Sorafenib for 18 hours. Intracellular GSH levels were measured using the GSH-Glo Glutathione Assay kit. Data are shown as mean ± standard deviation (SD). Statistical significance was calculated using one-way ANOVA. Results represent six independent experiments.

(e) Colony formation assay demonstrating that the ferroptosis inhibitor Ferrostatin-1 reversed Sorafenib-induced cell death in SLC7A11-deficient HCC cells. Huh7-IR and Huh7-CR cells were transfected with siCtrl or siSLC7A11 and treated with Sorafenib (8μM) or DMSO plus either DMSO or Ferrostatin-1 (Fer; 5μM) for 2 weeks. Results represent three independent experiments.

(f) Colony formation assay showing that the ferroptosis inhibitor Ferrostatin-1 reversed Sorafenib-induced cell death in SLC7A11-deficient HCC cells. HLE cells transfected with siCtrl or siSLC7A11 were treated with Sorafenib (6μM) or DMSO plus either DMSO or Ferrostatin-1 (Fer; 5μM) for 2 weeks. Results represent three independent experiments.

(g) Immunohistochemical staining of SLC7A11 in HCC and adjacent non-neoplastic areas from patients. Tumor tissues showed a higher expression of SLC7A11. Scale bar, 100μm.

(h) Quantification of SLC7A11-positive cells in tumor and non-tumor samples showed that HCC tumors present higher SLC7A11 levels. Data are shown as mean ± standard deviation (SD). Statistical significance was calculated using unpaired t-test.

(i) SLC7A11 protein correlated with aggressiveness of HCC samples as assessed by Edmondson grade III and IV samples. HCC grade I & II (N=75), HCC grade III & IV (N=40). Data are shown as mean ± standard deviation (SD). Statistical significance was calculated using unpaired t-test.

(j) Kaplan-Meyer analysis of the TCGA database revealed that high SLC7A11 expression in HCC of patients significantly correlated with poor clinical outcome. Statistical significance was calculated using log-rank (Mantel-Cox) test.

Appendix Figure S5. Gao et al.

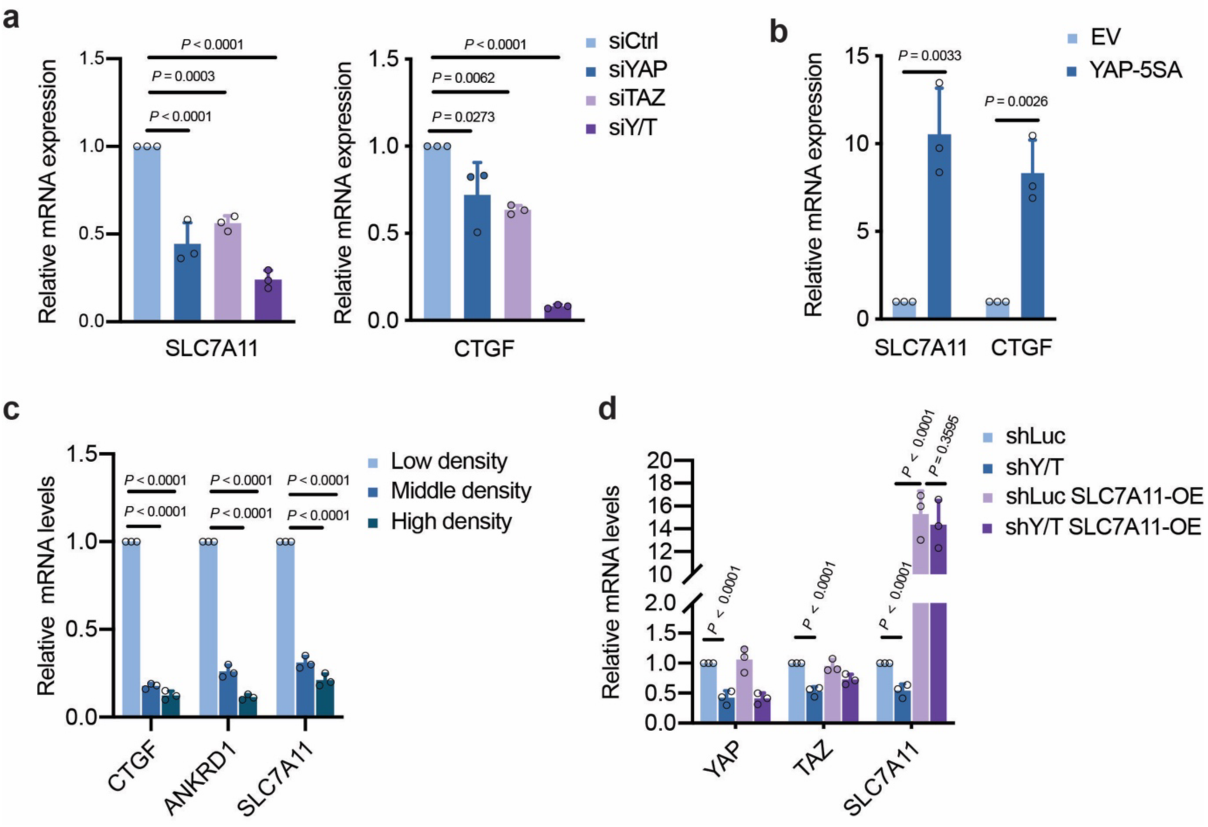

Appendix Figure S5. YAP/TAZ transcriptionally upregulate *SLC7A11* expression.

(a) Either YAP or TAZ deficiency, and most effectively their combinatorial depletion, reduced *SLC7A11* mRNA levels. HLE cells were transfected with siCtrl, siYAP, siTAZ or siY/T as indicated, and mRNA levels were determined by quantitative RT-PCR. The YAP/TAZ

transcriptional target *CTGF* served as positive control. Data are shown as mean ± standard deviation (SD). Statistical significance was calculated using one-way ANOVA. Results represent three independent experiments.

(b) The forced expression of a constitutive-active form of YAP (YAP-5SA) upregulated SLC7A11 expression. HLE cells were transfected with empty vector (EV) or a vector coding for YAP-5SA, and *SCL7A11* mRNA levels were determined by quantitative RT-PCR. The YAP/TAZ transcriptional target gene *CTGF* served as positive control. Data are shown as mean ± standard deviation (SD). Statistical significance was calculated using one-way ANOVA. Results represent three independent experiments.

(c) High cell density reduced *SLC7A11* mRNA levels. HLE cells were seeded at different cell numbers to obtain low, medium and high cell densities. *SCL7A11* mRNA levels were determined by quantitative RT-PCR. *CTGF* and *ANKRD1* served as positive controls for YAP/TAZ-dependent transcriptional activity. Data are shown as mean ± standard deviation (SD). Statistical significance was calculated using one-way ANOVA. Results represent three independent experiments.

(d) Validation of shRNA-mediated depletion of YAP and TAZ expression and of the forced expression of SLC7A11. The efficiency of shRNA-mediated knockdown of YAP/TAZ (shY/T) and of the forced expression of *SLC7A11* (SLC7A11-OE) was determined by quantitative RT-PCR. Data are shown as mean ± standard deviation (SD). Statistical significance was calculated using one-way ANOVA. Results represent three independent experiments.

Appendix Figure S6. Gao et al.

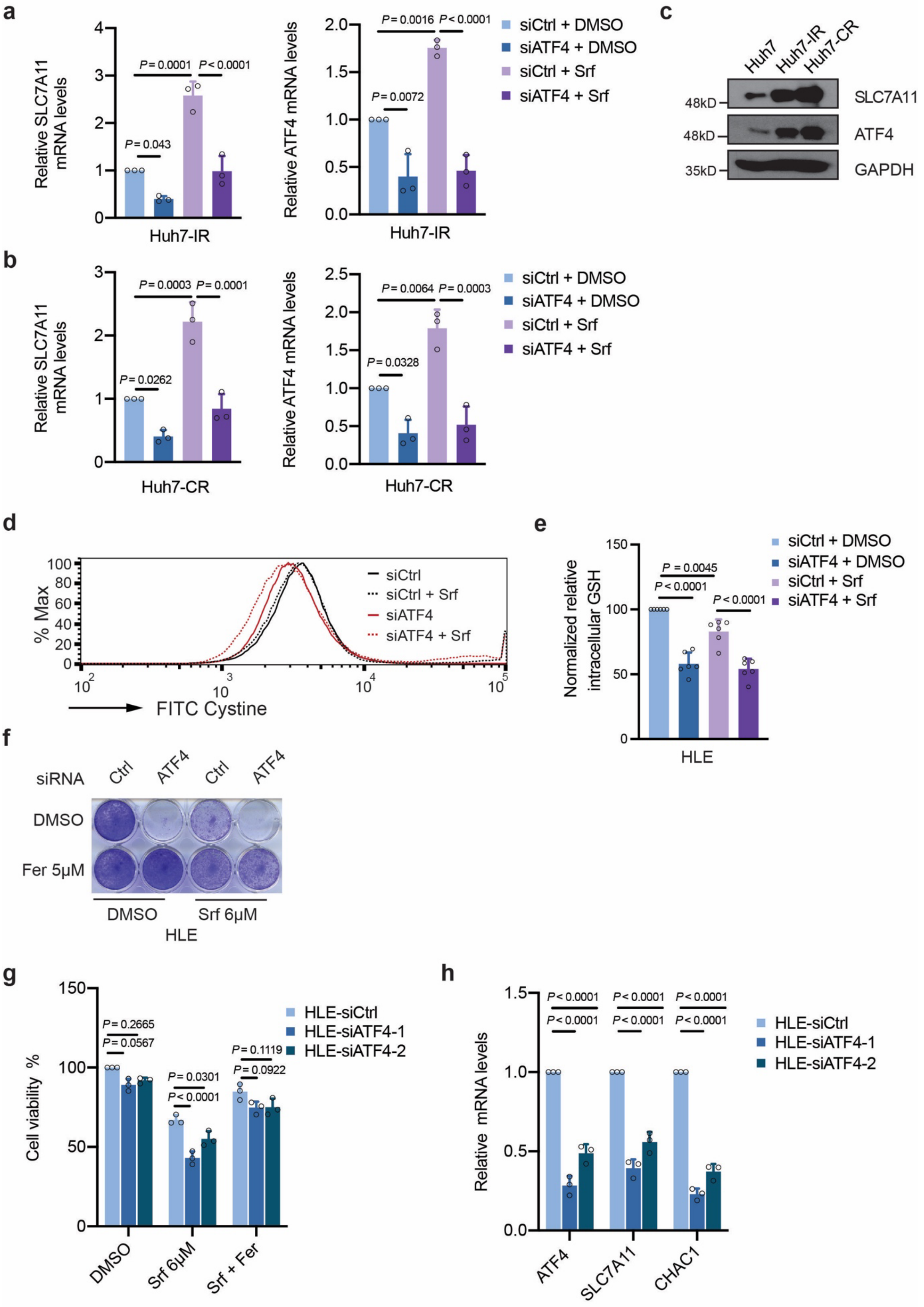

Appendix Figure S6. ATF4 regulates ferroptosis.

(a,b) *SLC7A11* mRNA levels were decreased upon ATF4 depletion in Sorafenib-resistant Huh7-IR and Huuh7-CR cells. Huh7-IR cells (a) and Huh7-CR cells (b) were transfected with siCtrl or siATF4 and cultured with DMSO or 6μM Sorafenib for 18 hours. Quantitative RT-PCR was used to determine *SLC7A11* (left) and *ATF4* (right) mRNA levels. *SLC7A11* decreased with siATF4 either under DMSO or Sorafenib treatment. Knockdown efficiency of siATF4 was determined by quantitative RT-PCR for ATF4 expression. Data are shown as mean ± standard deviation (SD). Statistical significance was calculated using one-way ANOVA. Results represent three independent experiments.

(c) Immunoblotting analysis of Sorafenib-resistant Huh7-IR and Huh7-CR cells revealed higher protein levels of ATF4 and SLC7A11 as compared to Sorafenib-sensitive parental Huh7 cells. GAPDH served as loading control. Results represent three independent experiments.

(d) Depletion of ATF4 impaired cystine uptake in the presence or absence of Sorafenib. HLE cells transfected with siCtrl or siATF4 were treated with DMSO or 6μM Sorafenib for 18 hours. FITC-Cystine was added to the for 30 minutes at 37ºC, and the intracellular FITC-Cystine levels were determined by flow cytometry with a 488 nm laser. Results represent three independent experiments.

(e) Intracellular GSH levels declined with the depletion of ATF4 either with or without Sorafenib treatment. HLE cells transfected with siCtrl or siATF4 were treated with DMSO or 6μM Sorafenib for 18 hours, and intracellular GSH levels were measured using the GSH-Glo Glutathione Assay kit. Data are shown as mean ± standard deviation (SD). Statistical significance was calculated using one-way ANOVA. Results represent six independent experiments.

(f) Colony formation assay showing that the ferroptosis inhibitor Ferrostatin-1 could reverse Sorafenib-induced cell death in ATF4-deficient HCC cells. HLE cells transfected with siCtrl or siATF4 were treated with Sorafenib (6μM) or DMSO plus either DMSO or Ferrostatin-1 (Fer; 5μM) for 2 weeks. Results represent three independent experiments.

(g) ATF4 deficiency induced cell death in response to Sorafenib which was overcome by treatment with Ferrostatin-1 (Fer). HLE cells were transfected with siCtrl, siATF4-1 or siATF4-2 and treated with 6μM Sorafenib with or without 5μM Ferrostatin-1 for 12 hours before harvest. Cell viability was measured with Promega CellTiter-Glo 2.0 kit and normalized to HLE-siCtrl DMSO. Data are shown as mean ± standard deviation (SD). Statistical

significance was calculated using two-way ANOVA. Results represent three independent experiments.

(h) Quantitative RT-PCR analysis confirmed the knockdown efficiency of siATF4-1 and siATF4-2. RNA from the cells described in (g) was extracted and analyzed by quantitative RT-PCR. mRNA levels of *SLC7A11* and *CHAC1* decreased significantly with the deficiency of ATF4. Data are shown as mean ± standard deviation (SD). Statistical significance was calculated using two-way ANOVA. Results represent three independent experiments.

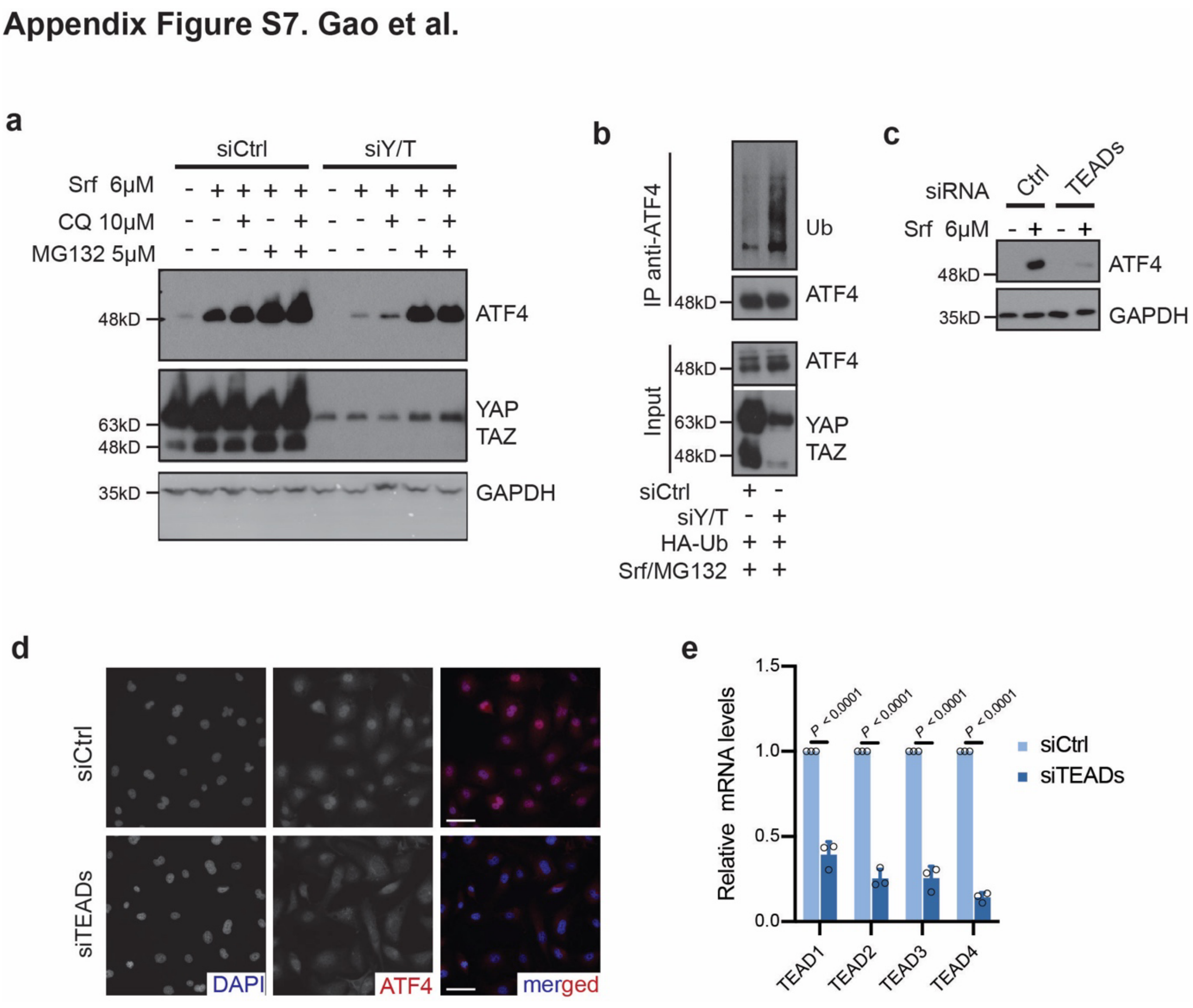

Appendix Figure S7. YAP/TAZ and TEADs stabilize ATF4 protein.

(a) YAP/TAZ protect ATF4 from proteasome-mediated degradation. HLE cells were transfected with siCtrl or siYAP/TAZ (siY/T) and treated with Sorafenib and the autophagy inhibitor chloroquine (CQ) or the proteasome inhibitor MG132 for 18 hours, as indicated.

Immunoblotting showed that YAP/TAZ deficiency declined Sorafenib-induced ATF4 protein levels which was blocked by MG132 but not by CQ. GAPDH served as loading control. Results represent three independent experiments.

(b) YAP/TAZ deficiency promoted ubiquitylation of ATF4. HLE cells were treated with Sorafenib and MG132 for 18 hours, before ATF4 was immunoprecipitated, and the immunoprecipitates were analyzed by immunoblotting for ATF4 and ubiquitylation of ATF4 (Ub) and for ATF4 and YAP/TAZ in the input samples. Results represent three independent experiments.

(c) TEAD deficiency reduced ATF4 expression. HLE cells were transfected with siCtrl or siTEADs (siTEAD1 + siTEAD2 + siTEAD3 + siTEAD4) and treated with 6μM Sorafenib. Immunoblotting visualized the levels of ATF4. GAPDH served as loading control. Results represent three independent experiments.

(d) TEADs direct the nuclear localization of ATF4. HLE cell were transfected with siCtrl or siTEADs, and the nuclear localization of ATF4 was analyzed by immunofluorescence microscopy. DAPI visualized nuclei. Scale bars, 50μm. Results represent three independent experiments.

(e) Validation of the knockdown efficiency of siTEAD-transfected HLE cells as determined by quantitative RT-PCR for *TEAD1*, *TEAD2*, *TEAD3* and *TEAD4* mRNA levels. Data are shown as mean ± standard deviation (SD). Statistical significance was calculated using one-way ANOVA. Results represent three independent experiments.

Appendix Figure S8. Gao et al.

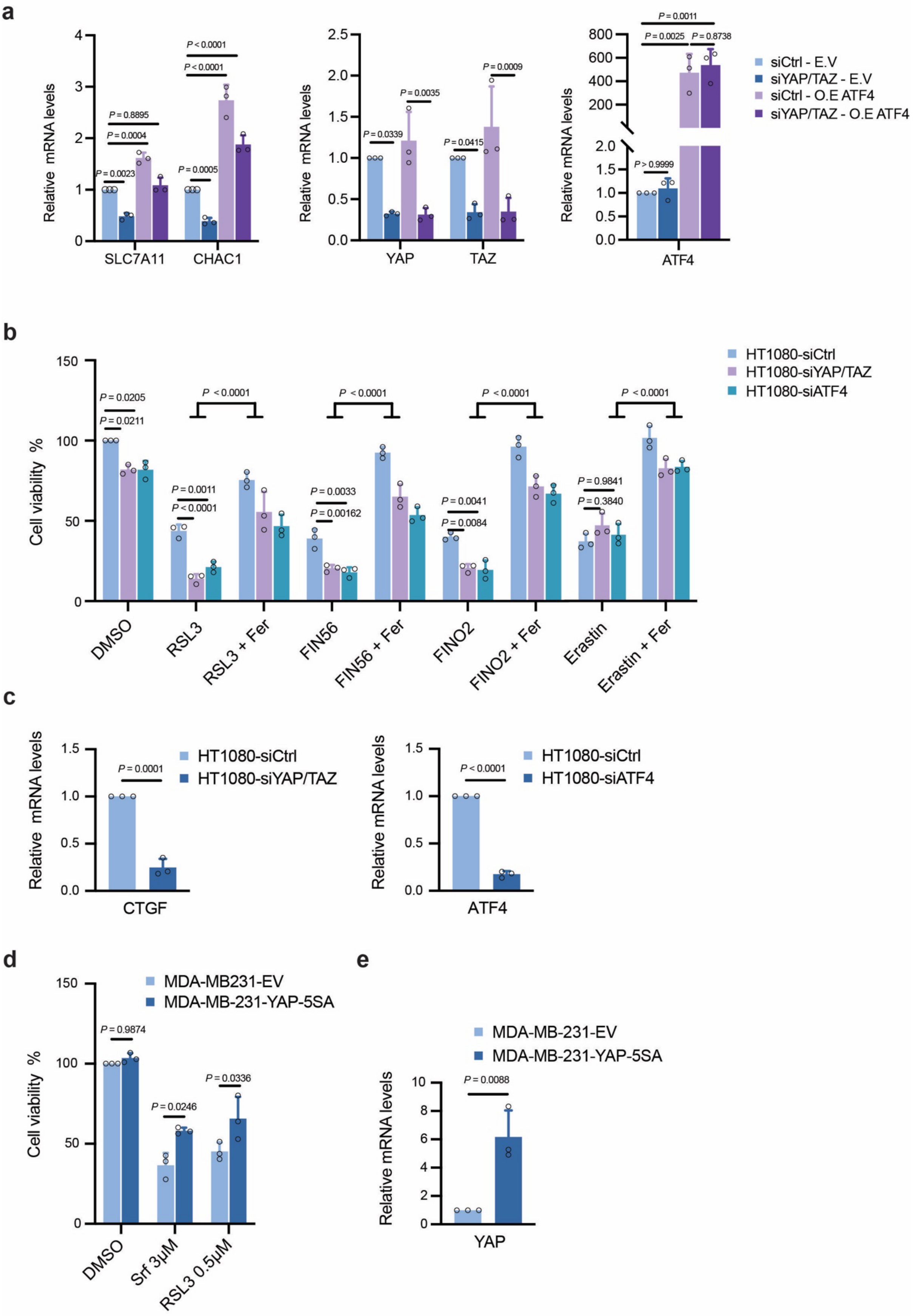

Appendix Figure S8. YAP/TAZ and ATF4 also prevent ferroptosis in other cancer cell types.

(a) Quantitative RT-PCR analysis demonstrated that YAP/TAZ deficiency reduced ATF4-induced upregulation of *SLC7A11* and *CHAC1* gene expression. HLE cells were transfected with siCtrl or siYAP/TAZ, and 24 hours later transfected with empty vector or a plasmid construct encoding *ATF4*. RNA was extracted and analyzed by quantitative RT-PCR. *SLC7A11* and *CHAC1* gene expression significantly increased with overexpression of ATF4, but decreased significantly upon knockdown of YAP/TAZ (left). Knockdown efficiency of YAP/TAZ and overexpression of ATF4 were confirmed by quantitative RT-PCR (middle and right). Data are shown as mean ± standard deviation (SD). Statistical significance was calculated using two-way ANOVA. Results represent three independent experiments.

(b) Cell viability assay showing that upon either YAP/TAZ or ATF4 deficiency in HT1080 cells treatments with RSL3, FIN56, or FINO2 resulted in increased cell death which could be overcome by treatment with Ferrostatin-1. In contrast, treatment with Erastin induced comparable rates of ferroptosis in the presence or absence of YAP/TAZ or ATF4. HT1080 cells were transfected with either siYAP/TAZ or siATF4 and 24 hours later treated with 0.3μM RSL3, 1μM FIN56, or 0.5μM FINO2 with or without 5μM Ferrostatin-1 for 20 hours before harvest. Cell viability was measured using Promega CellTiter-Glo 2.0 kit and normalized to siCtrl-DMSO. Data are shown as mean ± standard deviation (SD). Statistical significance was calculated using two-way ANOVA. Results represent 3 independent experiments.

(c) Quantitative RT-PCR analysis confirmed the knockdown efficiency of YAP/TAZ and ATF4 in HT1080 described in (b). *CTGF* as a direct transcriptional target gene of YAP/TAZ served as positive control to confirm the knockdown efficiency of siYAP/TAZ. Data are shown as mean ± standard deviation (SD). Statistical significance was calculated using unpaired t-test. Results represent three independent experiments.

(d) Activated YAP overexpression confers resistance to Sorafenib and RSL3 in MDA-MB-231 cells. MDA-MB-231 (empty vector control (EV) or YAP-5SA-expressing cells were treated with either DMSO or 3μM Sorafenib or 0.5μM RSL3 for 12 hours before harvest. Cell viability was measured with Promega CellTiter-Glo 2.0 kit and normalized to MDA-MB-23-EV + DMSO. Data are shown as mean ± standard deviation (SD). Statistical significance was calculated using two-way ANOVA. Results represent three independent experiments.

(e) Quantitative RT-PCR analysis confirmed the overexpression of activated YAP in MDA-MB-231 cells described in (d). Data are shown as mean ± standard deviation (SD). Statistical significance was calculated using unpaired t-test. Results represent three independent experiments.

Appendix Figure S9 . Gao et al.

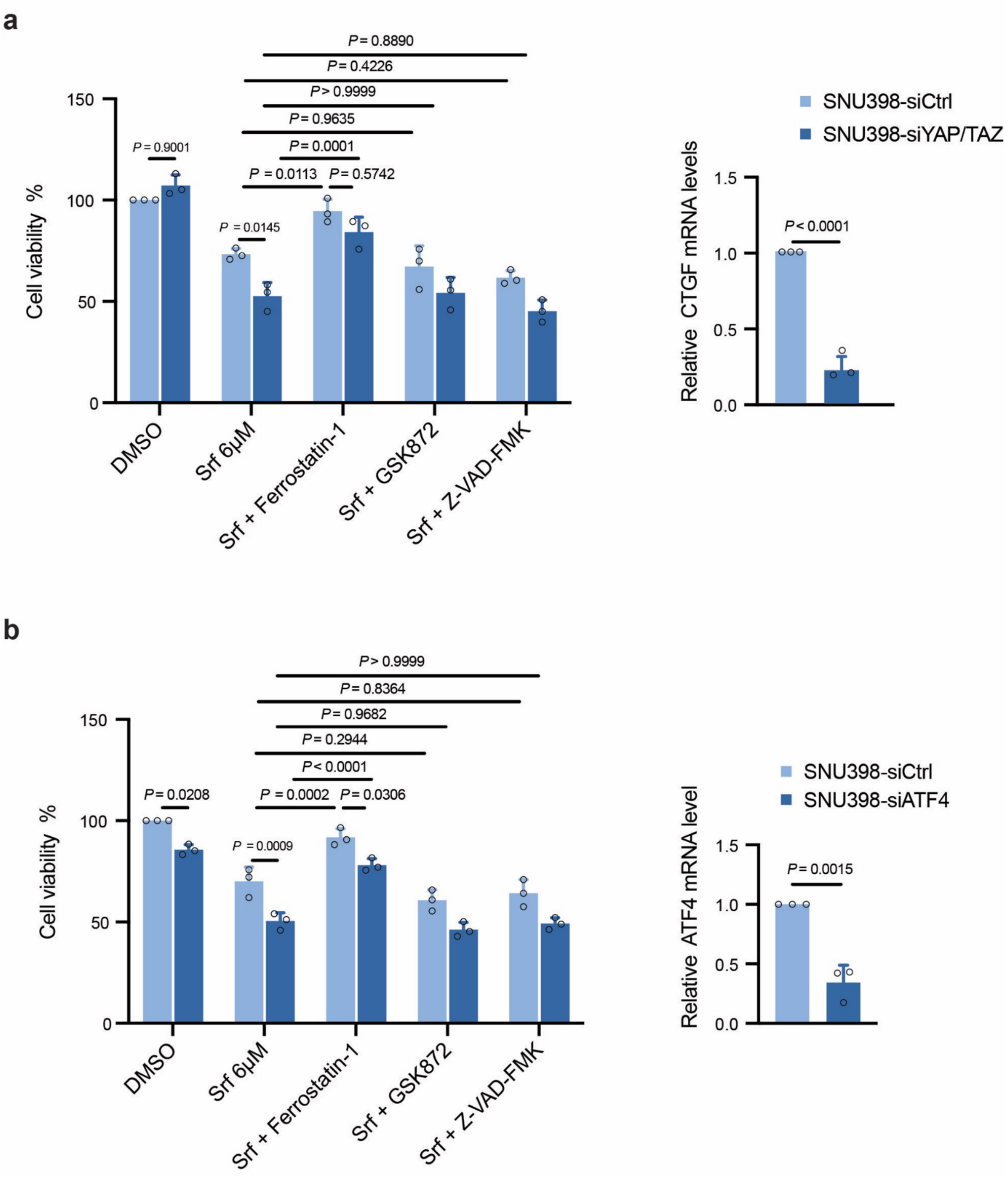

Appendix Figure S9. YAP/TAZ and ATF4 also prevent ferroptosis in highly tumorigenic SNU398 cells.

(a, b) Deficiency of YAP/TAZ induced higher rates of cell death in response to Sorafenib treatment in tumorigenic SNU398 cells, which could be rescued by Ferrostatin-1 but not by GSK872 or Z-VAD-FMK. SNU398 cells were transfected with either siCtrl or siYAP/TAZ and 24 hours later treated with 6μM Sorafenib with 5μM Ferrostatin-1, 10μM GSK872 or 10μM Z-VAD-FMK for 20 hours. Cell viability was measured with Promega CellTiter-Glo 2.0 kit and normalized to siCtrl-DMSO. Knockdown efficiencies of siYAP/TAZ and of siATF4 were determined by the mRNA expression levels of *CTGF*, a direct transcriptional target gene of YAP/TAZ, and of *ATF4* (right panels). Data are shown as mean ± standard deviation (SD). Statistical significance was calculated using two-way ANOVA. Results represent three independent experiments.

Appendix Tables

Appendix Table S1. siRNAs used in the study.

	Catalog ID (Horizon discovery)
siCtrl	ON-TARGET plus non-targeting pool (D-001810-10-20)
siYAP1	ON-TARGET plus Human YAP1 (L-012200-00-0005)
siTAZ	ON-TARGET plus Human TAZ (L-009608-00-0005)
siATF4	ON-TARGET plus Human ATF4 (L-005125-00-0005)
siATF4-1	ON-TARGET plus Human ATF4 (J-005125-10-0002)
siATF4-2	ON-TARGET plus Human ATF4 (J-005125-11-0002)
siSLC7A11	ON-TARGET plus Human SLC7A11 (L-007612-01-0005)
siNRF2	ON-TARGET plus Human NFE2L2 siRNA (L-003755-00-0005)

Appendix Table S2. Antibodies used in the study.

Antibodies	Dilution	SOURCE	IDENTIFIER
YAP	1:1000	CST	4912
YAP	1:1000	Abcam	ab52771
TAZ(V386)	1:1000	CST	4883

YAP/TAZ (63.7)	1:1000	Santa Cruz	Sc-101199
GAPDH	1:5000	Abcam	ab9485
ATF-4 (D4B8)	1:2000	CST	11815
ATF-4	1:2000	Abcam	ab221791
SLC7A11 (D2M7A)	1:1000	CST	12691S
4-HNE	1:200	Abcam	ab46545
NRF2 (EP1808Y)	1:1000	Abcam	ab62352
Lamin A/C	1:1000	Santa Cruz	Sc-6215
Ki67	1:200	Abcam	ab833
Ubiquitin	1:1000	CST	3933

Appendix Table S3. PCR primers used in the study.

Oligonucleotides	5' --- 3'
hRPL19, forward primer	GATGCCGGAAAAACACCTTG
hPRL19, reverse primer	CAGGGCAGTGATCTCCTTCTG
YAP, forward primer	CCTTCTTCAAGCCGCCGGAG
YAP, reverse primer	CAGTGTCCCAGGAGAAACAGC
TAZ, forward primer	TATCCCAGCCAAATCTCGTG
TAZ, reverse primer	TTCTGCTGGCTCAGGGTACT
ATF4, forward primer	CCCTTCACCTTCTTACAACCTC
ATF4, reverse primer	TGCCCAGCTCTAAACTAAAGGA
CTGF, forward primer	AGGAGTGGGTGTGTGACGA
CTGF, reverse primer	CCAGGCAGTTGGCTCTAATC
CYR61, forward primer	AGCCTCGCATCCTATACAACC
CYR61, reverse primer	TTCTTTCACAAGGCGGCACTC
SLC7A11, forward primer	TCTCCAAAGGAGGTTACCTGC
SLC7A11, reverse primer	AGACTCCCCTCAGTAAAGTGAC
ANKRD1, forward primer	AGTAGAGGAACTGGTCACTGG
ANKRD1, reverse primer	TGGGCTAGAAGTGTCTTCAGA
CHAC1, forward primer	GTGGTGACGCTCCTTGAAGATC
CHAC1, reverse primer	GAAGGTGACCTCCTTGGTATCG
NRF2, forward primer	CACATCCAGTCAGAAACCAGTGG
NRF2, reverse primer	GGAATGTCTGCGCCAAAAGCTG

ACLS4, forward primer	GCTATCTCCTCAGACACACCGA
ACSL4, reverse primer	AGGTGCTCCAACTCTGCCAGTA
GPX4, forward primer	GAGGCAAGACCGAAGTAAACTAC
GPX4, reverse primer	CCGAACTGGTTACACGGGAA
AIFM2, forward primer	GACTCCTTCCACCACAATGTGG
AIFM2, reverse primer	CAGCACCATCTGGTTCTTCAGG
ZEB1, forward primer	GGCATACACCTACTCAACTACGG
ZEB1, reverse primer	TGGGCGGTGTAGAATCAGAGTC
CYR61-CHIP-F	CCCTTGGCTGTTATGAGGAA
CYR61-CHIP-R	CCTTGCATTCCTTTGCATTT
SLC7A11-AARE-CHIP-F	TTGAGCAACAAGCTCCTCCT
SLC7A11-AARE-CHIP -R	CAAACCAGCTCAGCTTCCTC
SLC7A11-CHIP-P1-F	GTAGCTTTAGGATACATTCTACTCACA
SLC7A11-CHIP-P1-R	GCAACTCGTAGTGAGCAACA
SLC7A11-CHIP-P2-F	TTGGATTTGACTGTATTGCCTT
SLC7A11-CHIP-P2-R	TTGTGAGTAGAATGTATCCTAAAGC
Negative control Chr10 (NC10)-F	ACCAACACTCTTCCCTCAGC
Negative control Chr10 (NC10)-R	TTATTTTGGTTCAGGTGGTTGA
