# Supplementary figures and images for: YAP/TAZ and ATF4 drive resistance to Sorafenib in hepatocellular carcinoma by preventing ferroptosis
Source: EMBO Mol Med. 2021 Oct 19;13(12):e14351. doi: 10.15252/emmm.202114351 (PMC8649869; doi:10.15252/emmm.202114351)

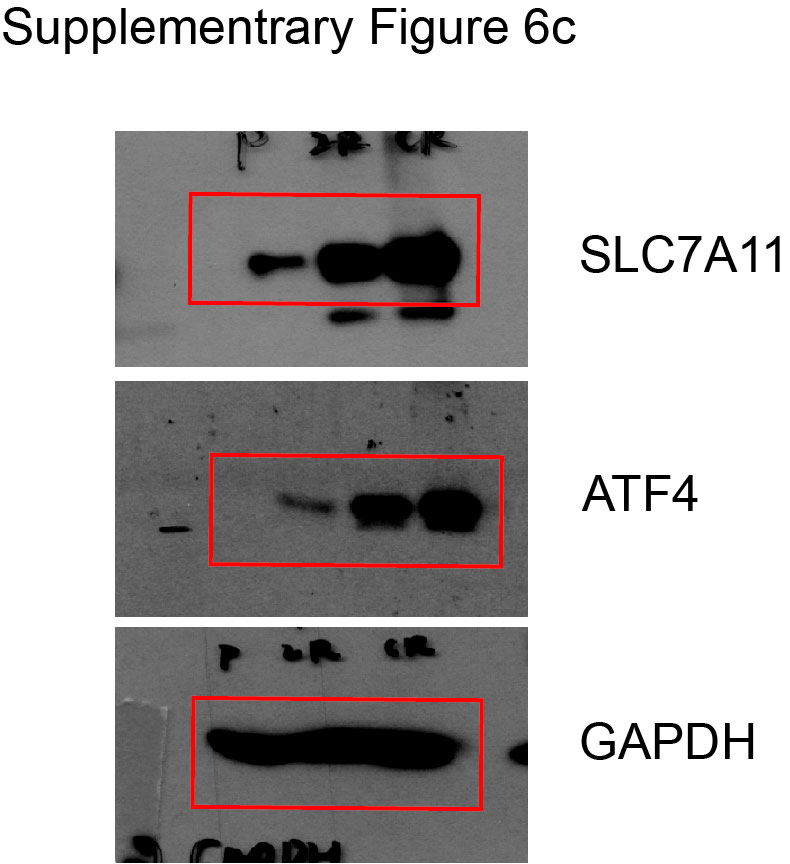

Supplement: Supplementary file 7 — Source Data for Appendix [file EMMM-13-e14351-s002.zip › EMM-2021-14351-V3-Appendix_Figure_Source_Data/EMM-2021-14351-V3-Appendix_Figure_6_Source_Data-sd.jpg]

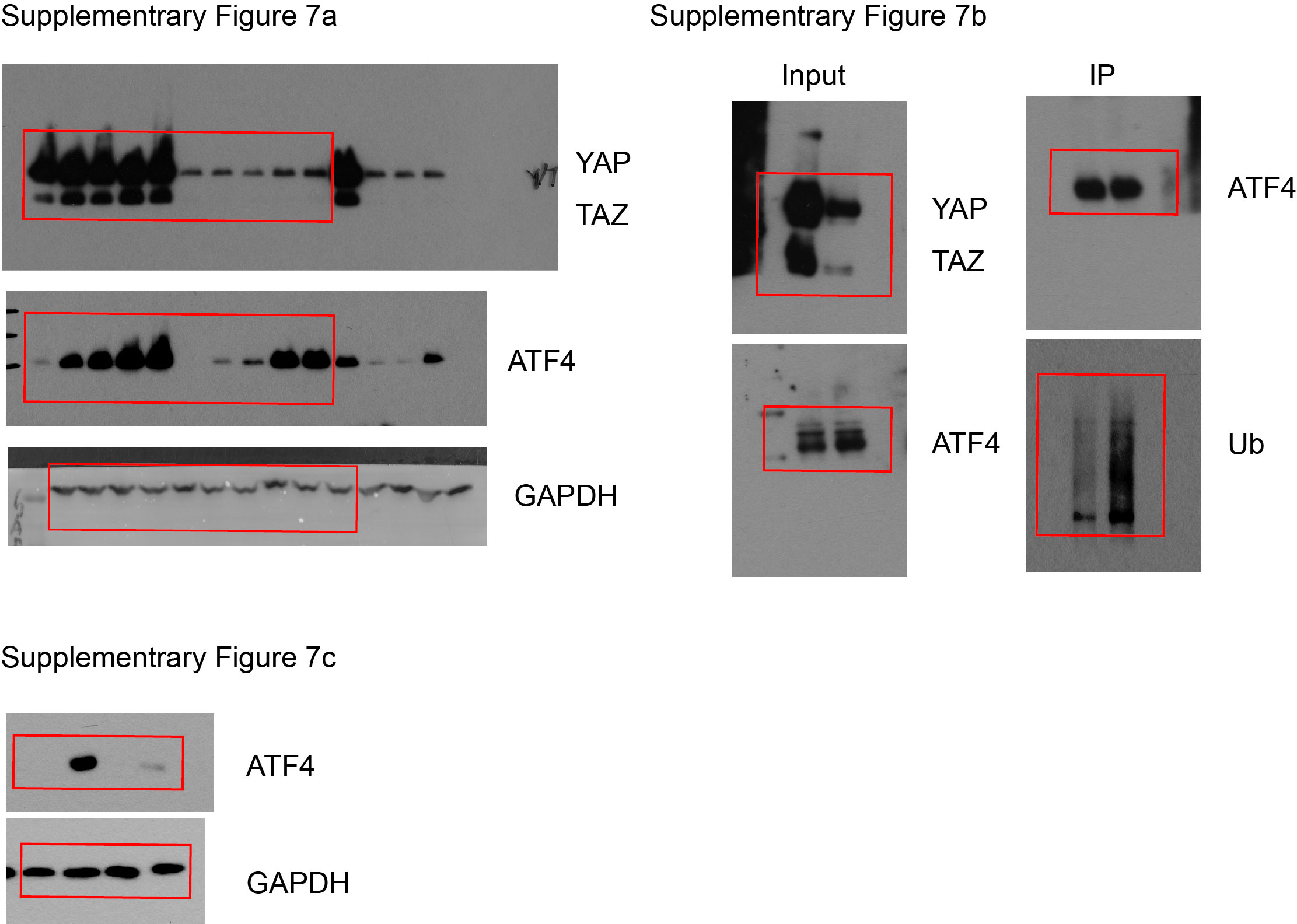

Supplement: Supplementary file 7 — Source Data for Appendix [file EMMM-13-e14351-s002.zip › EMM-2021-14351-V3-Appendix_Figure_Source_Data/EMM-2021-14351-V3-Appendix_Figure_7_Source_Data-sd.jpg]

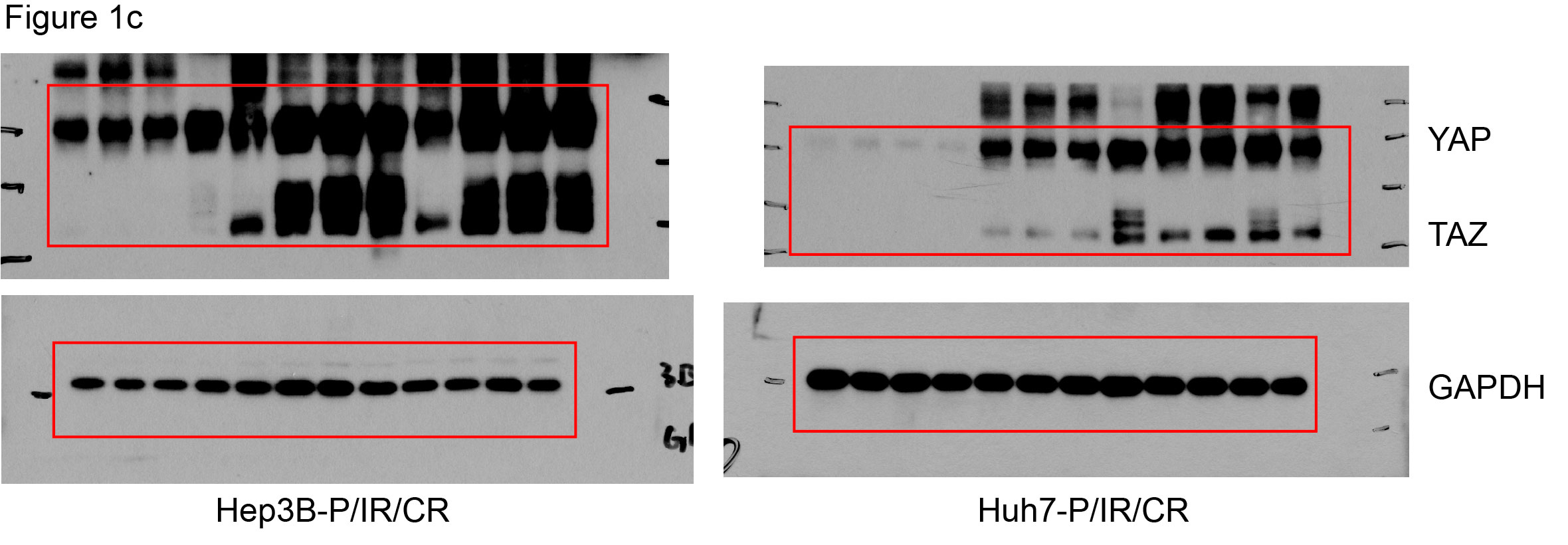

Supplement: Supplementary file 8 — Source Data for Figure 1 [file EMMM-13-e14351-s010.jpg]

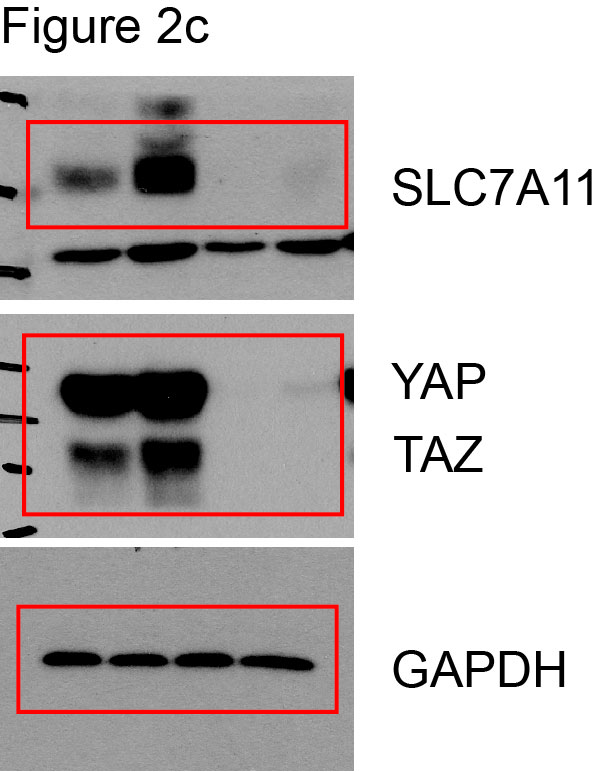

Supplement: Supplementary file 9 — Source Data for Figure 2 [file EMMM-13-e14351-s011.zip › Figure_2_source_data.jpg]

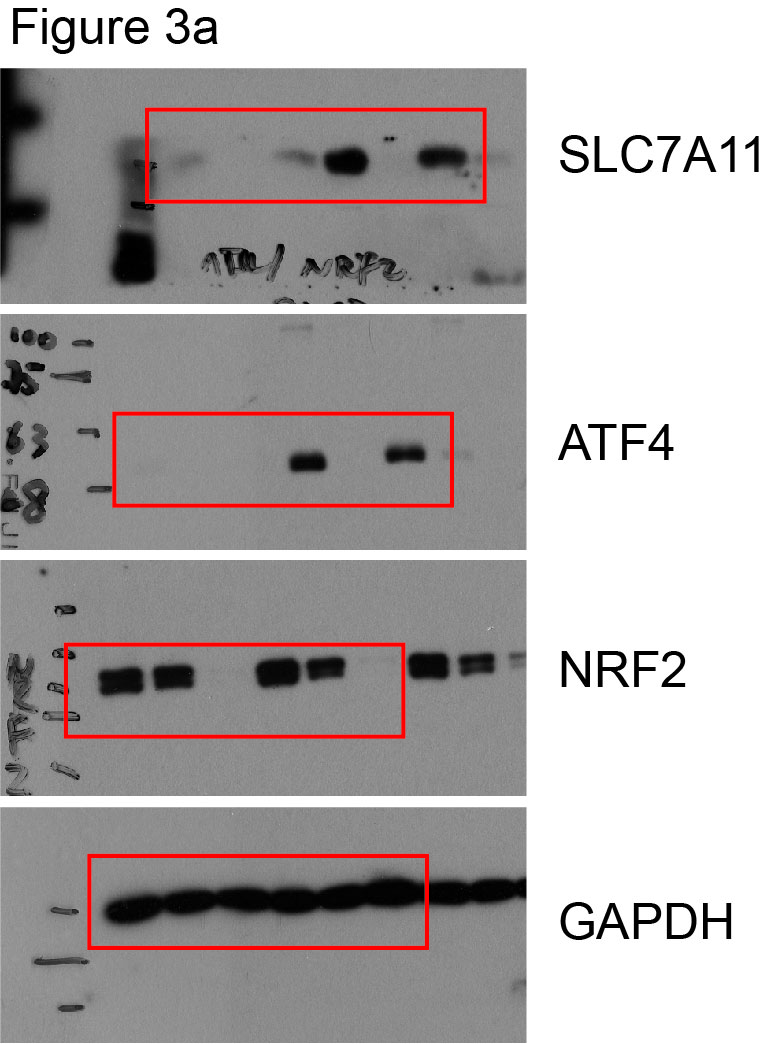

Supplement: Supplementary file 10 — Source Data for Figure 3 [file EMMM-13-e14351-s013.zip › Figure_3_source_data.jpg]

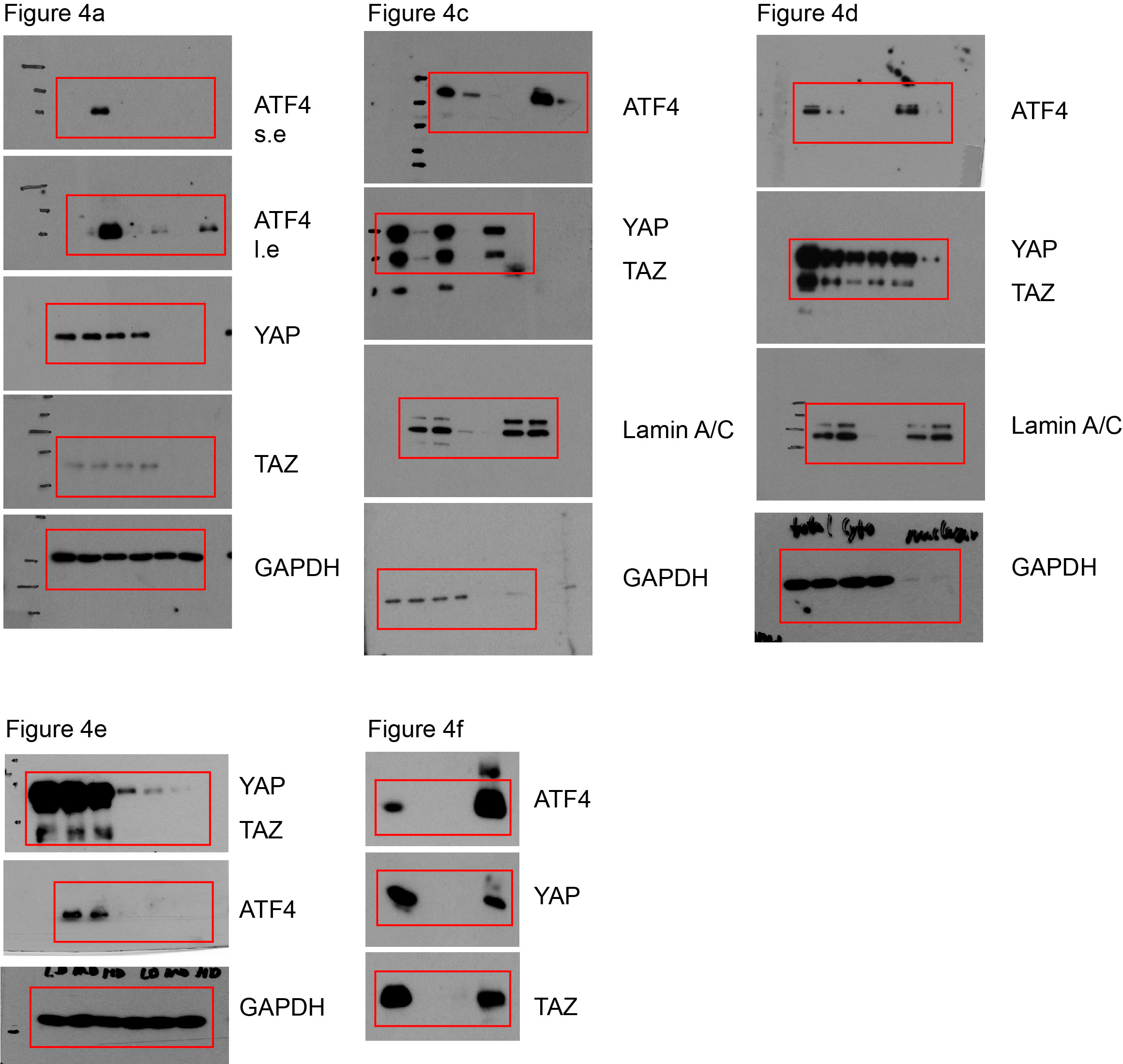

Supplement: Supplementary file 11 — Source Data for Figure 4 [file EMMM-13-e14351-s004.jpg]

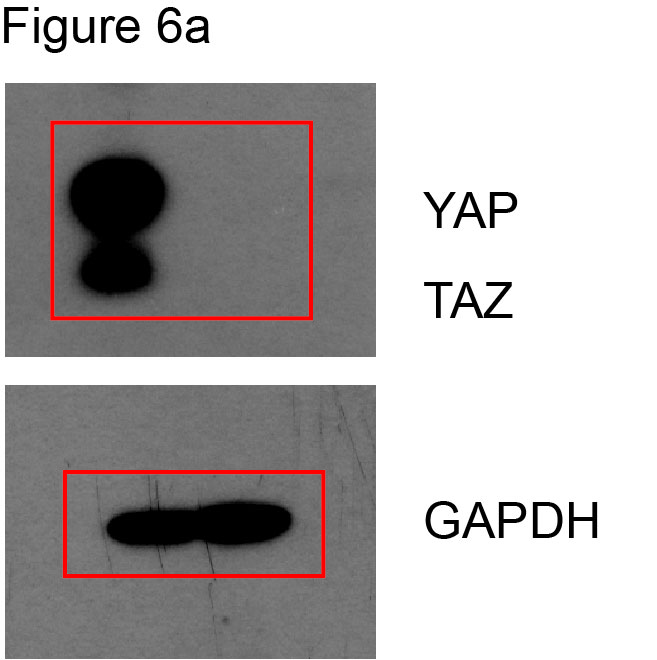

Supplement: Supplementary file 13 — Source Data for Figure 6 [file EMMM-13-e14351-s009.jpg]
