# Supplementary material for: YAP/TAZ and ATF4 drive resistance to Sorafenib in hepatocellular carcinoma by preventing ferroptosis
Source: EMBO Mol Med. 2021 Oct 19;13(12):e14351. doi: 10.15252/emmm.202114351 (PMC8649869; doi:10.15252/emmm.202114351)
Supplement: Supplementary file 14 [file EMMM-13-e14351-s005.pdf]

EMBO Molecular Medicine

YAP/TAZ and ATF4 drive resistance to Sorafenib in hepatocellular carcinoma by preventing ferroptosis

Gerhard Christofori, Ruize Gao, Ravi Kalathur, Mairene Coto-Llerena, Caner Ercan, David Büchel, Shuang Song, Salvatore Piscuoglio, Michael Dill, Fernando Camargo, and Fengyuan Tang
DOI: 10.15252/emmm.202114351

Corresponding authors: Gerhard Christofori (Gerhard.Christofori@unibas.ch) , Fengyuan Tang (tangfengyuan@gmail.com)

Review Timeline:

Submission Date:	30th Mar 21
Editorial Decision:	14th Apr 21
Revision Received:	21st Aug 21
Editorial Decision:	15th Sep 21
Revision Received:	29th Sep 21
Accepted:	4th Oct 21

Editor: Lise Roth

Transaction Report:

(Note: With the exception of the correction of typographical or spelling errors that could be a source of ambiguity, letters and reports are not edited. Depending on transfer agreements, referee reports obtained elsewhere may or may not be included in this compilation. Referee reports are anonymous unless the Referee chooses to sign their reports.)

14th Apr 2021

Dear Prof. Christofori,

Thank you for the submission of your manuscript to EMBO Molecular Medicine. We have now heard back from the three referees who agreed to evaluate your manuscript. As you will see below, the referees all mention the interest of the study, but they also raise substantial concerns on your work, which should be convincingly addressed in a major revision of the present manuscript.

Addressing the reviewers' concerns in full will be necessary for further considering the manuscript in our journal. However, we understand that adding new in vivo experiments would be time-consuming and might fall beyond the scope of the study. Therefore, while a nice addition, such experiments will not be required for acceptance of the manuscript, but limitations should then be discussed.
As revising the manuscript according to the referees' recommendations appears to require a lot of additional work and experimentation, and given the potential interest of your findings, we are ready to extend the deadline to 6-9 months with the understanding that acceptance of the manuscript would entail a second round of review. Should you find that the requested revisions are not feasible within the constraints outlined here and prefer, therefore, to submit your paper elsewhere, we would welcome a message to this effect.

EMBO Molecular Medicine encourages a single round of revision only and therefore, acceptance or rejection of the manuscript will depend on the completeness of your responses included in the next, final version of the manuscript. For this reason, and to save you from any frustrations in the end, I would strongly advise against returning an incomplete revision.

***
When submitting your revised manuscript, please carefully review the instructions that follow below. Failure to include requested items will delay the evaluation of your revision:

1) A .docx formatted version of the manuscript text (including legends for main figures, EV figures and tables). Please make sure that the changes are highlighted to be clearly visible.

2) Individual production quality figure files as .eps, .tif, .jpg (one file per figure).

3) A .docx formatted letter INCLUDING the reviewers' reports and your detailed point-by-point responses to their comments. As part of the EMBO Press transparent editorial process, the point-by-point response is part of the Review Process File (RPF), which will be published alongside your paper.

4) A complete author checklist, which you can download from our author guidelines (https://www.embopress.org/page/journal/17574684/authorguide#submissionofrevisions). Please insert information in the checklist that is also reflected in the manuscript. The completed author checklist will also be part of the RPF.

5) Before submitting your revision, primary datasets produced in this study need to be deposited in an appropriate public database (see https://www.embopress.org/page/journal/17574684/authorguide#dataavailability).
Please remember to provide a reviewer password if the datasets are not yet public.
The accession numbers and database should be listed in a formal "Data Availability " section (placed after Materials & Method).
Please note that the Data Availability Section is restricted to new primary data that are part of this study.

6) We would also encourage you to include the source data for figure panels that show essential data. Numerical data should be provided as individual .xls or .csv files (including a tab describing the data). For blots or microscopy, uncropped images should be submitted (using a zip archive if multiple images need to be supplied for one panel). Additional information on source data and instruction on how to label the files are available at
.

7) Our journal encourages inclusion of *data citations in the reference list* to directly cite datasets that were re-used and obtained from public databases. Data citations in the article text are distinct from normal bibliographical citations and should directly link to the database records from which the data can be accessed. In the main text, data citations are formatted as follows: "Data ref: Smith et al, 2001" or "Data ref: NCBI Sequence Read Archive PRJNA342805, 2017". In the Reference list, data citations must be labeled with "[DATASET]". A data reference must provide the database name, accession number/identifiers and a resolvable link to the landing page from which the data can be accessed at the end of the reference. Further instructions are available at .

8) We replaced Supplementary Information with Expanded View (EV) Figures and Tables that are collapsible/expandable online. A maximum of 5 EV Figures can be typeset. EV Figures should be cited as 'Figure EV1, Figure EV2" etc... in the text and their respective legends should be included in the main text after the legends of regular figures.

- For the figures that you do NOT wish to display as Expanded View figures, they should be bundled together with their legends in a single PDF file called *Appendix*, which should start with a short Table of Content. Appendix figures should be referred to in the main text as: "Appendix Figure S1, Appendix Figure S2" etc.

- Additional Tables/Datasets should be labeled and referred to as Table EV1, Dataset EV1, etc. Legends have to be provided in a separate tab in case of .xls files. Alternatively, the legend can be supplied as a separate text file (README) and zipped together with the Table/Dataset file.
See detailed instructions here:
.

9) For more information: There is space at the end of each article to list relevant web links for further consultation by our readers. Could you identify some relevant ones and provide such information as well? Some examples are patient associations, relevant databases, OMIM/proteins/genes links, author's websites, etc...

10) Every published paper now includes a 'Synopsis' to further enhance discoverability. Synopses are displayed on the journal webpage and are freely accessible to all readers. They include a short stand first (maximum of 300 characters, including space) as well as 2-5 one-sentences bullet points that summarizes the paper. Please write the bullet points to summarize the key NEW findings. They should be designed to be complementary to the abstract - i.e. not repeat the same text. We encourage inclusion of key acronyms and quantitative information (maximum of 30 words / bullet point). Please use the passive voice. Please attach these in a separate file or send them by email, we will incorporate them accordingly.

Please also suggest a striking image or visual abstract to illustrate your article. If you do please provide a png file 550 px-wide x 400-px high.

11) As part of the EMBO Publications transparent editorial process initiative (see our Editorial at http://embomolmed.embopress.org/content/2/9/329), EMBO Molecular Medicine will publish online a Review Process File (RPF) to accompany accepted manuscripts.
In the event of acceptance, this file will be published in conjunction with your paper and will include the anonymous referee reports, your point-by-point response and all pertinent correspondence relating to the manuscript. Let us know whether you agree with the publication of the RPF and as here, if you want to remove or not any figures from it prior to publication.
Please note that the Authors checklist will be published at the end of the RPF.

EMBO Molecular Medicine has a "scooping protection" policy, whereby similar findings that are published by others during review or revision are not a criterion for rejection. Should you decide to submit a revised version, I do ask that you get in touch after six months if you have not completed it, to update us on the status.

I look forward to receiving your revised manuscript.

Yours sincerely,

Lise Roth

Lise Roth, PhD
Editor
EMBO Molecular Medicine

***** Reviewer's comments *****

Referee #1 (Remarks for Author):

Review of manuscript EMM-2021-14351 - YAP/TAZ and ATF4 collaboratively drive resistance to Sorafenib therapy in hepatocellular carcinoma by preventing ferroptosis
In the present manuscript the authors set to identify molecular mechanism conferring resistance to Sorafenib in hepatocellular carcinoma (HCC) cell lines. The authors have identified an unexpected role of YAP/TAZ in preventing ferroptosis, via a mechanism involving ATF4 upregulation of system Xc- (SLC7A11/SLC3A2). The study is interesting and adds to the complex role of YAP/TAZ suggesting a context dependent role in ferroptosis regulation.
I provide to the authors a series of remarks they might consider addressing in order clarify and/or improve a few sections in their manuscript and potentially substantiate their findings

1. In figure 1 the authors show a marked upregulation of YAP/TAZ in the resistant cells (Figure 1C) and subsequently show that the knockdown of YAP/TAZ in these cells decreases colony forming capacity. Is this also happening in naïve HCC cells? Or the YAP/TAZ dependency is exclusive to the resistant pairs - would be helpful to include the naïve cell in the panel presented in figure 1d.
2. The viability assay are performed only using a clonogenic assay and in most cases is not obvious if the effect of Y/T shRNA on sensitizing to ferroptosis are only due to the lower cell density (Figure 1D abd Figure 1H). This is particularly important given the already reported effect of cell density on ferroptosis. https://www.biorxiv.org/content/10.1101/417949v2.full and PMID 31341276 among others. This seems important given that lower cell density also is reported to generate an increase dependency on system Xc in order to provide sufficient cysteine for GSH synthesis.
3. Another issue is that the authors have carried their work almost exclusively using a single shRNA. It would be important that the authors provide at least for some of the key experiments a "rescue" using siRNA resistant constructs. In case they have been validated elsewhere please describe.
4. Regarding the HCC pairs used (naïve and resistant and the corresponding shRNA expressing cells) it would be important for the authors to provide a more detailed characterization in the context of ferroptosis regulators (at least the more established ones such as GPX4, AIFM2, ZEB1 and ACSL4) - interestingly in their RNAseq data its already observable that ACSL4 is markedly downregulated in the resistant cells. Also the authors should provide more insights into the resistance by comparing the response of the cell lines to different ferroptosis stimuli not only system Xc- inhibitors.
5. The downregulation of ACSL4 could be a particular enlightening observation in the context presented here. Loss of ACSL4 is expected to markedly shift the cell lipidome from a more unsaturated state to a more saturated one. Increase membrane saturation has been reported to increase basal expression of ATF4, please see for example (22246806 and 22628618). Currently its not obvious how YAP/TAZ would impact on this but this could be easily addressed by further characterizing the cells (point 4). Could it be that the overall protective effect against ferroptosis rests on the YAP/TAZ suppression of ACSL4 expression? Would the ACSL4 forced expression be sufficient to resensitize Sorafenib resistant HCC to ferroptosis.
6. The authors suggest that in vivo ferroptosis is contributing to Sorafenib suppression of tumor growth. This notion is complex and the authors might wish to tune this down - given that the majority of cysteine taken upon in vivo is in its reduced form, the role played by system Xc- could be minor here. Therefore without the analysis of the growth of system Xc- deficient tumors and the respective response to Sorafenib no strong conclusions can be made.
Minor comments
1. Replace Oxidated for Oxidized BODIPY in the figures and text.

Ref

Intercellular interaction dictates cancer cell ferroptosis via NF2-YAP signalling. Wu J, Minikes AM, Gao M, Bian H, Li Y, Stockwell BR, Chen ZN, Jiang X. Nature. 2019 Aug;572(7769):402-406.
Saturated fatty acid induction of endoplasmic reticulum stress and apoptosis in human liver cells via the PERK/ATF4/CHOP signaling pathway. Cao J, Dai DL, Yao L, Yu HH, Ning B, Zhang Q, Chen J, Cheng WH, Shen W, Yang ZX. Mol Cell Biochem. 2012 May;364(1-2):115-29.
Activating transcription factor 4 regulates stearate-induced vascular calcification. Masuda M, Ting TC, Levi M, Saunders SJ, Miyazaki-Anzai S, Miyazaki M. J Lipid Res. 2012 Aug;53(8):1543-52.

Referee #2 (Remarks for Author):

In the presented manuscript, Gao et al. present data on their investigations of sorafenib resistance in liver cancer. They report that these tumors escape from ferroptosis, an inflammatory form of cell death by regulated necrosis. Ferroptosis depends on iron catalyzed lipid peroxidation. Mechanistically, the authors provide evidence for the regulation of ferroptosis by YAP/TAZ and ATF4. A genome-wide pooled lentiviral shRNA-based lethal screen (barcode amplification) was used to identify these factors. While it is timely and interesting to investigate factors that regulate ferroptosis, especially in a model of liver cancer and sorafenib resistance, several technical and conceptual concerns are listed below. Without a clear readout system for ferroptosis, this title is hardly supported by sufficient data and I cannot recommend this paper for publication in Embo Molecular Medicine. I hope that some of the concerns are helpful to the authors to improve the interesting manuscript.

Major concerns
• The major drawback of this study is the limitation of YAP/TAZ and ATF4 for liver cancer. If these factors do not regulate ferroptosis in general, but exclusively do so in the liver, a more specialized journal appears more appropriate. However, if standard models of ferroptosis, such as e.g. HT1080 cells were affected by YAP/TAZ and ATF4 deficiency or overexpression, the finding would be more relevant to an interdisciplinary readership. Authors should investigate typical ferroptosis-sensitive cells in the presence of appropriate siRNAs or shRNAs or crKO of YAP/TAZ and ATF4, and investigate if classical ferroptosis inducers (erastin, RSL3, FIN56, FINO2) exhibit different kinetics of ferroptosis. This could be performed by FACS using annexin V/7AAD, LDH release, ATP content etc. Addition of ferrostatins should complete these assays.
• Along similar lines, and as much as colony formation assays are appreciated, clear readout systems should be added to detect necrotic cell death. In fact, the manuscript deals with ferroptosis, but this conclusion is entirely based on the use of the inhibitor Fer-1 and inadequate readout systems (relative mRNA expression, signal relative to input, normalized relative intracellular GSH and others). None of these readouts allows to conclude on ferroptosis!
• In figure S1D, more repetitions of the entire experimental setup are required to conclude on GSH regulation by these factors. Standard deviations look like they were based on reading out the same samples, not representing independent experiments. The same concerns applies to Figs. S2D and S4B.
• The western blots (loading controls) in Fig. 4d and 4e should be repeated.

Minor Remarks
• There is an antibody listed for cleaved caspase-3 but I cannot find an experiment where this is used. Please check for others!

Referee #3 (Comments on Novelty/Model System for Author):

For most experiments, the number of independent cell lines tested was limited. Suggestions have been included in my comments to the Authors.

Referee #3 (Remarks for Author):

Tang, Christofori and collaborators investigate mechanisms leading to sorafenib (Srf) resistance in Hepatocellular Carcinoma (HCC). By conducting an shRNA screen they identify YAP and TAZ as factors that, by inhibiting ferroptosis, confer resistance to Srf.
They show that YAP/TAZ associate with ATF4 to regulate the expression of the cystine-glutamate antiporter SLC7A11. This proposed role for YAP-TAZ as inhibitors of ferroptosis is at odds with reports that demonstrate that YAP is an activators of ferroptosis (Wu et al, 2019, Yang et al. 2019, Yang et al. 2020).

The manuscript has potential novelty and impact provided that the Authors will be able to prove convincingly that, at least in HCC, YAP/TAZ are inhibitors of ferroptosis and that this leads to resistance to Srf.

Unfortunately, at this stage, the manuscript lacks coherence in the design of the experiments and consistency in the data shown: experiments need to be performed in multiple cell lines, in some instances the interpretation should be revised and additional experiments should be performed to reinforce their claims (see points below).

Also it needs to be clarified whether YAP/TAZ and ATF4 are inhibitors of ferroptosis selectively in Srf-resistant cells or whether elevation (or activation) of YAP/TAZ is sufficient to confer resistance in Srf-sensitive cells (or any cell).

Major points.

1. Suppl figure 2E. As judged from the colony assay shown in Suppl. Figure 2E, HUH7 resistant cells appear to be sensitive to

sorafenib (and not resistant as they should be). Similarly in figure 2h HLE cells seem to be extremely sensitive to Srf 6µM. Can the Authors provide data in support of the claim that these cells are resistant to Sorafenib? This is a key point, since all the manuscript is based on cell lines that should be resistant to Srf.

2. Is the overexpression of YAP or TAZ sufficient to induce resistance to Sorafenib? Aside from the loss of function analysis in Srf-resistant cells, Authors should test whether over-expression of activated YAP (or TAZ) are sufficient to convey resistance to Srf and ferroptosis. Comparative evaluation in other cell lines (non-HCCs) will tell us whether this function of YAP-TAZ is only present in HCC lines.

3. What is the role of ATF4 and of the YAP/TAZ-TEAD-ATF4 complex in transcription and in the inhibition of ferroptosis? In particular the Authors should test whether overexpression of ATF4 alone will be sufficient to induced ferroptotic genes (SLC7A11, ATF3, CHAC1). This will clarify whether YAP/TAZ are dispensable for gene transactivation, but essential for ATF4 accumulation.

4. The effect of ATF4 silencing should be tested using more than a single siRNA, in order to rule out off target effects.

5. Data in Figure 3 (and related text) show that ATF4 regulates SLC7A11 in response to Sorafenib treatment. This is a key evidence, the same experiment needs to be repeated for all the other Srf resistant lines.

6. Line 232. It is not clear how the Authors interpret the loss of viability following ATF4 silencing and how this relates to resistance to Srf. Is this sensitivity a trait acquired when cells became resistant to Srf? Are Srf-resistant cells more sensitive to ATF4 depletion than Srf-sensitive cells? If so, why? Is this because of "tonic" ER-stress signalling? Along with increased level of YAP/TAZ, have Srf-resistant cells also more ATF4?

7. Figure 7H show that blocking ferroptosis by Ferrostatin-1 restores Sorafenib resistance in YAP/TAZ silenced HLE cells. The same rescue needs to be performed in HUH7 and Hep3B cells.

8. Loss of YAP/TAZ in HLE cells leads to increased lipid oxidation, suggesting that YAP/TAZ are implicated in the regulation of intracellular GSH. Is this only happening in Sorafenib resistant cells? Or alternatively YAP/TAZ regulates the intracellular red-ox also in Sorafenib sensitive cells?

9. If activation of YAP/TAZ confers resistance to Srf by inhibiting ferroptosis, than Erastin should make resistant cells (i.e over-expressing YAP/TAZ) sensitive to Srf. This should be tested.

10. In vivo experiments were performed only on SNU398 cells. Oddly this line was not used in all the in-vitro experiments. For coherence and to strengthen the manuscript, Authors need to (i) show that SNU398 cells are Srf-resistant and that resistance is mediated by YAP/TAZ and ATF4, (ii) reinforce the in-vivo evidences by using additional Srf-resistant cell lines.

11. Line 147: ChIP does not have the resolution to demonstrate binding to a transcription factor binding site. The PCR signal only shows that the immunoprecipitated chromatin contains the TEAD-binding motif, but the TF used for the IP could be binding a nearby sequence contained in the immunoprecipitated DNA. The same holds for the sentence starting at line 308, concerning regulation by the AARE-motif, and the paragraph starting at line 312. Thus, the interpretation of these experimental evidences should be revised and in should not be used as an evidence for sequence specific binding

Minor points

12. Figure 1C. TAZ seems to be modified upon Srf treatment as suggested by the altered migration in WB, have the Authors investigated this? This may reflect selective activation of TAZ (and possible YAP) upon Srf treatment.

13. Are ATF4 level changing with cell density? (see fig. 4D)

14. Line 204. This sentence reads: "Moreover, the forced expression of SLC7A11 in YAP/TAZ knock-down cells was able to prevent loss of YAP/TAZ-induced cell death in response to Sorafenib...." This sentence is difficult to read, if possible I kindly ask the Authors to amend it.

15. Line 219. To my knowledge the reference cited (Dixon, 2014) does not show that Sorafenib does not induce YAP/TAZ activity, as the Authors state here. Please clarify and if needed amend.

Point-by-point reply to the reviewers' comments

Journal: EMBO Molecular Medicine
Manuscript: EMM-2021-14351

YAP/TAZ and ATF4 drive resistance to Sorafenib therapy in hepatocellular carcinoma by preventing ferroptosis

Ruize Gao, Ravi K.R. Kalathur, Mairene Coto-Llerena, Caner Ercan, David Buechel, Song Shuang, Salvatore Piscuoglio, Michael T. Dill, Fernando D. Camargo, Gerhard Christofori, and Fengyuan Tang

Introductory Remarks

We highly appreciate the constructive comments and suggestions by the editor and by the reviewers on our manuscript.

We have now spent the past 2-3 months to adequately address all the criticisms raised by the reviewers. In brief, we have 1. added another readout to mark ferroptosis by determining the cellular ATP content via a cell viability assay as suggested by the reviewers, 2. updated gain of function studies, 3. examined the expression of classical ferroptosis-related genes by quantitative RT-PCR, 4. performed more biological replicates on GSH measurement to validate the statistical significance of the results, 5. employed additional individual siRNAs to avoid off-target effects, 6. used additional inducers and inhibitors of different types of cell death to conclude on ferroptosis, and 7. included additional cancer cell lines and cancer types in the various analyses.

As a consequence of these revisions, the Figures and Suppl. Figures (Appendix Figures) have been revised and updated in their panels and the number of Appendix Figures has increased from 5 to 9. In addition, the presentation of the results has been revised in the text to accommodate the new data and to appropriately adapt the conclusions.

The details of the revisions can be seen in the point-by-point reply to the reviewers' comments. We copied the reviewers' comments in *italic* and presented our reply in regular font.

Point-by-point reply

Referee #1. *Review of manuscript EMM-2021-14351 - YAP/TAZ and ATF4 collaboratively drive resistance to Sorafenib therapy in hepatocellular carcinoma by preventing ferroptosis. In the present manuscript the authors set to identify molecular mechanism conferring resistance to Sorafenib in hepatocellular carcinoma (HCC) cell lines. The authors have identified an unexpected role of YAP/TAZ in preventing ferroptosis, via a*

mechanism involving ATF4 upregulation of system Xc- (SLC7A11/SLC3A2). The study is interesting and adds to the complex role of YAP/TAZ suggesting a context dependent role in ferroptosis regulation. I provide to the authors a series of remarks they might consider addressing in order clarify and/or improve a few sections in their manuscript and potentially substantiate their findings.

We thank the reviewer for her/his interest in the study and for the constructive comments to improve the quality of the manuscript.

1. In figure 1 the authors show a marked upregulation of YAP/TAZ in the resistant cells (Figure 1C) and subsequently show that the knockdown of YAP/TAZ in these cells decreases colony forming capacity. Is this also happening in naïve HCC cells? Or the YAP/TAZ dependency is exclusive to the resistant pairs - would be helpful to include the naïve cell in the panel presented in figure 1d.

We thank the reviewer for this comment. As the reviewer suggested, we now performed these experiments also in Sorafenib-sensitive (parental) Huh7 and Hep3B cells upon YAP/TAZ deficiency. Indeed, an increase in sensitivity to acute Sorafenib treatment was observed in parental Huh7 and Hep3B cells upon loss of YAP/TAZ. However, we observed that Ferrostatin-1 could not rescue the Sorafenib-induced cell death in control siRNA-transfected parental Huh7 and Hep3B cells as well as upon siRNA-mediated depletion of YAP/TAZ in these cells. These results suggest that the cell death induced by acute Sorafenib treatment of Sorafenib-sensitive HCC cells does not involve ferroptosis, as is the case in the Sorafenib-resistant cells. Instead, as previously reported by our laboratory, the acute treatment of Sorafenib-sensitive HCC cells with Sorafenib induces autophagy and apoptosis which is also prevented by YAP/TAZ activities (Tang *et al*, 2019). In contrast, Sorafenib-resistant cells require YAP/TAZ activity to maintain Sorafenib resistance by overcoming ferroptotic cell death, as demonstrated in this report.

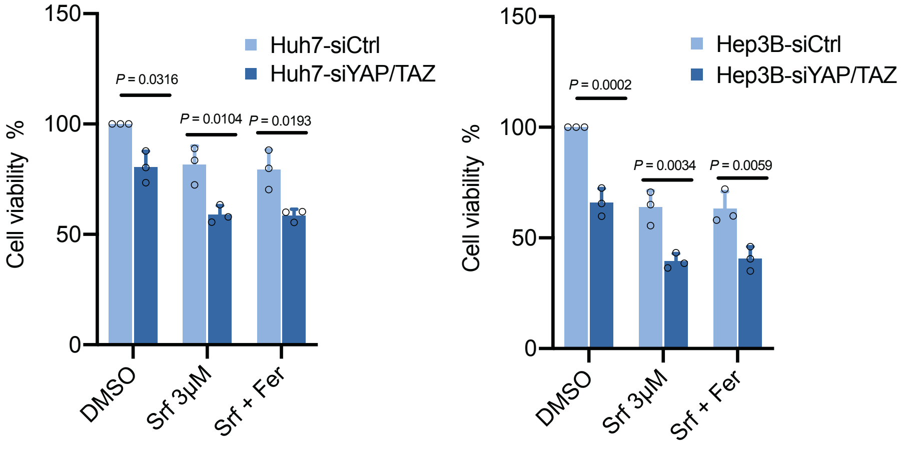

Cell viability assay showing that the loss of YAP/TAZ increased death of Sorafenib-sensitive (parental) Huh7 and Hep3B cells in response to Sorafenib treatment which could be reversed by treatment with Ferrostatin-1. Huh7 and Hep3B cells were seeded into 60mm dishes and transfected with siYAP/TAZ, 24 hours later after the transfection, cells were seeded into 96-well plates with 5000 cells/well, and treated with 3μM Sorafenib (Srf) with or without 5μM Ferrostatin-1 (Fer) treatment for 12 hours before harvest. Cell viability was measured with Promega CellTiter-Glo 2.0 kit and normalized to siCtrl-DMSO. Statistical significance was calculated using two-way ANOVA. Results represent three independent experiments.

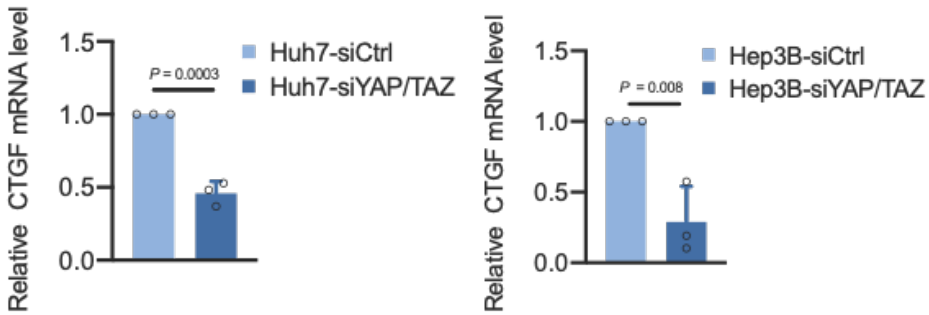

Quantitative RT-PCR analysis confirmed the knockdown efficiency of YAP/TAZ as determined from the experiment shown above. RNA was extracted and analyzed by quantitative RT-PCR. Expression of *CTGF* as a direct transcritional target gene of YAP/TAZ was used to assess the knockdown efficiency of siYAP/TAZ. Statistical significance was calculated using unpaired t-test. Results represent three independent experiments.

Given that YAP/TAZ expression was significantly lower in Sorafenib-sensitive Huh7 and Hep3B cells as compared to their resistant counterparts (Figure 1c), we now also assessed whether the forced expression of YAP/TAZ could overcome cell death induced by Sorafenib treatment of Sorafenib-sensitive cells. Indeed, the forced expression of a constitutively active version of YAP (YAP-5SA) prevented Sorafenib-induced cell death, further indicating that YAP/TAZ can promote Sorafenib resistance in HCC cells.

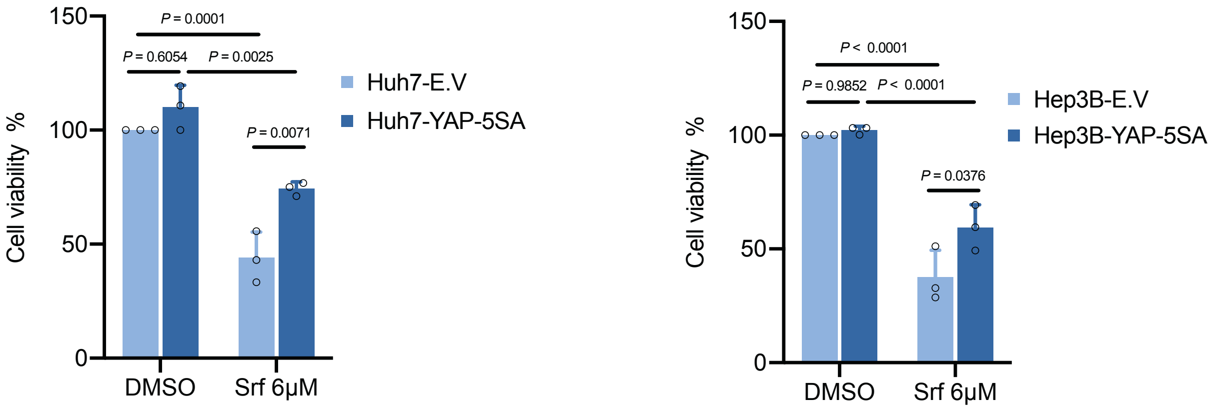

Activated YAP overexpression confers Sorafenib resistance in Huh7 and Hep3B cells. Huh7 and Hep3B cells transfected with empty vector (E.V) control or with a cDNA construct coding for YAP-5SA were seeded into 96-well plate with 5000 cells/well and cultured with either DMSO or 6μM Sorafenib (Srf) for 12 hours before harvest. Cell viability was measured with Promega CellTiter-Glo 2.0 kit and normalized to Huh7 and Hep3B transfected with empty vector (E.V) and treated with DMSO solvent. Statistical significance was calculated using two-way ANOVA. Results represent three independent experiments.

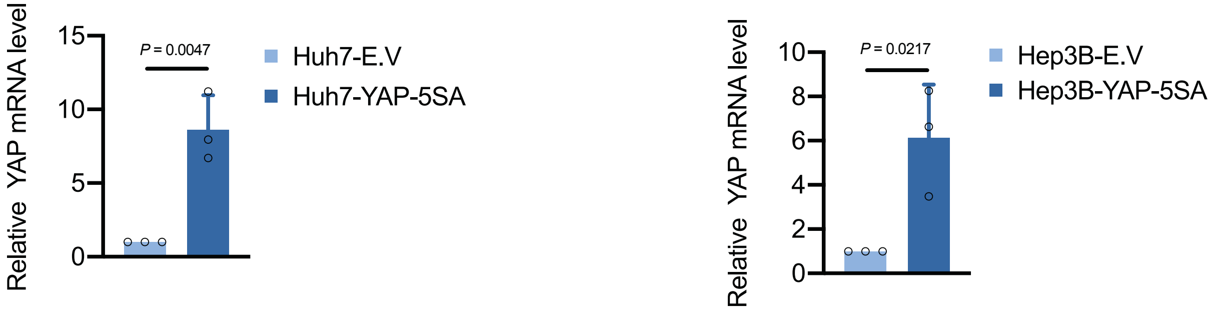

Quantitative RT-PCR analysis confirmed the overexpression of activated YAP. RNA was extracted from the cells described above and analyzed by quantitative RT-PCR. Statistical significance was calculated using Unpaired t-test. Results represent three independent experiments.

These results are now presented in the updated Appendix Figure S3a-d and presented in the text in lines 167-174.

2. The viability assay are performed only using a clonogenic assay and in most cases is not obvious if the effect of Y/T shRNA on sensitizing to ferroptosis are only due to the lower cell density (Figure 1D abd Figure 1H). This is particularly important given the already reported effect of cell density on ferroptosis. https://www.biorxiv.org/content/10.1101/417949v2.full and PMID 31341276 among others. This seems important given that lower cell density also is reported to generate an

increase dependency on system Xc in order to provide sufficient cysteine for GSH synthesis.

We thank the reviewer for this comment. As the reviewer suggested, low cell density addict cancer cells to System Xc. Indeed, our colony formation was performed at a very low cell density (starting with 2000 cells/well in a 6-well plate).
To further support our conclusion, we added as an analytical readout the assessment of cell viability using the Promega Celltiter Glo^{TM} assay. In line with the results of the colony formation assays, the cell viability results confirmed the conclusion that YAP/TAZ promotes Sorafenib resistance via blocking ferroptosis in YAP/TAZ^{high} cells (updated Appendix Figure S1i).

Quantitative RT-PCR analysis on the expression of *CTGF*, a direct transcritional target gene of YAP and TAZ, was used to determine the knock-down efficiency of YAP/TAZ activities (updated Appendix Figure S1j)

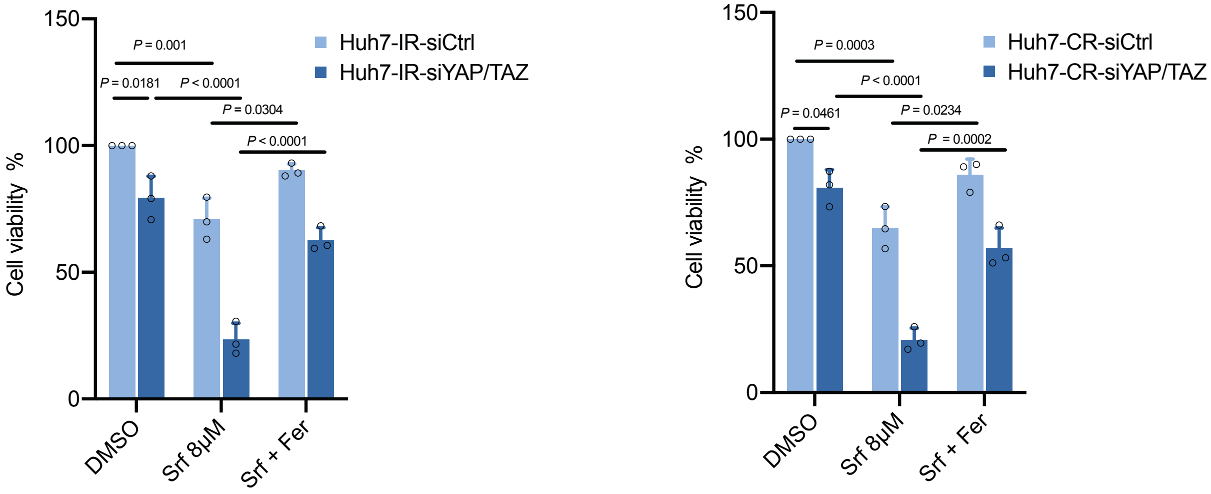

Cell viability assay showing that the knockdown of YAP/TAZ in Sorafenib-resistant Huh7-IR cells (left panel) and Huh7-CR cells (right panel) induced higher rates of cell death in response to Sorafenib (Srf; 8µM) treatment which could be overcome by treatment with Ferrostatin-1 (Fer; 5µM) 12 hours before harvest. Cell viability was measured with Promega CellTiter-Glo 2.0 kit and normalized to siCtrl-DMSO. Statistical significance was calculated using two-way ANOVA. Results represent three independent experiments.

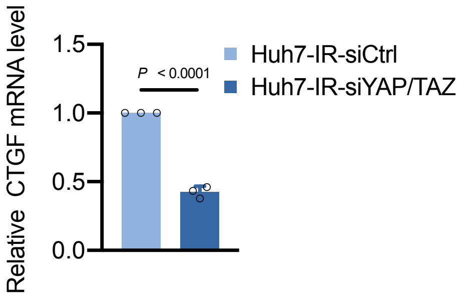

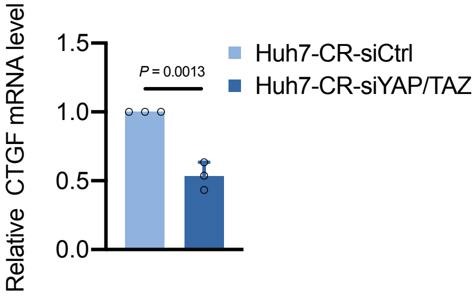

Knockdown efficiency of siYAP/TAZ was assessed by quantitative RT-PCR analysis. RNA was extracted from the cells described in (i) and analyzed by quantitative RT-PCR. *CTGF* as a direct transcriptional target of YAP/TAZ served as positive control to confirm the knockdown efficiency of siYAP/TAZ. Statistical significance was calculated using unpaired t-test. Results represent three independent experiments.

These results are now presented in the updated Appendix Figure S1i and j and presented in the text in lines 150-154.

3. Another issue is that the authors have carried their work almost exclusively using a single shRNA. It would be important that the authors provide at least for some of the key experiments a "rescue" using siRNA resistant constructs. In case they have been validated elsewhere please describe.

We thank the reviewer for the suggestions. The shRNA targeting both YAP/TAZ with a sequence of “TGTGGATGAGATGGATACA” used in this study was adapted from a previous report (Hiemer *et al*, 2015; now cited in the Appendix Methods section). In addition to the use of shRNA, On-target Plus siRNA pools from Horizon Discovery were used across the experiments. The results generated with different reagents from separate vendors all supported the same conclusion: YAP/TAZ and ATF4 promote Sorafenib resistance via the upregulation SLC7A11. Knock-down efficiencies are shown in all experiments.

Furthermore, key experimental evidence was also generated by gain of function (GOF) approaches. For instance, the forced expression of a constitutive-active version of YAP strikingly induced the expression of SLC7A11 and overcame Sorafenib-induced cell death. In addition, protein stabilization of ATF4 and its nuclear translocation was significantly instructed by the expression of a constitutive-nuclear version of TAZ.

We appreciate the reviewer's suggestions of confirmation of key results via a rescue experiment. However, during the revision period, we did not succeed in obtaining stable shRNA/siRNA-resistant YAP/TAZ or ATF4-overexpressing cell lines. We hope that the various independent experimental results now generated with different cancer type cell lines and via both LOF and GOF studies convince this reviewer on the aptness of our conclusion.

4. Regarding the HCC pairs used (naïve and resistant and the corresponding shRNA expressing cells) it would be important for the authors to provide a more detailed characterization in the context of ferroptosis regulators (at least the more established ones such as GPX4, AIFM2, ZEB1 and ACSL4) - interestingly in their RNAseq data its already observable that ACSL4 is markedly downregulated in the resistant cells. Also the authors should provide more insights into the resistance by comparing the response of the cell lines to different ferroptosis stimuli not only system Xc-inhibitors.

We thank the reviewer for the important comment. As suggested, we have now determined the mRNA expression of ferroptotic regulators in Sorafenib-resistant cells. Indeed, we observed that the expression of ACSL4 was significantly down-regulated in resistant cells (see figure below, only to be seen by the reviewers). How ACSL4 is downregulated and how ACSL4 mechanistically affects Sorafenib resistance is certainly of interest and will motivate future studies. However, as discussed and shown under Point 5 by this reviewer, in contrast to the expression of SLC7A11, the expression of ACSL4 and the other ferroptosis regulators analyzed was not apparently regulated by YAP and TAZ and, hence, we believe that these regulators may exert YAP/TAZ-independent roles in ferroptosis regulation and hence are out of the scope of this study.

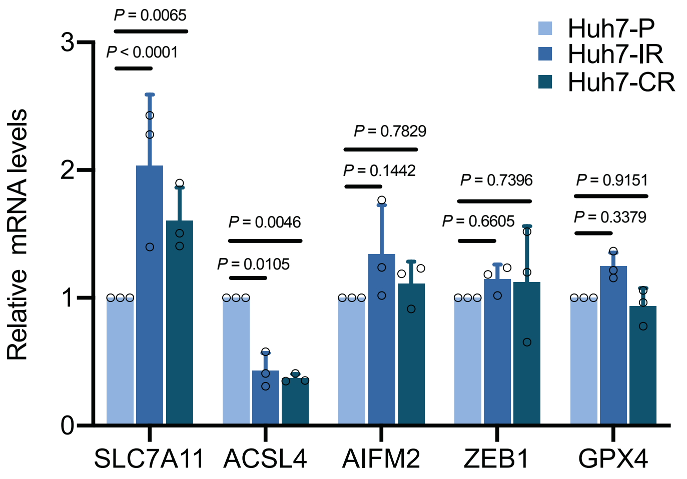

Figure 1 to be seen by reviewers. Quantitative RT-PCR analysis of ferroptotic genes in Sorafenib-sensitive Huh7 and and Sorafenib-resistant Huh7-IR and Huh7-CR cell lines. Huh7-Parental/IR/CR cells were seeded into 60mm dishes and RNA was extracted once cell density reached 80% percent. The expression of various ferroptotic regulators was determined by quantitative RT-PCR. Huh7-IR/CR cells present higher *SLC7A11* and lower *ACSL4* expression than Huh7-parental cells. Statistical significance was calculated using two-way ANOVA. Results represent three independent experiments.

As suggested by the reviewer, we further determined the specific role of ferroptosis as the major cell death pathway which has to be overcome by Sorafenib-resistant HCC cells. We observed that Sorafenib-resistant HCC cells were more resistant to various ferroptosis stimuli than their Sorafenib-sensitive parental cells (updated Appendix Figure S2a).

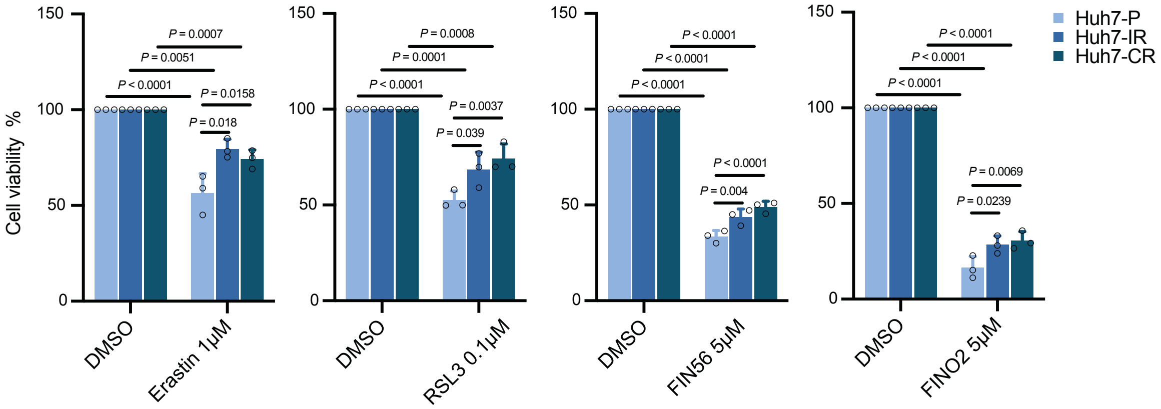

Cell viability assay showing that Sorafenib-resistant Huh7 cells are resistant to various ferroptosis inducers, including Erastin, RSL3, FIN56, and FINO2. Cells were treated with either 1μM Erastin for 24 hours, 0.1μM RSL3 for 12 hours, 5μM FIN56 for 18 hours, or 5μM FINO2 for 18 hours. Cell viability was measured using the Promega CellTiter-Glo 2.0 kit and normalized to the respective DMSO treatments. Statistical significance was calculated using two-way ANOVA. Results represent three independent experiments.

These results are now presented in Appendix Figure S2a and presented in the text in lines 155-159.

5. The downregulation of ACSL4 could be a particular enlightening observation in the context presented here. Loss of ACSL4 is expected to markedly shift the cell lipidome from a more unsaturated state to a more saturated one. Increase membrane saturation has been reported to increase basal expression of ATF4, please see for example (22246806 and 22628618). Currently its not obvious how YAP/TAZ would impact on this but this could be easily addressed by further characterizing the cells (point 4). Could it be that the overall protective effect against ferroptosis rests on the YAP/TAZ suppression

of ACSL4 expression? Would the ACSL4 forced expression be sufficient to resensitize Sorafenib resistant HCC to ferroptosis.

We appreciate the reviewer's comment in the exploring the interplay between YAP/TAZ and ACSL4. Indeed, we observed that ACSL4 was significantly down-regulated in resistant cells as analyzed by RNA sequencing and quantitative RT-PCR (shown above in Figure 1 to be seen by the reviewers). We believe that downregulated ACSL4 could possibly be a marker of Sorafenib resistance in HCC, which also is in line with a previous report (Feng et al. Acta Pharmacol Sin, 2021).
How ACSL4 is downregulated and what the consequent outcome of downregulated ACSL4 in Sorafenib resistance are certainly interesting and may motivate future studies. First gene expression analyses in response to siRNA-mediated depletion of YAP/TAZ in further Sorafenib-resistant HCC cells have revealed that the loss of YAP/TAZ does not result in an increase of ACSL4 expression, as well as other ferroptosis regulators analyzed (see Figure 2 below to be seen by the reviewers). However, we have identified SLC7A11 as the most significant target of concomitant regulation by YAP/TAZ and ATF4, a cooperative regulation which has not been shown and characterized at the mechanistic level before. Hence, we here focus on the role of SLC7A11, GSH and ROS in Sorafenib resistance of HCC cells.

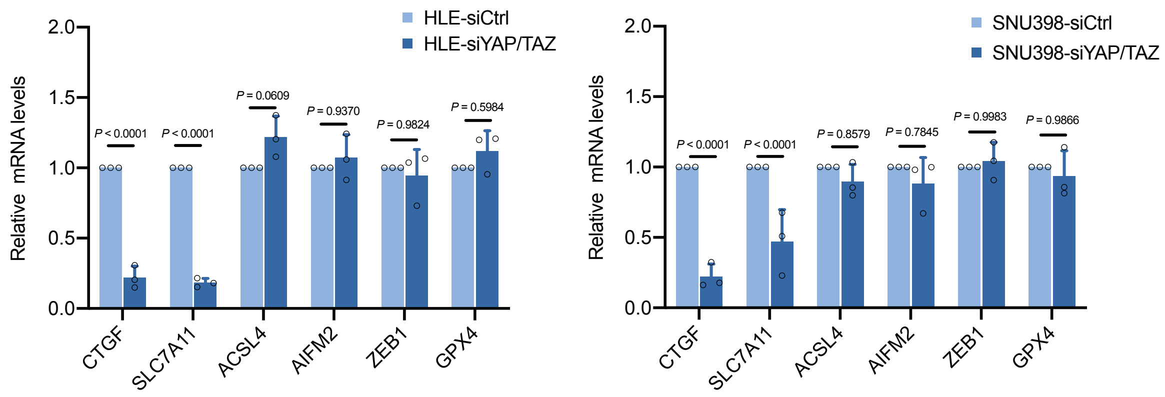

Figure 2 to be seen by the reviewers. Quantitative RT-PCR analysis of the expression of ferroptotic genes upon YAP/TAZ deficiency in HLE and SNU398 HCC cells. Either HLE or SNU398 cells were seeded into 60mm dishes and transfected with siYAP/TAZ. 24 hours later, RNA was extracted and analyzed by quantitative RT-PCR. *CTGF* as a direct transcriptional target of YAP/TAZ served as positive control to confirm the ablation of YAP/TAZ transcriptional activities. *SLC7A11* expression has a significant decrease with knockdown of YAP/TAZ but not the other genes. Statistical significance was calculated using Two-way ANOVA. Results represent three independent experiments.

6. The authors suggest that in vivo ferroptosis is contributing to Sorafenib suppression of tumor growth. This notion is complex and the authors might wish to tune this down - given that the majority of cysteine taken upon in vivo is in its reduced form, the role played by system Xc- could be minor here. Therefore without the analysis of the growth of system Xc- deficient tumors and the respective response to Sorafenib no strong conclusions can be made.

We thank the reviewer for this suggestion. We fully agree with this reviewer that the pharmacological inhibition of system Xc- and GSH function does not as convincingly represent a proof of concept experiment in vivo. However, it should be noted that the pharmacological interference will be the approach to be taken in patients. We have now revised the text to down-tune these statements and to avoid any over-interpretation of the in vivo results (updated in text lines 415-418 and 480-484).

Minor comments

1. Replace Oxidated for Oxidized BODIPY in the figures and text.

We have corrected this in figures and text.

Referee #2 (Remarks for Author):

In the presented manuscript, Gao et al. present data on their investigations of sorafenib resistance in liver cancer. They report that these tumors escape from ferroptosis, an inflammatory form of cell death by regulated necrosis. Ferroptosis depends on iron catalyzed lipid peroxidation. Mechanistically, the authors provide evidence for the regulation of ferroptosis by YAP/TAZ and ATF4. A genome-wide pooled lentiviral shRNA-based lethal screen (barcode amplification) was used to identify these factors. While it is timely and interesting to investigate factors that regulate ferroptosis, especially in a model of liver cancer and sorafenib resistance, several technical and conceptual concerns are listed below. Without a clear readout system for ferroptosis, this title is hardly supported by sufficient data and I cannot recommend this paper for publication in Embo Molecular Medicine. I hope that some of the concerns are helpful to the authors to improve the interesting manuscript.

We appreciate for the reviewer's comments and suggestions. We have now substantially expanded on the experiments addressing the specific role of YAP/TAZ-mediated inhibition of ferroptosis underlying Sorafenib resistance in HCC cells and also in other cancer type cells, including the use of additional inducers of ferroptosis, inhibitors of various types of

cell death, and additional cellular readouts, such as measuring cell viability using the Promega Celltiter Glo™ assay according to the three reviewers' comments.

Major concerns

• The major drawback of this study is the limitation of YAP/TAZ and ATF4 for liver cancer. If these factors do not regulate ferroptosis in general, but exclusively do so in the liver, a more specialized journal appears more appropriate. However, if standard models of ferroptosis, such as e.g. HT1080 cells were affected by YAP/TAZ and ATF4 deficiency or overexpression, the finding would be more relevant to an interdisciplinary readership. Authors should investigate typical ferroptosis-sensitive cells in the presence of appropriate siRNAs or shRNAs or crKO of YAP/TAZ and ATF4, and investigate if classical ferroptosis inducers (erastin, RSL3, FIN56, FINO2) exhibit different kinetics of ferroptosis. This could be performed by FACS using annexin V/7AAD, LDH release, ATP content etc. Addition of ferrostatins should complete these assays.

We thank the reviewer for these constructive comments. However, we respectfully disagree that our findings have to be valid in general for all cancer types to be published in EMBO Mol. Med. Sorafenib is one of the current treatments of choice for advanced HCC, yet the development of resistance to this treatment - as is for most targeted therapies in other cancer types - is a major clinical problem. Hence, overcoming Sorafenib resistance may be specific for HCC and thus important for improving patient care in particular, but also for the scientific understanding of therapy resistance in general. Hence, these insights are important to a general field of molecular medicine and cancer research. One precedence for such specific findings with general implications for the understanding of therapy resistance, is the use of PARP inhibitors to overcome chemotherapy resistance in BRCA-deficient breast cancer. This is highly specific for this type of cancer, yet has been of high general interest as a principle of overcoming therapy resistance, and numerous papers have been and are still being published in journals of general science.

Still, in order to address the reviewer's concerns experimentally, we have first expanded the readout of cell viability throughout the manuscript by using an additional method to monitor ferroptosis via measuring ATP content (by Promega Celltiter Glo™ kit), as suggested by the reviewer.
Moreover, to address the generality of findings for ferroptosis regulation, we have further addressed the roles of YAP/TAZ and ATF4 in HT1080 cells in response to a variety of ferroptotic stimuli. Interestingly, YAP/TAZ and ATF4 loss-of-function resulted in higher rates of ferroptosis upon RSL3, FIN56 and FINO2 treatments. More importantly, treatment with Ferrostatin-1 rescued the ferroptotic stimuli-induced cell death, confirming cell death via ferroptosis (updated Appendix Figure S8b).

Interestingly, we observed that there was no difference in the rate of ferroptosis between control-treated and YAP/TAZ or ATF4-deficient HT1080 cells upon Erastin treatment. In fact, a recent report by the Conrad laboratory demonstrated that HT1080 cells fails to response to the xCT blocker Sorafenib (Zheng *et al*, 2021). While both Sorafenib and Erastin target xCT, how these two compounds exert functions so dramatically divergent in HT1080 cells remains unknown; it may be due to the inhibition of other targets of Sorafenib and/or Erastin. The recent study by the Conrad laboratory demonstrated that Sorafenib fails to trigger ferroptosis in xCT^{high} cells. In fact, the key conclusion from our study is that YAP/TAZ and ATF4 drive SLC7A11 expression, thereby restricting Sorafenib-induced ferroptosis. Thus, our conclusion is fully supported by the recently published study.

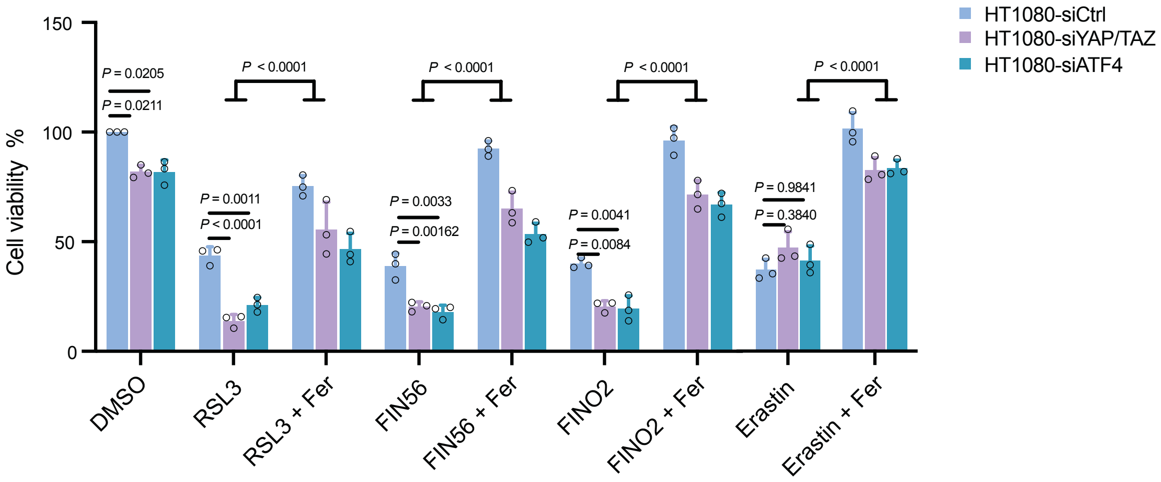

Cell viability assay showing that upon either YAP/TAZ or ATF4 deficiency in HT1080 cells treatments with RSL3, FIN56, or FINO2 resulted in increased cell death which could be overcome by treatment with Ferrostatin-1. In contrast, treatment with Erastin induced comparable rates of ferroptosis in the presence or absence of YAP/TAZ or ATF4. HT1080 cells were transfected with either siYAP/TAZ or siATF4 and 24 hours later treated with 0.3µM RSL3, 1µM FIN56, or 0.5µM FINO2 with or without 5µM Ferrostatin-1 for 20 hours before harvest. Cell viability was measured using Promega CellTiter-Glo 2.0 kit and normalized to siCtrl-DMSO. Statistical significance was calculated using two-way ANOVA. Results represent 3 independent experiments.

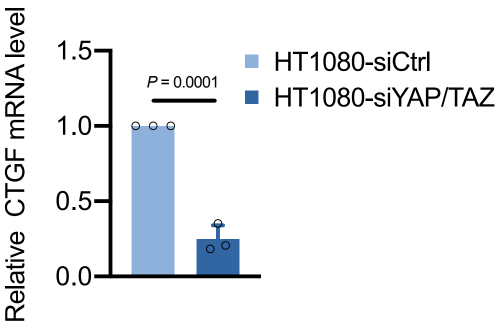

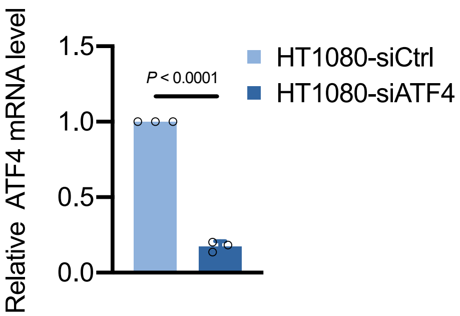

Quantitative RT-PCR analysis confirmed the knockdown efficiency of YAP/TAZ and ATF4 in HT1080 described in (b). *CTGF* as a direct transcriptional target gene of YAP/TAZ served as positive control to confirm the knockdown efficiency of siYAP/TAZ. Statistical significance was calculated using unpaired t-test. Results represent three independent experiments.

We now present these data in Appendix Figure S8b and c and in lines 367-379 in the text and in lines 464- 476 in the Discussion section.

To further assess a general role of YAP/TAZ in restricting ferroptosis, we analyzed Sorafenib and RSL-3-induced ferroptosis in MDA-MB-231 human breast cancer cells, a cell line widely used in the breast cancer community to study breast cancer metastasis. In line with our conclusion, overexpressed YAP can induce ferroptosis resistance in MDA-MB-231 cell as well (updated Appendix Figure S8d, e).

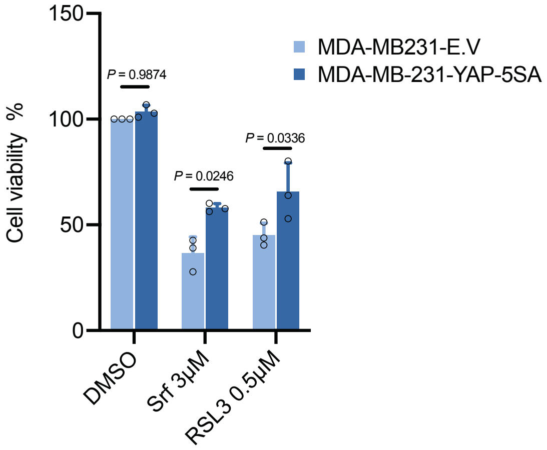

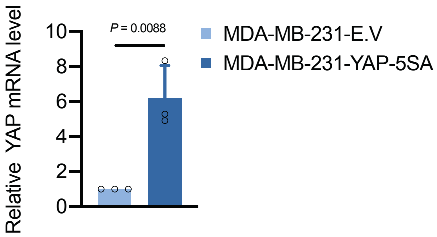

(Left panel) Activated YAP overexpression confers resistance to Sorafenib and RSL3 in MDA-MB-231 cells. MDA-MB-231 (empty vector control (EV) or YAP-5SA-expressing cells were treated with either DMSO or 3μM Sorafenib or 0.5μM RSL3 for 12 hours before harvest. Cell viability was measured with Promega CellTiter-Glo 2.0 kit and normalized to MDA-MB-23-EV + DMSO. Statistical significance was calculated using two-way ANOVA. Results represent three independent experiments.

(Right panel) Quantitative RT-PCR analysis confirmed the overexpression of activated YAP in MDA-MB-231. RNA was extracted from the cells described above and analyzed by quantitative RT-PCR. Statistical significance was calculated using unpaired t-test. Results represent three independent experiments.

These data are now presented in Appendix Figure S8d and e and in the results section in lines 380-384.

• *Along similar lines, and as much as colony formation assays are appreciated, clear readout systems should be added to detect necrotic cell death. In fact, the manuscript deals with ferroptosis, but this conclusion is entirely based on the use of the inhibitor Fer-1 and inadequate readout systems (relative mRNA expression, signal relative to input, normalized relative intracellular GSH and others). None of these readouts allows to conclude on ferroptosis!*

We thank the reviewer for these constructive comments. In light of this suggestion, we have included in all additional experiments another readout by measuring ATP contents using Cell-titer^{TM}-Glo.

As suggested by the reviewers, we analyzed the role of YAP/TAZ and ATF4 in overcoming ferroptosis induced by additional well-characterized inducers, such as Erastin, RSL-3, FIN56 and FINO2, and its inhibition by Ferrostatin-1, highlighting a robustness and reliable ferroptosis system in our study (new Appendix Figure S2a; see also response to point 4 by Reviewer 1).

To further demonstrate the specificity of a role of YAP/TAZ in ferroptotic cell death, yet to exclude apoptosis or necroptosis, we treated siCtrl and siYAP/TAZ-transfected HLE cells with Sorafenib and with the ferroptosis inhibitor Ferrostatin-1, with the pan-caspase inhibitor Z-VAD-FMK to inhibit apoptosis or with the RIPK3 inhibitor GSK-872 to repress necroptosis. Interestingly, only Ferrostatin-1 rescued cell viability, thus confirming a specific role of YAP/TAZ in regulating Sorafenib-induced ferroptosis new Appendix Figure S2b).

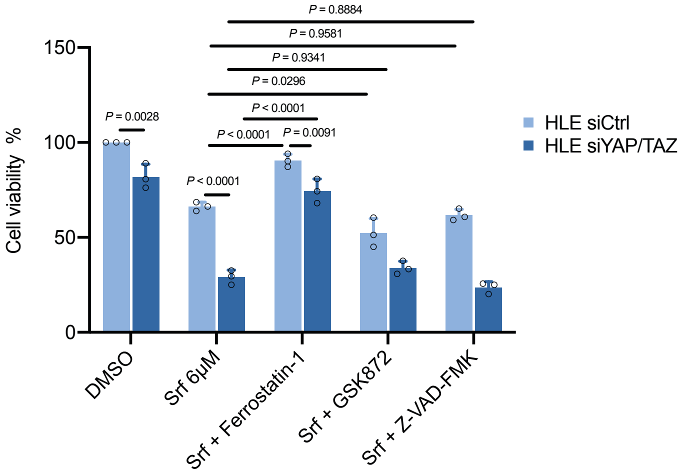

YAP/TAZ-deficiency induced death of HLE cells in response to Sorafenib treatment could be rescued by Ferrostatin-1 but not by GSK872 or Z-VAD-FMK. HLE cells transfected with either siCtrl or siYAP/TAZ were treated with 6μM Sorafenib (Srf) with or without 5μM Ferrostatin-1, 10μM GSK872 or 10μM Z-VAD-FMK for 20 hours before harvest. Cell viability was measured using Promega CellTiter-Glo 2.0 kit and normalized to siCtrl-DMSO. Statistical significance was calculated using two-way ANOVA. Results represent three independent experiments.

These results are now presented in Appemdix Figure S2a and b and described in the text in lines 160-166.

In figure S1D, more repetitions of the entire experimental setup are required to conclude on GSH regulation by these factors. Standard deviations look like they were based on reading out the same samples, not representing independent experiments. The same concerns applies to Figs. S2D and S4B.

We thank the reviewer for the comments. While the experiments have been independently repeated in the original version, we still have revised the corresponding result panels with additional biological replicates, now shown in the following figures:

Appendix Figure S1g

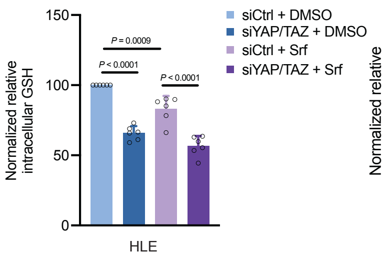

Appendix Figure S4d

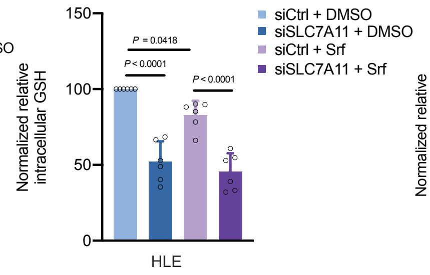

Appendix Figure S6e

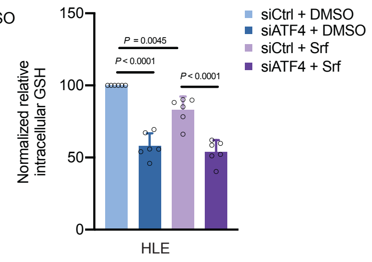

The western blots (loading controls) in Fig. 4d and 4e should be repeated.

Thank you for your suggestion. We have repeated the loading controls as suggested (see new Figures 4d and e).

Minor Remarks
• There is an antibody listed for cleaved caspase-3 but I cannot find an experiment where this is used. Please check for others!

We have removed the antibody from the antibody list and checked for the others.

Referee #3 (Comments on Novelty/Model System for Author):

For most experiments, the number of independent cell lines tested was limited. Suggestions have been included in my comments to the Authors.

Referee #3 (Remarks for Author):

Tang, Christofori and collaborators investigate mechanisms leading to sorafenib (Srf) resistance in Hepatocellular Carcinoma (HCC). By conducting an shRNA screen they identify YAP and TAZ as factors that, by inhibiting ferroptosis, confer resistance to Srf. They show that YAP/TAZ associate with ATF4 to regulate the expression of the cystine-glutamate antiporter SLC7A11. This proposed role for YAP-TAZ as inhibitors of ferroptosis is at odds with reports that demonstrate that YAP is an activators of ferroptosis (Wu et al, 2019, Yang et al. 2019, Yang et al. 2020).

The manuscript has potential novelty and impact provided that the Authors will be able to prove convincingly that, at least in HCC, YAP/TAZ are inhibitors of ferroptosis and that this leads to resistance to Srf.

Unfortunately, at this stage, the manuscript lacks coherence in the design of the experiments and consistency in the data shown: experiments need to be performed in multiple cell lines, in some instances the interpretation should be revised and additional experiments should be performed to reinforce their claims (see points below).

Also it needs to be clarified whether YAP/TAZ and ATF4 are inhibitors of ferroptosis selectively in Srf-resistant cells or whether elevation (or activation) of YAP/TAZ is sufficient to confer resistance in Srf-sensitive cells (or any cell).

We thank the reviewer for her/his interests in our manuscript and more importantly, for the helpful and constructive comments to improve the quality of the manuscript.

Major points.

1. Suppl figure 2E. As judged from the colony assay shown in Suppl. Figure 2E, HUH7 resistant cells appear to be sensitive to sorafenib (and not resistant as they should be). Similarly in figure 2h HLE cells seem to be extremely sensitive to Srf 6µM. Can the Authors provide data in support of the claim that these cells are resistant to Sorafenib? This is a key point, since all the manuscript is based on cell lines that should be resistant to Srf.

The IC50 values of different HCC cell lines have been reported in our previous publication (Tang et al., Nature Communications, 2019; Gao et al, Oncogenesis, 2021) which showed that HLE and SNU398 cells are partially resistant to Sorafenib treatment, while Huh7 and Hep3B cells are Sorafenib-sensitive HCC cell lines. The IC50 values of the HCC cell lines investigated, as well as of parental Huh7 and Hep3B cells and of the Sorafenib-resistant Huh7-IR and Huh7-CR and Hep3B-IR and Hep3B-CR cells, are now shown in Appendix Figure S1a-c and mentioned in the text in lines 79-84.

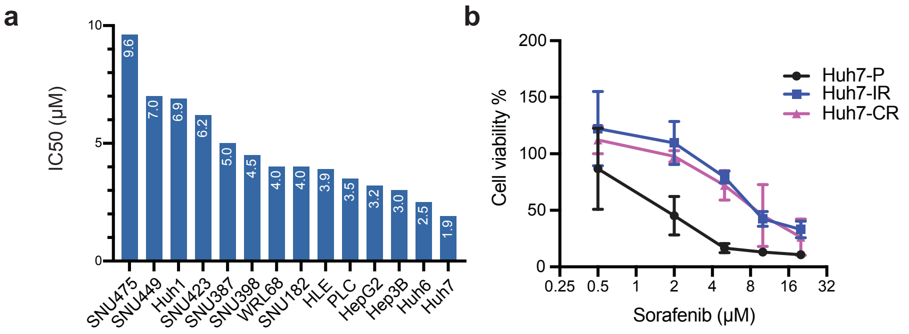

c

Cell line	IC50 (µM)
Huh7	1.7
Huh7-IR	10.7
Huh7-CR	10.8
Hep3B	3.0
Hep3B-IR	7.2
Hep3B-CR	8.3

(a) IC50 for Sorafenib responsiveness of different HCC cell lines. Different patient-derived HCC cell lines were treated with increasing doses of Sorafenib, and the IC50 values for cell growth inhibition by Sorafenib were determined. Hep3B and Huh7 were selected as two Sorafenib-responsive and HLE and SNU 398 as moderate Sorafenib-resistant HCC cell lines (taken from (Gao *et al*, 2021).
(b) Huh7-IR and Huh7-CR cells showing higher tolerance to Sorafenib than Huh7-parental cells. Cells were seeded into 96-well plate with 5000 cells/well, treated with increasing concentration of Sorafenib for 24 hours before harvest. Cell viability was measured with Promega CellTiter-Glo 2.0 kit and normalized to respective Sorafenib 0µM. (N = 4)
(c) Sorafenib IC50 values of HUH7, Huh7-IR, Huh7-CR and Hep3B, Hep3B-IR and Hep3B-CR. These IC50 values are fairly close to Sorafenib's clinically relevant concentration of 10µM.

2. Is the overexpression of YAP or TAZ sufficient to induce resistance to Sorafenib? Aside from the loss of function analysis in Srf-resistant cells, Authors should test whether over-expression of activated YAP (or TAZ) are sufficient to convey resistance to Srf and ferroptosis. Comparative evaluation in other cell lines (non-HCCs) will tell us whether this function of YAP-TAZ is only present in HCC lines.

We thank the reviewer for the comments.

As suggested by the reviewer, we expressed a constitutive-active version of YAP in Sorafenib-sensitive Huh7 and Hep3B cells and analyzed Sorafenib sensitivity in these cells. Indeed, we found that overexpressed active YAP can induce Sorafenib resistance as reflected by increased cell viability.

Furthermore, we overexpressed active YAP in MDA-MB231 cells and found that high levels of YAP can substantially restrict Sorafenib and RSL-3-induced ferroptosis in MDA-MB231 cells as well.

Together, the results demonstrate that YAP can promote ferroptosis resistance.

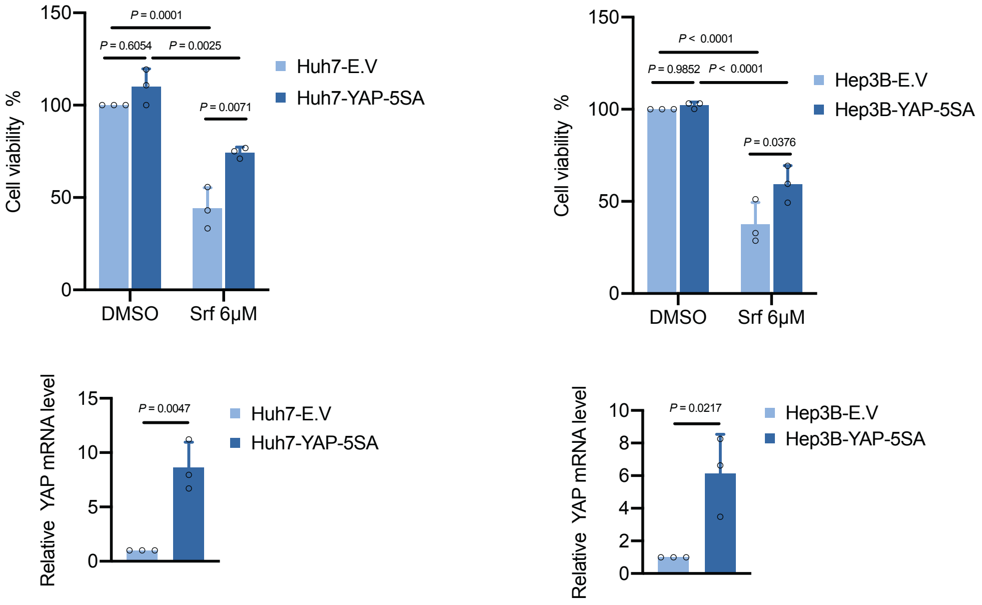

(Top panels) Activated YAP overexpression confers Sorafenib resistance in Huh7 and Hep3B cells. Huh7 and Hep3B cells transfected with empty vector (EV) or with a cDNA construct coding for YAP-5SA were cultured with either DMSO or 6μM Sorafenib (Srf) for 12 hours before harvest. Cell viability was measured with Promega CellTiter-Glo 2.0 kit and normalized to Huh7 and Hep3B transfected with empty vector (EV)

and treated with DMSO solvent. Statistical significance was calculated using two-way ANOVA. Results represent three independent experiments.

(Bottom panels) Quantitative RT-PCR analysis confirmed the overexpression of activated YAP. RNA was extracted from the cells described above and analyzed by quantitative RT-PCR. Statistical significance was calculated using Unpaired t-test. Results represent three independent experiments.

These results are now presented in Appendix Figure S3c and e and presented in the text in lines 169-174.

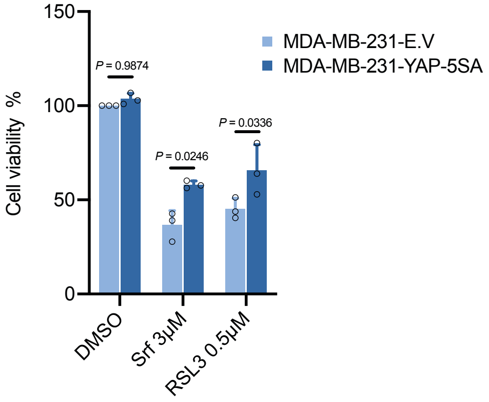

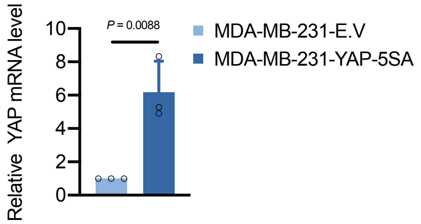

(Left panel) Activated YAP overexpression confers resistance to Sorafenib and RSL3 in MDA-MB-231 cells. MDA-MB-231 (empty vector control (EV) or YAP-5SA-expressing cells were treated with either DMSO or 3μM Sorafenib or 0.5μM RSL3 for 12 hours before harvest. Cell viability was measured with Promega CellTiter-Glo 2.0 kit and normalized to MDA-MB-23-EV + DMSO. Statistical significance was calculated using two-way ANOVA. Results represent three independent experiments.

(Right panel) Quantitative RT-PCR analysis confirmed the overexpression of activated YAP in MDA-MB-231 cells described in (d). Statistical significance was calculated using unpaired t-test. Results represent three independent experiments.

These data are now presented in Appendix Figure S8d and e and in the results section in lines 380-384.

3. What is the role of ATF4 and of the YAP/TAZ-TEAD-ATF4 complex in transcription and in the inhibition of ferroptosis? In particular the Authors should test whether overexpression of ATF4 alone will be sufficient to induced ferroptotic genes (SLC7A11, ATF3, CHAC1). This will clarify whether YAP/TAZ are dispensable for gene transactivation, but essential for ATF4 accumulation.

We thank the reviewer for the constructive comments. As suggested by the reviewer, we analyzed the effect of ATF4 overexpression on the expression of SLC7A11 and CHAC1.

Since in further experiments we did not observe robust changes in ATF3 expression, we did not further evaluate ATF3. Interestingly, while overexpression of ATF4 alone significantly induced the expression of SCL7A11 and CHAC1, the concomitant loss of YAP/TAZ reduced the ATF4-mediated induction of SLC7A11 and CHAC1 expression. This result is consistent with our findings that YAP/TAZ stabilize ATF4 and that YAP/TAZ and ATF4 collaboratively regulate the expression of SLC7A11.

These data are now presented in Appendix Figure S8a and described in the results section in lines 361-365.

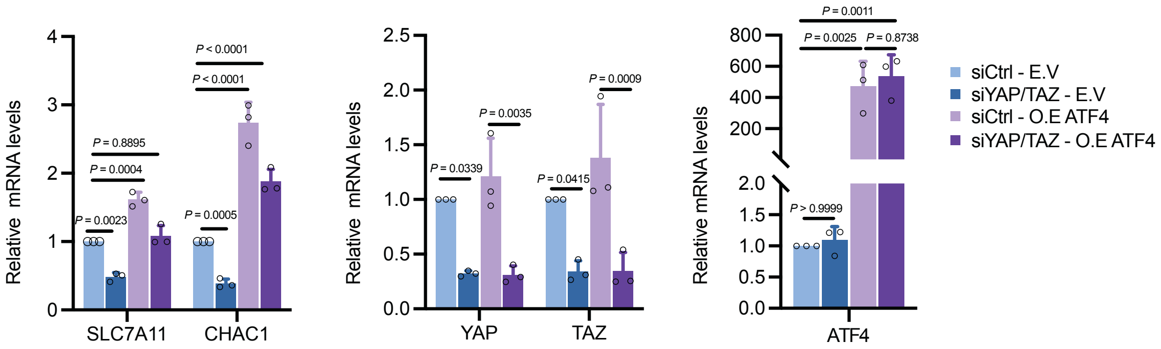

Quantitative RT-PCR analysis demonstrated that YAP/TAZ deficiency reduced ATF4-induced upregulation of *SLC7A11* and *CHAC1* gene expression. HLE cells were transfected with siCtrl or siYAP/TAZ, and 24 hours later transfected with empty vector or a plasmid construct encoding *ATF4*. RNA was extracted and analyzed by quantitative RT-PCR. *SLC7A11* and *CHAC1* gene expression significantly increased with overexpression of ATF4, but decreased significantly upon knockdown of YAP/TAZ (left). Knockdown efficiency of YAP/TAZ and overexpression of ATF4 were confirmed by quantitative RT-PCR (middle and right). Statistical significance was calculated using two-way ANOVA. Results represent three independent experiments.

4. The effect of ATF4 silencing should be tested using more than a single siRNA, in order to rule out off target effects.

We thank the reviewer for the constructive comment. As suggested, we repeated the experiment with two additional siRNAs and confirmed our conclusions with an expanded set of tools.

These data are now presented in Appendix Figure S6g and h and in the results section in lines 268-274.

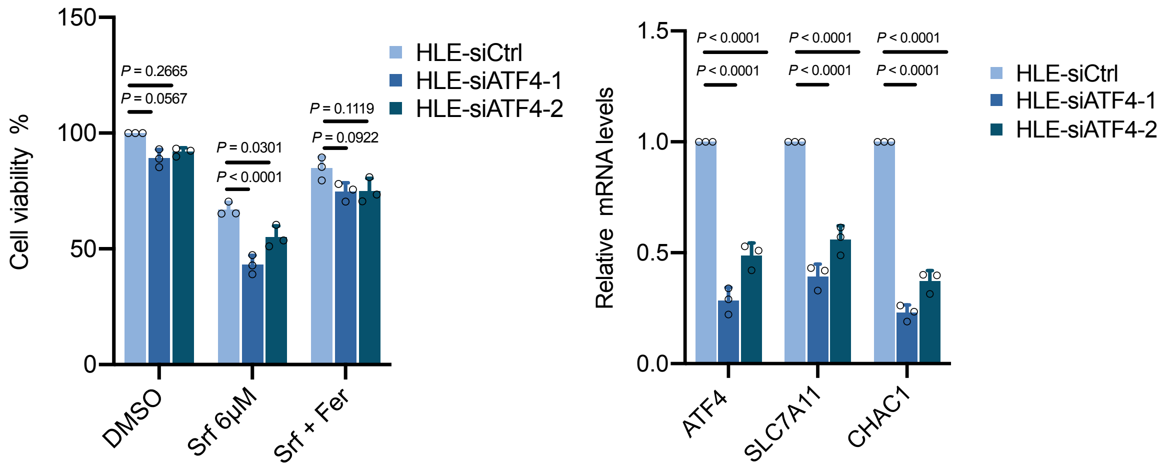

(Left panel) ATF4 deficiency induced cell death in response to Sorafenib which was overcome by treatment with Ferrostatin-1 (Fer). HLE cells were transfected with siCtrl, siATF4-1 or siATF4-2 and treated with 6µM Sorafenib with or without 5µM Ferrostatin-1 for 12 hours before harvest. Cell viability was measured with Promega CellTiter-Glo 2.0 kit and normalized to HLE-siCtrl DMSO. Statistical significance was calculated using two-way ANOVA. Results represent three independent experiments.

(Right panel) Quantitative RT-PCR analysis confirmed the knockdown efficiency of siATF4-1 and siATF4-2. RNA from the cells described in (g) was extracted and analyzed by quantitative RT-PCR. mRNA levels of *SLC7A11* and *CHAC1* decreased significantly with the deficiency of ATF4. Statistical significance was calculated using two-way ANOVA. Results represent 3 independent experiments.

5. Data in Figure 3 (and related text) show that ATF4 regulates SLC7A11 in response to Sorafenib treatment. This is a key evidence, the same experiment needs to be repeated for all the other Srf resistant lines.

We thank the reviewer for the comment. We further explored the effect of ATF4 in SLC7A11 expression in Huh7-IR and Huh7-CR cells. Indeed, also in these Sorafenib-resistant cell lines ATF4 was a key transcriptional factor regulating SLC7A11.

These data are now presented in Appendix Figure S6a and b and in the results section in lines 254-258.

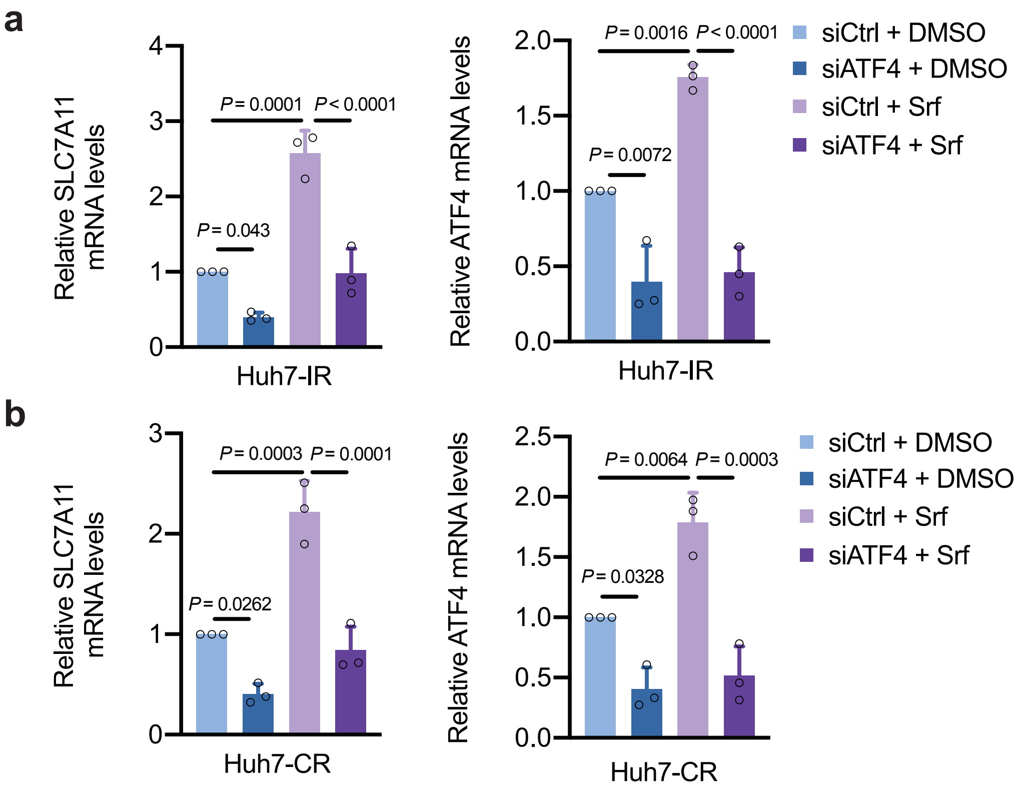

(a,b) *SLC7A11* mRNA levels were decreased upon ATF4 depletion in Sorafenib-resistant Huh7-IR and Huuh7-CR cells. Huh7-IR cells (a) and Huh7-CR cells (b) were transfected with siCtrl or siATF4 and cultured with DMSO or 6µM Sorafenib for 18 hours. Quantitative RT-PCR was used to determine *SLC7A11* (left) and *ATF4* (right) mRNA levels. *SLC7A11* decreased with siATF4 either under DMSO or Sorafenib treatment. Knockdown efficiency of siATF4 was determined by quantitative RT-PCR for ATF4 expression. Statistical significance was calculated using one-way ANOVA. Results represent three independent experiments.

6. Line 232. It is not clear how the Authors interpret the loss of viability following ATF4 silencing and how this relates to resistance to Srf. Is this sensitivity a trait acquired when cells became resistant to Srf? Are Srf-resistant cells more sensitive to ATF4 depletion than Srf-sensitive cells? If so, why? Is this because of "tonic" ER-stress signalling? Along with increased level of YAP/TAZ, have Srf-resistant cells also more ATF4?

We thank the reviewer for the constructive comment. To address this comment, we firstly probed the ATF4 expression in Sorafenib resistant cells. Interestingly, in line with an increase of YAP/TAZ in resistant cells, an upregulation of ATF4 has been observed in resistant cells in comparison to the sensitive counterpart as well.

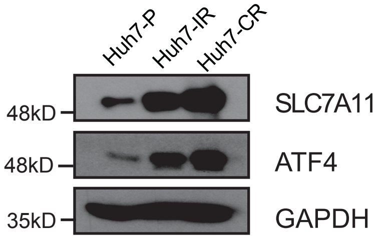

Immunoblotting of Sorafenib-resistant Huh7-IR and Huh7-CR cells revealed higher protein levels of ATF4 and SLC7A11 as compared to Sorafenib-sensitive parental Huh7 cells. GAPDH served as loading control.

The upregulation of ATF4 might be due to increased level of YAP/TAZ, which consequently facilitate the protein stability of ATF4, as demonstrated in our manuscript. With regard to ER-stress, Sorafenib is a well-known inducer of ER-stress (Rahmani *et al*, 2007; Shi *et al*, 2011).

This result is now shown in Appendix Figure S6c and mentioned in lines 258-262, We have lso included a discussion of this potential mechanism in the induction of ATF4 in the Discussion section in lines 445-459.

7. Figure 7H show that blocking ferroptosis by Ferrostatin-1 restores Sorafenib resistance in YAP/TAZ silenced HLE cells. The same rescue needs to be performed in HUH7 and Hep3B cells.

We thank the reviewer for this comment which is identical to Point 1 raised by Reviewer 1, and we refer the reviewer to our response to the reviewer there.

8. Loss of YAP/TAZ in HLE cells leads to increased lipid oxidation, suggesting that YAP/TAZ are implicated in the regulation of intracellular GSH. Is this only happening in Sorafenib resistant cells? Or alternatively YAP/TAZ regulates the intracellular red-ox also in Sorafenib sensitive cells?

We thank the reviewer for these comments. We have now overexpressed a constitutive-active version of YAP (YAP-5SA) in Sorafenib-sensitive Huh7 and Hep3B cells. In line with the increased viability of the cells in response to Sorafenib (see point 1 of Reviewer

1 and new Appendix Figure S3c), the forced expression of YAP-5SA also lead to upregulated intracellular GSH levels (updated Appendix Figure S3e).

These data are now shown in Appendix Figure S3e and mentioned in the text in lines 169-174.

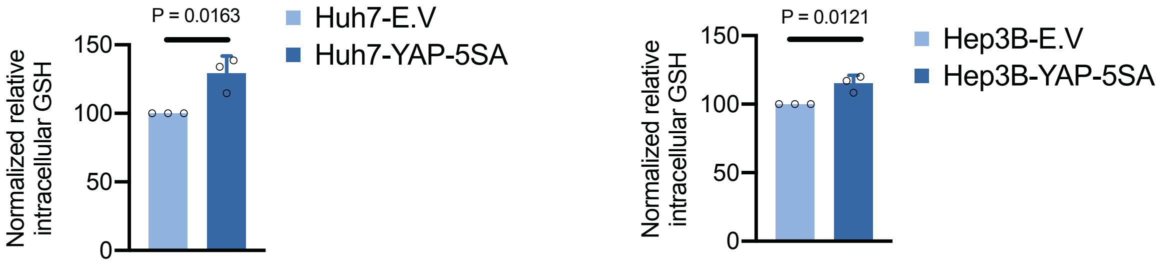

Intracellular GSH levels increased with the overexpression of activated-YAP (YAP-5SA). Huh7 and Hep3B cells were transfected with empty vector control (E.V) or with a plasmid coding for YAP-5SA. The cells were seeded into 96-well plates with 5000 cells/well, and the intracellular GSH levels were measured using the GSH-Glo Glutathione Assay kit and normalized to Huh7-EV and Hep3B-EV, respectively. Statistical significance was calculated using unpaired t-test. Results represent three independent experiments.

9. If activation of YAP/TAZ confers resistance to Srf by inhibiting ferroptosis, than Erastin should make resistant cells (i.e over-expressing YAP/TAZ) sensitive to Srf. This should be tested.

We thank the reviewer for the constructive suggestion to explore combination strategies. As suggested, we have now combined Sorafenib and Erastin in YAP-5SA-overexpressing Huh7 cells. As already described above, the forced activation of YAP made the initially Sorafenib-sensitive Huh7 and Hep3B cells more resistant to Sorafenib. This increased resistance as compared to empty vector control (E.V) cells was maintained in the presence of Erastin, although the overall rate of cell death was dramatically increased by Erastin, as expected.

These data are now shown in Appendix Figure S3f and described in the text in lines 175-181.

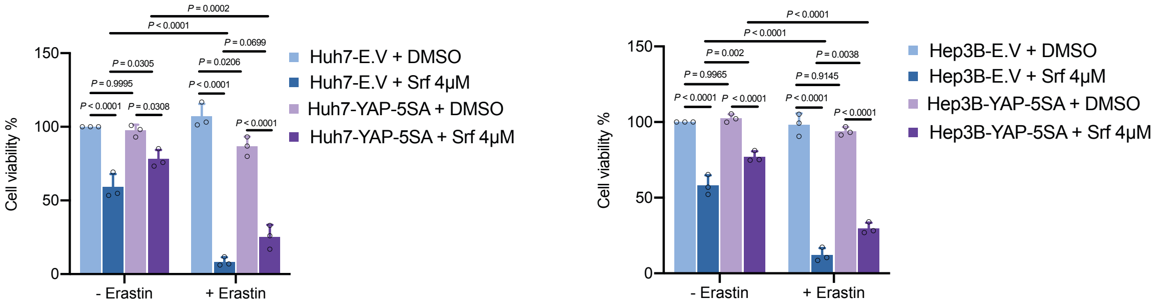

Combination of Sorafenib and Erastin induced higher rates of cell death as compared to single Sorafenib treatments in activated-YAP overexpressing Huh7 and Hep3B cells. Empty vector control (EV) transfected and YAP-5SA-expressing Huh7 and Hep3B cells were treated with DMSO or 4μM Sorafenib, combined with or without 2μM Erastin treatment for 12 hours before harvest. Cell viability was measured with Promega CellTiter-Glo 2.0 kit and normalized to empty vector-transfected Huh7/Hep3B treated with DMSO. Statistical significance was calculated using Two-way ANOVA. Results represent three independent experiments.

10. In vivo experiments were performed only on SNU398 cells. Oddly this line was not used in all the in-vitro experiments. For coherence and to strengthen the manuscript, Authors need to (i) show that SNU398 cells are Srf-resistant and that resistance is mediated by YAP/TAZ and ATF4, (ii) reinforce the in-vivo evidences by using additional Srf-resistant cell lines.

We thank the reviewer for the comments. We would like to note that from a previous study (Qiu *et al*, 2019)) and from our own experience we knew that most Sorafenib-resistant HCC cells did not readily form tumor upon transplantation into immunodeficient mice, with the exception of SNU398 cells.

Therefore, we initially tested both SNU398 cells and Huh7 and Hep3B-derived Sorafenib-resistant cell lines for tumor transplantation experiments in vivo. Unfortunately, tumor outgrowth post inoculation was extremely low for the Huh7 and Hep3B-derived Sorafenib-resistant cells. Therefore, we have chosen to employ SNU398 cells for the pharmacological interference studies in vivo.

To demonstrate that SNU398 cells are indeed a relevant model, we have now analyzed the functional contribution of YAP/TAZ and ATF4 for overcoming Sorafenib-induced ferroptosis in SNU398 HCC cells. Indeed, siRNA-mediated ablation experiments revealed that YAP/TAZ and ATF4 restricted Sorafenib-induced ferroptosis in SNU398 cells which could only be repressed by Ferrostain-1 and not by GSK872 or Z-VAD-FMK.

These data are now shown in Appendix Figure S9a and b and are described in the text in lines 390-396.

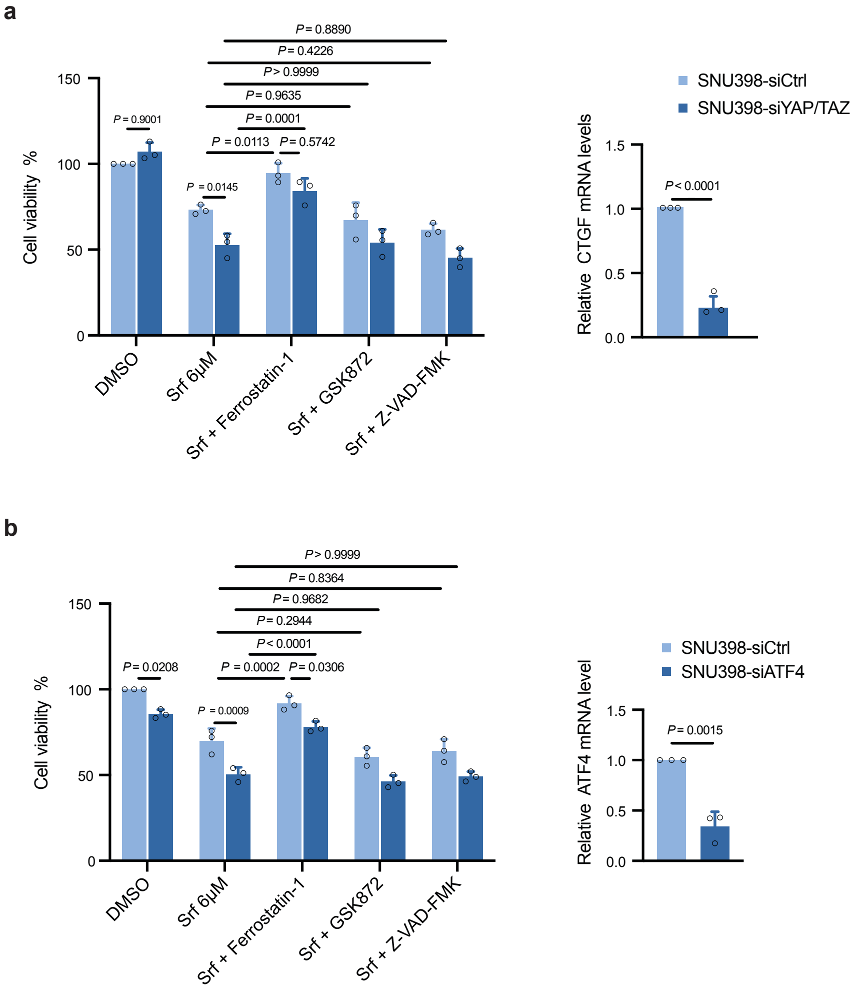

(a, b) Deficiency of YAP/TAZ induced higher rates of cell death in response to Sorafenib treatment in tumorigenic SNU398 cells, which could be rescued by Ferrostatin-1 but not by GSK872 or Z-VAD-FMK. SNU398 cells were transfected with either siCtrl or siYAP/TAZ and 24 hours later treated with 6μM Sorafenib with 5μM Ferrostatin-1, 10μM GSK872 or 10μM Z-VAD-FMK for 20 hours. Cell viability was measured with Promega CellTiter-Glo 2.0 kit and normalized to siCtrl-DMSO. Knockdown efficiencies of siYAP/TAZ and of siATF4 were determined by the mRNA expression levels of *CTGF*, a direct transcriptional target gene of YAP/TAZ, and of *ATF4* (right panels). Statistical significance was calculated using two-way ANOVA. Results represent three independent experiments.

11. Line 147: ChIP does not have the resolution to demonstrate binding to a transcription factor binding site. The PCR signal only shows that the immunoprecipitated chromatin contains the TEAD-binding motif, but the TF used for the IP could be binding

a nearby sequence contained in the immunoprecipitated DNA. The same holds for the sentence starting at line 308, concerning regulation by the AARE-motif, and the paragraph starting at line 312. Thus, the interpretation of these experimental evidences should be revised and in should not be used as an evidence for sequence specific binding

We thank the reviewer for this comment. We have now revised the text in the Results section to carefully and appropriately present and interpret this result (lines 228-230, 342-345, 349-351, and 356-3590.

Minor points

12. Figure 1C. TAZ seems to be modified upon Srf treatment as suggested by the altered migration in WB, have the Authors investigated this? This may reflect selective activation of TAZ (and possible YAP) upon Srf treatment.

We thank the reviewer for the comment. It has been well-known that YAP/TAZ are highly phosphorylated proteins, and phosphorylated YAP/TAZ migrate slower in a SDS-PAGE in comparison to non-phosphorylated proteins.
Indeed, Sorafenib can induce phosphorylation of YAP/TAZ as previously reported by our group (Tang *et al.*, 2019).

13. Are ATF4 level changing with cell density? (see fig. 4D)

Yes, we show in Figure 4d and describe in the text (lines 295-297) that ATF4 protein levels are influenced by cell density. We also show in Appendix Figure S5c and in the text in lines 217-221 that cell density affects the transcriptional activities of YAP and TAZ and with it the expression of CTGF, ANKRD1 and SLC7A11.

14. Line 204. This sentence reads: "Moreover, the forced expression of SLC7A11 in YAP/TAZ knock-down cells was able to prevent loss of YAP/TAZ-induced cell death in response to Sorafenib...." This sentence is difficult to read, if possible I kindly ask the Authors to amend it.

We have now reworded the sentence accordingly (now lines 235-237).

15. Line 219. To my knowledge the reference cited (Dixon, 2014) does not show that Sorafenib does not induce YAP/TAZ activity, as the Authors state here. Please clarify and if needed amend.

We apologize for our misleading reference. This has been a mishap. We have now corrected the reference to (Tang *et al.*, 2019).

References cited

Gao R, Buechel D, Kalathur RKR, Morini MF, Coto-Llerena M, Ercan C, Piscuoglio S, Chen Q, Blumer T, Wang X *et al* (2021) USP29-mediated HIF1alpha stabilization is associated with Sorafenib resistance of hepatocellular carcinoma cells by upregulating glycolysis. *Oncogenesis* 10: 52

Hiemer SE, Zhang L, Kartha VK, Packer TS, Almershed M, Noonan V, Kukuruzinska M, Bais MV, Monti S, Varelas X (2015) A YAP/TAZ-Regulated Molecular Signature Is Associated with Oral Squamous Cell Carcinoma. *Mol Cancer Res* 13: 957-968

Qiu Z, Li H, Zhang Z, Zhu Z, He S, Wang X, Wang P, Qin J, Zhuang L, Wang W *et al* (2019) A Pharmacogenomic Landscape in Human Liver Cancers. *Cancer Cell* 36: 179-193 e111

Rahmani M, Davis EM, Crabtree TR, Habibi JR, Nguyen TK, Dent P, Grant S (2007) The kinase inhibitor sorafenib induces cell death through a process involving induction of endoplasmic reticulum stress. *Mol Cell Biol* 27: 5499-5513

Shi YH, Ding ZB, Zhou J, Hui B, Shi GM, Ke AW, Wang XY, Dai Z, Peng YF, Gu CY *et al* (2011) Targeting autophagy enhances sorafenib lethality for hepatocellular carcinoma via ER stress-related apoptosis. *Autophagy* 7: 1159-1172

Tang F, Gao R, Jeevan-Raj B, Wyss CB, Kalathur RK, Piscuoglio S, Ng CK, Hindupur SK, Nuciforo S, Dazert EJNc (2019) LATS1 but not LATS2 represses autophagy by a kinase-independent scaffold function. *Nature communications* 10: 1-17

Zheng J, Sato M, Mishima E, Sato H, Proneth B, Conrad M (2021) Sorafenib fails to trigger ferroptosis across a wide range of cancer cell lines. *Cell Death Dis* 12: 698

15th Sep 2021

Dear Prof. Christofori,

Thank you for the submission of your revised manuscript to EMBO Molecular Medicine.
We have received the enclosed reports from the 3 referees who originally reviewed your manuscript, and as you will see, they are now supportive of publication. I am therefore pleased to inform you that we will be able to accept your manuscript once the following minor points will be addressed:

1/ Please address the remaining issues from referees #1 and #3.

2/ Main manuscript text:
- Please address the queries from our data editors in track changes mode in the main manuscript file labelled 'Data edited MS file'. Please use this file for any further modification and only keep in track changes mode the new modifications.
- Material and methods:
o We do not have limitations for Material and Methods, therefore please include the methods that are currently in the Appendix file in the main manuscript file.
o Antibodies: thank you for providing a table with antibodies information. Please also provide the dilutions used in the study.
o Cells: please indicate the culture conditions, and whether the cells were authenticated when applicable.
o Mice: please provide information on the origin of the mice, their age, gender, housing, and husbandry conditions. Please identify the institution that approved the experimentation.
- Please include the Data Availability section after the Material and Methods section. Please only include in the section the accession numbers and URL for the primary datasets produced in this study.
- Please merge the Funding section with the Acknowledgements section. Please make sure that the information provided in the submission system match the information provided in the manuscript.
- Author contributions: Michael Dill and Fernando Camargo are missing from this section.
- Please remove the Animal experiment approval section and include it in the Material and Methods section.
- Please move the section Patient material and ethics up, in the Material and Methods section. Please include a statement that informed consent was obtained from all subjects and that the experiments conformed to the principles set out in the WMA Declaration of Helsinki and the Department of Health and Human Services Belmont Report.
- References: Please reformat to have 10 authors listed before et al.

3/ Datasets and Appendix:
- There are 5 excel files uploaded as Appendix Table S1-5. Please rename "Dataset EV1" etc. All Datasets need their legends added in a separate tab. Please make sure that these datasets are referenced in the main manuscript text.
- Appendix: as mentioned above, please remove the Appendix methods section and include all methods in the main manuscript file. Please rename the Appendix tables "Appendix Table S1", etc.

4/ Thank you for providing Source Data. Please upload them as one file per figure, with blots and tables zipped where needed. The Source Data labelled Fig. 1d seems to refer to Fig. 1c, please check. Please also correct the labelling of source data for Appendix figures.

5/ Checklist:
Section D/8: please provide the age and gender of the mice
Section E/Human subjects: this section should contain the information provided in your manuscript under "patient material and ethics".

6/ Thank you for providing The Paper Explained section. I included minor modifications, please let me know if you agree with the following, or amend as you see fit:

PROBLEM
While treatment of liver cancer patients with Sorafenib, the current treatment of choice for advanced hepatocellular carcinoma, induces in most cases initial beneficial effects, resistance to Sorafenib therapy eventually occurs, tumors relapse, and patients succumb to the disease.
RESULTS
We investigated the molecular mechanism underlying the development and maintenance of resistance to Sorafenib therapy in liver cancer cells. We found that the transcriptional regulators YAP/TAZ and ATF4 cooperatively induce the expression of genes required for anti-oxidant pathways, which are critical to prevent cancer cell death by ferroptosis. These pathways are also upregulated in tumors of Sorafenib-resistant liver cancer patients. Proof-of-concept experiments with cultured liver cancer cells and in liver cancer mouse models revealed that inhibition of these pathways prevents the development of resistance to Sorafenib therapy.

IMPACT
These results suggest the possibility to re-sensitize therapy-resistant liver cancers to Sorafenib treatment by pharmacologically repressing the anti-oxidant pathways regulated by YAP/TAZ and ATF4.

7/ Thank you for providing a synopsis text. I added minor modifications to fit with our style and format, please let me know if you agree with the following:

Resistance to therapy occurs in most liver cancer patients treated with Sorafenib, and patients succumb to the disease. A synthetic lethal screen identified a regulatory circuit, which prevents ferroptosis and promotes cancer cell survival, thus promoting resistance to Sorafenib.

• The transcription factors YAP and TAZ stabilize ATF4 by promoting its nuclear import to cooperatively induce expression of SLC7A11, a cystine importer critical for glutathione synthesis.
• Glutathione synthesis and homeostasis are required to repress ferroptosis and to maintain Sorafenib resistance in liver cancer cells.
• Inhibition of Glutathione synthesis re-sensitizes Sorafenib-resistant cancer cells to Sorafenib therapy, which then induces ferroptosis and represses tumor growth in murine liver cancer models.
• Pharmacological repression of the anti-oxidant pathways regulated by YAP/TAZ and ATF4 could re-sensitize therapy-resistant liver cancers to Sorafenib treatment.

Please note that this would be the final version, and changes during proofing are usually not allowed.

Thank you for providing a nice synopsis picture. Please resize it as a PNG/TIFF file 550 px wide x 300-600 px high.

8/ As part of the EMBO Publications transparent editorial process initiative (see our Editorial at http://embomolmed.embopress.org/content/2/9/329), EMBO Molecular Medicine will publish online a Review Process File (RPF) to accompany accepted manuscripts.
This file will be published in conjunction with your paper and will include the anonymous referee reports, your point-by-point response and all pertinent correspondence relating to the manuscript. Let us know whether you agree with the publication of the RPF and as here, IF YOU WANT TO REMOVE OR any figures from it prior to publication.
Please note that the Authors checklist will be published at the end of the RPF.

I look forward to receiving your revised manuscript.

With kind regards,

Lise Roth

Lise Roth, PhD
Editor
EMBO Molecular Medicine

***** Reviewer's comments *****

Referee #1 (Remarks for Author):

The authors have satisfactorily addressed most of my concerns.
I would just like to point the authors a recent publication that would deserve mention and discussion in the framework of their study.
Sorafenib fails to trigger ferroptosis across a wide range of cancer cell lines.
Zheng J, Sato M, Mishima E, Sato H, Proneth B, Conrad M.
Cell Death Dis. 2021 Jul 13;12(7):698.

This study appears to convincingly show that sorafenib is unable to inhibit system Xc. The experiments are well controled and no inhibitions of system Xc- is shown. This could make it complex to rationalize the effect of sorafenib exclusively on the proposed function on system Xc-.

Referee #2:

Suitable for publication.

Referee #3 (Comments on Novelty/Model System for Author):

* as pointed put in my rebuttal, based on the new pharmacological data included in the new version of the manuscript, I do have some concerns regarding the use of HLE cells as "resistant" to srf.

Referee #3 (Remarks for Author):

I thank the Authors for taking the time to address the concerns I expressed in the first round of the revision. Following there are still a couple of issues that I think will need to be addressed.

Major points:

Point 1. I appreciate the Authors choice to resolve to a more quantitative and statistically meaningful way to display viability assay data: bar graphs are more informative than the pictures reporting representative colony assays. Yet, I still have some concerns about drug sensitivity data, and the definition of resistance (figS1a), since HLE cells, which are considered as resistant have an IC50 for srf which is 3.9 µM, not so greater than the IC50 of Hep3B (3.0 µM), which is a cell line reported as sensitive. It is thus unclear why in the present manuscript the Authors chose to focus on HLE cells (which do not seem really resistant to srf) and did not analyze cell lines like SNU475 which are clearly resistant to srf (IC50=9.6 µM).

Point 2. The Authors addressed whether activation of YAP rescued viability in sorafenib sensitive cells (upon sorafenib treatment). Yet, they did not provide data in support of the fact that this rescue was due to decreased ferroptosis. Indeed their sentence (line 184) "These results are consistent with a previous report by our laboratory demonstrating that the acute treatment of Sorafenib-sensitive HCC cells with Sorafenib induces autophagy and apoptosis which
is prevented by YAP/TAZ activities (Tang et al., 2019)." seems to suggest that the rescue is not due to ferroptosis, but either by Apoptosis and ferroptosis. Thus, I think that how YAP/TAZ may induce sorafenib resistance in srf sensitive cells is still an open question.

Point 3. Addressed

Point 4. Addressed

Point 5. Addressed

Point 6. Addressed

Point 7. Addressed

Point 8. Addressed

Point 9. Addressed

Point 10. Based on the Authors' response, I understand the Authors' reasons for using SNU398 cells for the in vivo experiments. For clarity, I think these arguments should be reported in the text.

Additional request: Please, double check the scale of the X axis of figS1, panel b, since it doesn't seem linear.

The authors performed the requested editorial changes.

4th Oct 2021

Dear Prof. Christofori,

Thank you for submitting the revised manuscript file. I am pleased to inform you that your manuscript is accepted for publication and is now being sent to our publisher to be included in the next available issue of EMBO Molecular Medicine.

Congratulations on your interesting work!

With kind regards,

Lise Roth

Lise Roth, Ph.D
Editor
EMBO Molecular Medicine

Follow us on Twitter @EmboMolMed
Sign up for eTOCs at embopress.org/alertsfeeds

EMBO PRESS

YOU MUST COMPLETE ALL CELLS WITH A PINK BACKGROUND

PLEASE NOTE THAT THIS CHECKLIST WILL BE PUBLISHED ALONGSIDE YOUR PAPER

Corresponding Author Name: Gerhard Christofori
Journal Submitted to: EMBO Molecular Medicine
Manuscript Number: EMM-2021-14351

USEFUL LINKS FOR COMPLETING THIS FORM

http://www.antibodypedia.com
http://1degreebio.org
http://www.equator-network.org/reporting-guidelines/improving-bioscience-research-repor

http://grants.nih.gov/grants/olaw/olaw.htm
http://www.mrc.ac.uk/Ourresearch/Ethicsresearchguidance/Useofanimals/index.htm
http://ClinicalTrials.gov
http://www.consort-statement.org
http://www.consort-statement.org/checklists/view/32-consort/66-title
http://www.equator-network.org/reporting-guidelines/reporting-recommendations-for-tumo
http://datadryad.org
http://figshare.com
http://www.ncbi.nlm.nih.gov/gap
http://www.ebi.ac.uk/ega
http://biomodels.net/
http://biomodels.net/miriam/
http://jjj.biochem.sun.ac.za
https://osp.od.nih.gov/biosafety-biosecurity-and-emerging-biotechnology/
http://www.selectagents.gov/

Reporting Checklist For Life Sciences Articles (Rev. June 2017)

This checklist is used to ensure good reporting standards and to improve the reproducibility of published results. These guidelines are consistent with the Principles and Guidelines for Reporting Preclinical Research issued by the NIH in 2014. Please follow the journal's authorship guidelines in preparing your manuscript.

A- Figures
1. Data
The data shown in figures should satisfy the following conditions:

- the data were obtained and processed according to the field's best practice and are presented to reflect the results of the experiments in an accurate and unbiased manner.
- figure panels include only data points, measurements or observations that can be compared to each other in a scientifically meaningful way.
- graphs include clearly labeled error bars for independent experiments and sample sizes. Unless justified, error bars should not be shown for technical replicates.
- if n< 5, the individual data points from each experiment should be plotted and any statistical test employed should be justified
- Source Data should be included to report the data underlying graphs. Please follow the guidelines set out in the author ship guidelines on Data Presentation.

2. Captions

Each figure caption should contain the following information, for each panel where they are relevant:

- a specification of the experimental system investigated (eg cell line, species name).
- the assay(s) and method(s) used to carry out the reported observations and measurements
- an explicit mention of the biological and chemical entity(ies) that are being measured.
- an explicit mention of the biological and chemical entity(ies) that are altered/varied/perturbed in a controlled manner.

- the exact sample size (n) for each experimental group/condition, given as a number, not a range;
- a description of the sample collection allowing the reader to understand whether the samples represent technical or biological replicates (including how many animals, litters, cultures, etc.).
- a statement of how many times the experiment shown was independently replicated in the laboratory.
- definitions of statistical methods and measures:
  - common tests, such as t-test (please specify whether paired vs. unpaired), simple $\chi 2$ tests, Wilcoxon and Mann-Whitney tests, can be unambiguously identified by name only, but more complex techniques should be described in the methods section;
  - are tests one-sided or two-sided?
  - are there adjustments for multiple comparisons?
  - exact statistical test results, e.g., P values = x but not P values < x;
  - definition of 'center values' as median or average;
  - definition of error bars as s.d. or s.e.m.

Any descriptions too long for the figure legend should be included in the methods section and/or with the source data.

In the pink boxes below, please ensure that the answers to the following questions are reported in the manuscript itself. Every question should be answered. If the question is not relevant to your research, please write NA (non applicable). We encourage you to include a specific subsection in the methods section for statistics, reagents, animal models and human subjects.

B- Statistics and general methods

Please fill out these boxes (Do not worry if you cannot see all your text once you press return)

1.a. How was the sample size chosen to ensure adequate power to detect a pre-specified effect size?	All experiments have been performed at n=3, except for animal experiments where the number of mice per treatment cohort was chosen to ensure statistical significance. For statistical analysis see Material and Methods sections and in legends to the figures.
1.b. For animal studies, include a statement about sample size estimate even if no statistical methods were used.	Immunodeficient NSG mice were used in numbers as described in the Methods sections "Tumor transplantation" and "PDX mouse models". 8 and 7mice per treatment cohort were used.
2. Describe inclusion/exclusion criteria if samples or animals were excluded from the analysis. Were the criteria pre-established?	No samples or animals were excluded from the analysis.
3. Were any steps taken to minimize the effects of subjective bias when allocating animals/samples to treatment (e.g. randomization procedure)? If yes, please describe.	The treatment protocols were compared to Placebo control in tumor transplanted mice. The isolated tumor samples were randomized and blinded after euthanasia of the mice for further histopathological investigations.
For animal studies, include a statement about randomization even if no randomization was used.	N/A
4.a. Were any steps taken to minimize the effects of subjective bias during group allocation or/and when assessing results (e.g. blinding of the investigator)? If yes please describe.	Tumor size measurement during experiment: no blinding. Histopathological and biochemical analyses of tumor biopsies: no knowledge of the scientist about group affiliation. See Methods section.
4.b. For animal studies, include a statement about blinding even if no blinding was done	No blinding was done, yet mice were supervised by animal care takers not involved in the actual experiment.
5. For every figure, are statistical tests justified as appropriate?	Yes. See Methods "statistical analysis" and individual figure legends.
Do the data meet the assumptions of the tests (e.g., normal distribution)? Describe any methods used to assess it.	Yes.
Is there an estimate of variation within each group of data?	Yes, variances are displayed in the individual figures by presenting all data points or relevant error bars.

Is the variance similar between the groups that are being statistically compared?	Yes, this is displayed within all individual figures.

C- Reagents

6. To show that antibodies were profiled for use in the system under study (assay and species), provide a citation, catalog number and/or clone number, supplementary information or reference to an antibody validation profile. e.g., Antibodypedia (see link list at top right), 1DegreeBio (see link list at top right).	Company and catalog number shave been provided wherever appropriate. See Materials and Methods section.
7. Identify the source of cell lines and report if they were recently authenticated (e.g., by STR profiling) and tested for mycoplasma contamination.	Materials and Methods. All cell lines were obtained from a neighboring laboratory and had been previously purchased from ATCC. All cell lines were routinely tested for Mycoplasma contamination by PCR analysis, and only non-contaminated cell lines were used for experiments.

* for all hyperlinks, please see the table at the top right of the document

D- Animal Models

8. Report species, strain, gender, age of animals and genetic modification status where applicable. Please detail housing and husbandry conditions and the source of animals.	Mus musculus, genetically engineered NOD/SCID;common gamma receptor knockout (NSG) mice as described in the Methods section "Tumor transplantation". 2-3 month-old male mice were used, due to the use of female mice for breast cancer experiments in the research group. Mice were housed and maintained under the suprvision of University of Basel Office of Animal Experimentation and by the Cantonal Veterinary Office of Basel-Stadt Nr. 2839.
9. For experiments involving live vertebrates, include a statement of compliance with ethical regulations and identify the committee(s) approving the experiments.	All experiments were performed following the rules and legislations of the Cantonal Veterinary Office and the Swiss Federal Veterinary Office (SFVO) covered by license Nr. 2839. See also Methods sections Methods sections "Tumor transplantation" and "PDX mouse models".
10. We recommend consulting the ARRIVE guidelines (see link list at top right) (PLoS Biol. 8(6), e1000412, 2010) to ensure that other relevant aspects of animal studies are adequately reported. See author guidelines, under 'Reporting Guidelines'. See also: NIH (see link list at top right) and MRC (see link list at top right) recommendations. Please confirm compliance.	Confirmed.

E- Human Subjects

11. Identify the committee(s) approving the study protocol.	All the analysis using human tissue samples reported in this study were approved by the ethics commission of Northwestern Switzerland (EKNZ, approval No.361/12).
12. Include a statement confirming that informed consent was obtained from all subjects and that the experiments conformed to the principles set out in the WMA Declaration of Helsinki and the Department of Health and Human Services Belmont Report.	Informed consent was obtained from all subjects and the experiments conformed to the principles set out in the WMA Declaration of Helsinki and the Department of Health and Human Services Belmont Report.
13. For publication of patient photos, include a statement confirming that consent to publish was obtained.	N/A
14. Report any restrictions on the availability (and/or on the use) of human data or samples.	Patient samples were available exclusively to the authors of this study and for the purpose of this study by the approval EKNZ, approval No.361/12.
15. Report the clinical trial registration number (at ClinicalTrials.gov or equivalent), where applicable.	N/A
16. For phase II and III randomized controlled trials, please refer to the CONSORT flow diagram (see link list at top right) and submit the CONSORT checklist (see link list at top right) with your submission. See author guidelines, under 'Reporting Guidelines'. Please confirm you have submitted this list.	N/A
17. For tumor marker prognostic studies, we recommend that you follow the REMARK reporting guidelines (see link list at top right). See author guidelines, under 'Reporting Guidelines'. Please confirm you have followed these guidelines.	N /A

F- Data Accessibility

18: Provide a "Data Availability" section at the end of the Materials & Methods, listing the accession codes for data generated in this study and deposited in a public database (e.g. RNA-Seq data: Gene Expression Omnibus GSE39462, Proteomics data: PRIDE PXD000208 etc.) Please refer to our author guidelines for 'Data Deposition'.<br><br>Data deposition in a public repository is mandatory for:<br>a. Protein, DNA and RNA sequences<br>b. Macromolecular structures<br>c. Crystallographic data for small molecules<br>d. Functional genomics data<br>e. Proteomics and molecular interactions	The sequencing data files are deposited on GEO database with the accession numbers: GSE117116 (RNA sequencing Sorafenib-resistant cell lines), GSE158458 (synthetic lethal DNA barcode sequencing) and GSE181771 (RNA sequencing of YAP/TAZ and ATF4 knockdown cells).
19. Deposition is strongly recommended for any datasets that are central and integral to the study; please consider the journal's data policy. If no structured public repository exists for a given data type, we encourage the provision of datasets in the manuscript as a Supplementary Document (see author guidelines under 'Expanded View' or in unstructured repositories such as Dryad (see link list at top right) or Figshare (see link list at top right).	N/A
20. Access to human clinical and genomic datasets should be provided with as few restrictions as possible while respecting ethical obligations to the patients and relevant medical and legal issues. If practically possible and compatible with the individual consent agreement used in the study, such data should be deposited in one of the major public access-controlled repositories such as dbGAP (see link list at top right) or EGA (see link list at top right).	N/A
21. Computational models that are central and integral to a study should be shared without restrictions and provided in a machine-readable form. The relevant accession numbers or links should be provided. When possible, standardized format (SBML, CellML) should be used instead of scripts (e.g. MATLAB). Authors are strongly encouraged to follow the MIRIAM guidelines (see link list at top right) and deposit their model in a public database such as Biomodels (see link list at top right) or JWS Online (see link list at top right). If computer source code is provided with the paper, it should be deposited in a public repository or included in supplementary information.	N/A

G- Dual use research of concern

22. Could your study fall under dual use research restrictions? Please check biosecurity documents (see link list at top right) and list of select agents and toxins (APHIS/CDC) (see link list at top right). According to our biosecurity guidelines, provide a statement only if it could.	N/A
